# Sharpening the DNA barcoding tool through a posteriori taxonomic validation: The case of *Longitarsus* flea beetles (Coleoptera: Chrysomelidae)

**DOI:** 10.1371/journal.pone.0233573

**Published:** 2020-05-21

**Authors:** Daniele Salvi, Emanuele Berrilli, Paola D’Alessandro, Maurizio Biondi

**Affiliations:** 1 Department of Health, Life and Environmental Sciences, University of L’Aquila, Coppito, L’Aquila, Italy; 2 CIBIO-InBIO, Centro de Investigação em Biodiversidade e Recursos Genéticos, Universidade do Porto, Vairão, Portugal; Universita degli Studi di Roma La Sapienza, ITALY

## Abstract

The accuracy of the DNA barcoding tool depends on the existence of a comprehensive archived library of sequences reliably determined at species level by expert taxonomists. However, misidentifications are not infrequent, especially following large-scale DNA barcoding campaigns on diverse and taxonomically complex groups. In this study we used the species-rich flea beetle genus *Longitarsus*, that requires a high level of expertise for morphological species identification, as a case study to assess the accuracy of the DNA barcoding tool following several optimization procedures. We built a *cox1* reference database of 1502 sequences representing 78 *Longitarsus* species, among which 117 sequences (32 species) were newly generated using a non-invasive DNA extraction method that allows keeping reference voucher specimens. Within this dataset we identified 69 taxonomic inconsistencies using barcoding gap analysis and tree topology methods. Threshold optimisation and *a posteriori* taxonomic revision based on newly generated reference sequences and metadata allowed resolving 44 sequences with ambiguous and incorrect identification and provided a significant improvement of the DNA barcoding accuracy and identification efficacy. Unresolved taxonomic uncertainties, due to overlapping intra- and inter-specific levels of divergences, mainly regards the *Longitarsus pratensis* species complex and polyphyletic groups *L*. *melanocephalus*, *L*. *nigrofasciatus* and *L*. *erro*. Such type of errors indicates either poorly established taxonomy or any biological processes that make mtDNA groups poorly predictive of species boundaries (e.g. recent speciation or interspecific hybridisation), thus providing directions for further integrative taxonomic and evolutionary studies. Overall, this study underlines the importance of reference vouchers and high-quality metadata associated to sequences in reference databases and corroborates, once again, the key role of taxonomists in any step of the DNA barcoding pipeline in order to generate and maintain a correct and functional reference library.

## Introduction

DNA barcoding is a molecular method of specimen identification using a short segment of DNA from a specific standardized gene which is compared against a database of known sequences from morphologically identified specimens. Therefore, the intent of the DNA barcoding is to standardize the large-scale screening use of one or more reference genes in order to assign unknown individuals to species [1–3]. The two key premises on which barcoding is based are: i) the nucleotide sequence used is characterized by a genetic divergence between close species that exceeds variation within the species; ii) the presence of a comprehensive sequences library obtained from individuals reliably determined at species level by expert taxonomists [4, 5]. Respecting these premises, DNA barcoding promises to be a fast and useful tool for taxonomists and a cost-effective system through which non-specialist can assign unidentified specimens to known species [6].

In recent years, large-scale DNA barcoding studies has been performed on various groups of animals and have generated an enormous amount of cytochrome oxidase I (*cox1*) barcodes, which are usually stored in GenBank^®^ and the official Barcode of Life database (BOLD) [7–9]. The association of such amount of sequences to taxa is a challenging step of these studies, especially for extraordinarily diverse group such as insects [10]. Indeed, for the most diverse orders (e.g. Diptera, Hymenoptera and Coleoptera), correct identification of species requires a high number of taxonomists, each one specialized on a single family or part thereof. Species-level identification represents a great challenge in some hyper-diverse and widespread genera, for which many taxonomists, each one with a long-standing taxonomic specialization on a regional fauna, might be required [11, 12]. This implies that broad-based DNA barcoding studies should ideally recruit hundreds of specialised taxonomists, but this is not feasible. Thus, a certain degree of misidentification is inherent to these studies, and can be anticipated in species-rich taxa with difficult taxonomy [13].

The inclusion of wrongly identified sequences into the reference databases undermines one of the key premises of the DNA barcoding tool and reduces its accuracy [14, 15]. Indeed, these misidentified sequences generate taxonomic inconsistencies, either because they fix a wrong species tag, if they represent species new to BOLD (or any other reference database), or because they will cause incongruence with data that already exists in these databases. Taxonomic inconsistencies within reference databases can be considered as *extrinsic errors* of the DNA-barcoding tool and can be afterwards detected, revised and corrected. For this purpose, non-invasive methods of DNA extraction that allow to keep reference samples in collection for further morphological validation [16–22] and high-quality metadata associated to submitted sequences (e.g. voucher type, date of collection, geographic coordinates, ecological information, images, etc.) are fundamental requirements for *a posteriori* revisions of the identification [23–26]. Revisions can be directly targeted to the instances of taxonomic inconsistency that occur in large dataset, previously identified through bioinformatic analyses. A variety of tools is available for detecting taxonomic inconsistencies both before and after deposition in the global barcode library. A first approach is based on threshold clustering and assumes that the intra-specific nucleotide variability of sequences does not exceed a certain distance value, otherwise sequences are flagged as belonging to different species [13, 27–29]. This method is implemented in the BOLD platform with a standard threshold of 1% [28]. However, there is no a priori reason to assume a threshold with a prescribed limit [30–32]. The recognition of a “boundary" among species will vary considerably due to differences in rate of nucleotide substitution and speciation time [33, 34]. Establishing robust thresholds for species delimitation is a key component of the barcoding process. Therefore, use of software and protocols to generate an optimised threshold directly from the data is a more effective procedure [23, 35–37]. Other approaches aimed at detecting taxonomic inconsistencies in reference databases consider topological incongruence between the taxonomic and the phylogenetic tree as an indication that some of the sequences might be mislabelled [38, 39]. Furthermore, the automated tool TAxCI, that combines multiple approaches for flagging and filtering inconsistent cases of specimen’s taxonomy has been recently developed [40].

Accuracy of the DNA barcoding also depends on the extent of the so-called barcoding gap, i.e. the separation between intraspecific variation and interspecific divergence estimated on the basis of the selected DNA marker, e.g. the mitochondrial *cox1* gene fragment in the case of animals [41]. However, poorly established taxonomy [42] as well as many biological processes including recent speciation [43, 44], species-level polyphyly [45], interspecific hybridisation [46–48], horizontal gene transfer mediated by bacterial endosymbionts [49, 50] make mtDNA groups poorly predictive of species boundaries thus affecting the accuracy of the DNA barcoding tool. These circumstances in which the molecular identification tool loses sensitivity, even in the presence of an error-free dataset, can be considered as an *intrinsic error* of the barcoding method. Identifying those areas of the dataset in which this type of error is present allows us to know which are those species or species groups that need to be analysed with more powerful integrative approaches to delimit, discover and identify species [51].

Here we used the hyper-diverse and taxonomically complex genus *Longitarsus* Latreille (Coleoptera, Chrysomelidae, Galerucinae, Alticini) as a case study to assess the accuracy of the DNA barcoding tool following several optimization procedures. Alticini is a tribe of small to medium-sized Coleoptera named ‘flea beetles’ because of their ability to jump due to the presence of a metafemoral extensor tendon in the swollen hind femora [52]. *Longitarsus* is the most abundant genus among flea beetles, with over 700 species distributed in all zoogeographical regions. Larvae and adult feed respectively on roots and leaves of plants of different angiosperm families, with levels of trophic specialization ranging from strictly monophagous to widely polyphagous [53]. Members of the genus are small-sized, with body length generally 2 to 4 mm. They can be recognized mainly by the co-occurrence of elongate first metatarsomere, exceeding half-length of hind tibia, confuse elytral punctuation, and absence of dorsal pubescence. Many species of this flea beetle genus are often part of morphologically homogenous species groups displaying striking similarities in external morphology, so a careful examination of the internal anatomic structures, mainly aedeagus and spermatheca, are also required to group specialists for reliable species identification [54].

The main aims of this study are: (i) to identify taxonomic inconsistencies within the *cox1* reference database available for *Longitarsus* using barcoding gap analysis, inclusive threshold specimen identification analysis, and tree topology methods; (ii) to implement *a posteriori* taxonomic revision of ambiguous and incorrect sequences using newly generated sequences identified by *Longitarsus* specialised taxonomists and metadata obtained for sequences already in these databases; and (iii) to assess the effect of these bioinformatic and taxonomic procedures on the identification efficacy of the DNA barcoding tool. Furthermore, by resolving the *extrinsic errors* within the reference database of *Longitarsus* during steps (i) and (ii), we will identify the cases where *intrinsic errors* due to taxonomic uncertainty or specific biological processes are likely to occur, thus providing directions for integrative taxonomic and evolutionary studies on this group.

## Materials and methods

### Ethics statement

Specimens analysed in this study belong to flea beetles (Coleoptera, Chrysomelidae, Galerucinae, Alticini) and have been collected in Italy and Portugal. No species of Alticini are listed as endangered or protected and their collection is not subjected to restriction by national and international laws and does not require special permission. Since the study did not involve laboratory work on living animals, authorization from the Ministry of Health was not required.

### Sample collection and morphological identification

**Fig 1:**
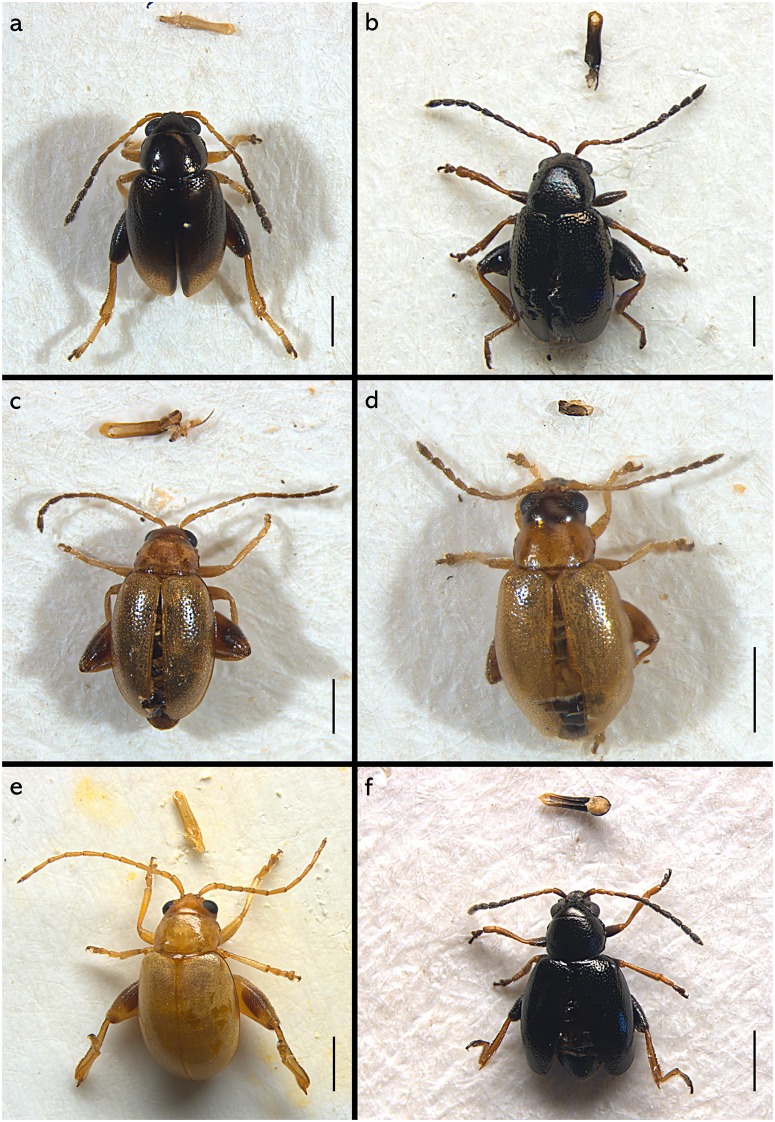
Photographs of voucher specimens of some of the species sequenced in this study (see S1–S3 Figs for photographs of the remaining species). Habitus and aedeagus or spermatheca of (a) *Longitarsus aeneicollis* ♂; (b) *L*. *corynthius metallescens* ♂; (c) *L*. *ballotae* ♂; (d) *L*. *pratensis* ♀; (e) *L*. *candidulus* ♂; (f) *L*. *anchusae* ♂. Scale bar 0.5 mm.

**Fig 2:**
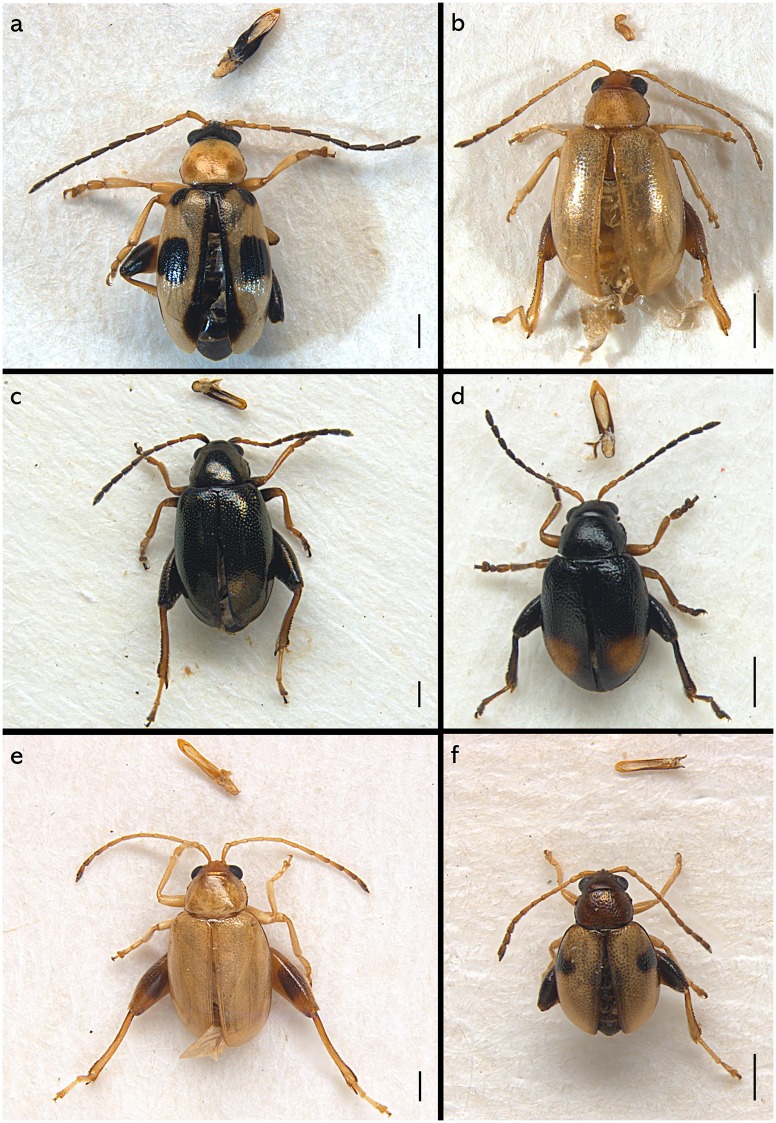
Photographs of voucher specimens of some of the species sequenced in this study (see S1–S3 Figs for photographs of the remaining species). Habitus and aedeagus or spermatheca of (a) *Longitarsus isoplexidis* ♂; (b) *L*. *pellucidus* ♀; (c) *L*. *echii* ♂; (d) *L*. *holsaticus* ♂; (e) *L*. *foudrasi* ♂; (f) *L*. *lateripunctatus* ♂. Scale bar 0.5 mm.

*Longitarsus* specimens analysed in this study were collected from their host plant by sweep net and the aid of aspirator and then stored in 95% ethanol. All specimens were morphologically identified by Maurizio Biondi at the species level with the auxiliary use of a Leica M205C binocular microscope. For each identified species we selected from 3 to 4 specimens, from the same locality, for DNA extraction. Among these specimens, before the DNA extraction, one specimen was mounted on an entomological card point with aedeagus or spermatheca after dissection and photomicrographs were taken using a Leica DFC500 camera and the Zerene Stacker software version 1.04. Scanning electron micrographs were taken using a Hitachi TM-1000camera (Figs 1 and 2 and S1, S2 and S3 Figs).

### DNA extraction, amplification, and sequencing

We used two different DNA extraction methods: (i) an invasive method, which involves the use of the entire specimen for DNA extraction, and (ii) a non-invasive method, which involves the separation of the head-prothorax portion of the animal from the rest of the body with the use of a entomological pin and the immersion of the two parts directly in lysis buffer and proteinase K. However, the non-invasive method, precisely because it allows to keep a specimen reference voucher, has been used more than the invasive method (84% of the samples were treated with a non-invasive method). In both cases the total DNA extraction was performed using a standard high-salt protocol [55]. The samples treated with the non-invasive method were recovered when the lysis process was completed, and the two parts of the animal were reassembled on an entomological card point. The standard barcode region of the mitochondrial *cytochrome c oxidase I* (*cox1*) gene (658 bp) was amplified by PCR using the primers specifically designed for *Longitarsus* Lon-LCO-F (5’-CTC AGC CAT TTT ACC GAA TAA ATG-3’) and LonHCO-R (5’-GGA TTT GGI ATA ATT TCY CATA TTG-3’) [53]. Amplification was carried out in a total volume of 25μl, with 12,5μl of BioMix^™^ 2x (Bioline Ltd, London, UK), 0.5 μl of each primer (10mM), 0.5 μl of BSA, and 1 μL (~40 ng) of DNA template. PCR cycling conditions for *cox1* followed [56]. Successful amplification was determined by gel electrophoresis and PCR products were purified and sequenced by an external service (Genewitz, UK). The obtained chromatograms of each sequence were manually edited and assembled into a consensus sequence using Geneious R8 (Biomatters Ltd., Auckland, New Zealand); consensus sequences were deposited in BOLD and GenBank database (BOLD accession number: BARLG001-20—BARLG117-20; GenBank accession: MT372331—MT372441).

### Reference sequence dataset building

We built a non-redundant database including all sequences of cytochrome genes of *Longitarsus* available in the public repositories of GenBank and BOLD (data updated to 12/08/2019). We downloaded 1372 sequences from GenBank and 1433 sequences from BOLD. For sequence mining we use “Longitarsus cytochrome” as search query in the GenBank nucleotide database, and “Longitarsus” as search query in the Public Data Portal of BOLD. We eliminated all retrieved sequences that were not identified to species level (94 sequences from GenBank and 16 from BOLD). We used the *duplicated ()* function [57] of R studio to dereplicate the dataset by removing sequences having identical GenBank accession number. Before removing redundant sequences, we checked cases in which the same GenBank accession number was associated with two different specific names in BOLD and GenBank^®^. In these cases, we retained the more recently updated name. The non-redundant cytochrome sequences database built following this procedure includes 1429 sequences, to which we added 117 newly generated *cox1* sequences for 32 *Longitarsus* species, for a total of 1546 sequences. To select only those sequences corresponding to *cox1* barcoding fragment, we assembled the 1546 sequences using the *map to reference* option in Geneious R8, and we trimmed the assembled dataset, using as reference the *cox1* sequence that we generated for *Longitarsus pratensis* voucher ‘6c’ using standard *cox1* barcode primers [41]. Afterwards, a multiple sequence alignment was performed with MAFFT v.7 using the FFT-NS-1 progressive method algorithm [58] and we eliminated two sequences that were shorter than 300 base pairs (bp). The final *cox1* dataset used for downstream analyses included 1502 sequences representing 78 *Longitarsus* species.

### Sequences’ taxonomy assessment analyses

The R library *ape* v5.3 [59] was used to calculate a pairwise distance matrix of intraspecific and interspecific genetic distance using the Kimura-two parameters (K2P) substitution model [60] with the pairwise deletion option. With the R package *spider* v1.5.0 [35] we performed the Barcoding Gap analyses [23] by estimating two statistics for each individual sequence in the dataset: (i) the *maximum intraspecific distance* (i.e., the maximum value of genetic distance between each sequence of the dataset with sequences of the same named species) and (ii) the *minimum interspecific distance* (i.e., the minimum value of genetic distance between each sequence of the dataset with sequences of different named species). When the difference between the maximum intraspecific distance and the minimum interspecific distance is equal to or less than zero, it means that there is the absence of a barcoding gap. For each species, we counted the instances of absence of the barcoding gap using a linear model and a kernel density estimate (KDE) developed in R library *ggplot2* [61]. We set the KDE method using a gaussian kernel function and the default smoothing bandwidth parameter of *ggplot2*. To assess the effect of limited sampling on the barcoding gap analyses we plotted the number of absences of barcoding gap against the total number of sequences available for each species [23, 62].

The distance threshold is a key parameter for barcoding analyses. We performed a threshold optimisation analysis in *spider* in order to calculate the value of genetic distance which reduces the number of identifications error. The best threshold was identified with the *localMinima* function that is based on the concept of the barcoding gap and identifies a dip in the density of genetic distances as a transition between intra- and inter-specific distances. This function does not require prior knowledge of species identity to get an indication of potential threshold values. To reduce the negative effects of poor taxon coverage, we removed singletons (i.e., species represented by a single sequence) from the dataset [27, 63].

The efficiency of molecular identification, before and after threshold optimization and singleton removal, was assessed using two methods: Best Close Match analyses [27] and the *TaxCI* pipeline developed by Rulik et al. [40]. The first method compares each sequence with the other sequences included in the dataset and checks if the smallest genetic distance (i.e. best match *sensu* Meier [27]) are between sequences tagged with the same species name. The TaxCI method identifies taxonomic inconsistencies based on tree topology. For this analysis a Neighbour-Joining (NJ) tree was inferred using the K2P model in MEGA7 [60].

Finally, we performed a taxonomic revision of all those sequences identified as wrong or ambiguous in the previous steps. The taxonomic revision was based on: (i) comparison with our newly generated reference sequence for 34 *Longitarsus* species; (ii) available metadata associated with sequences deposited in BOLD; (iii) newly generated metadata for voucher specimens kindly loaned to us by authors of sequences deposited in BOLD. Following this *a posteriori* taxonomic revision, incorrect identifications were corrected and the identification accuracy of the barcoding tool based on the resulting reference database was assessed using the same procedures described above (Barcoding Gap analyses, threshold optimization analyses, Best Close Match and *TaxCI* analyses). We used the ANOVA to test for differences between correct species identification ratio obtained with (i) the original reference dataset, (ii) the dataset with the optimised threshold, and (iii) the final reference dataset after the taxonomic revision. The ANOVA test was performed in the R library *clusterSim* after the data were normalized by quotient transformation (x/mean) [64].

## Result

Barcoding gap analysis and tree topology methods show that DNA barcoding accuracy and identification efficacy of the non-redundant database of *cox1* sequences (*Original dataset*) were improved after threshold optimization (*Optimal Threshold Dataset*) and *a posteriori* taxonomic revision (*Final dataset*).

### Original dataset

**Fig 3:**
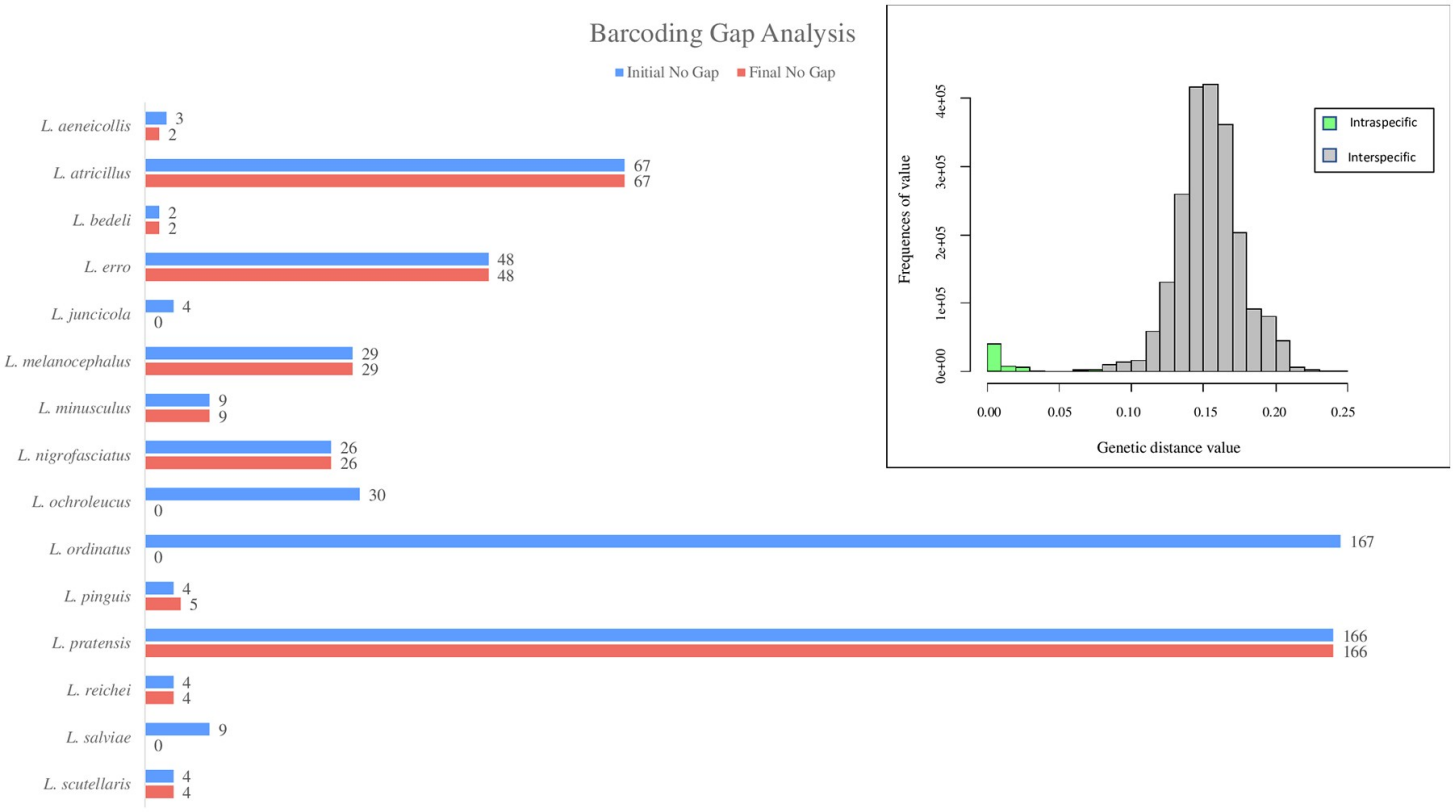
Results of the barcoding gap analyses for the original dataset and the final dataset. The number of absences of barcoding gap for each species is reported.

**Fig 4:**
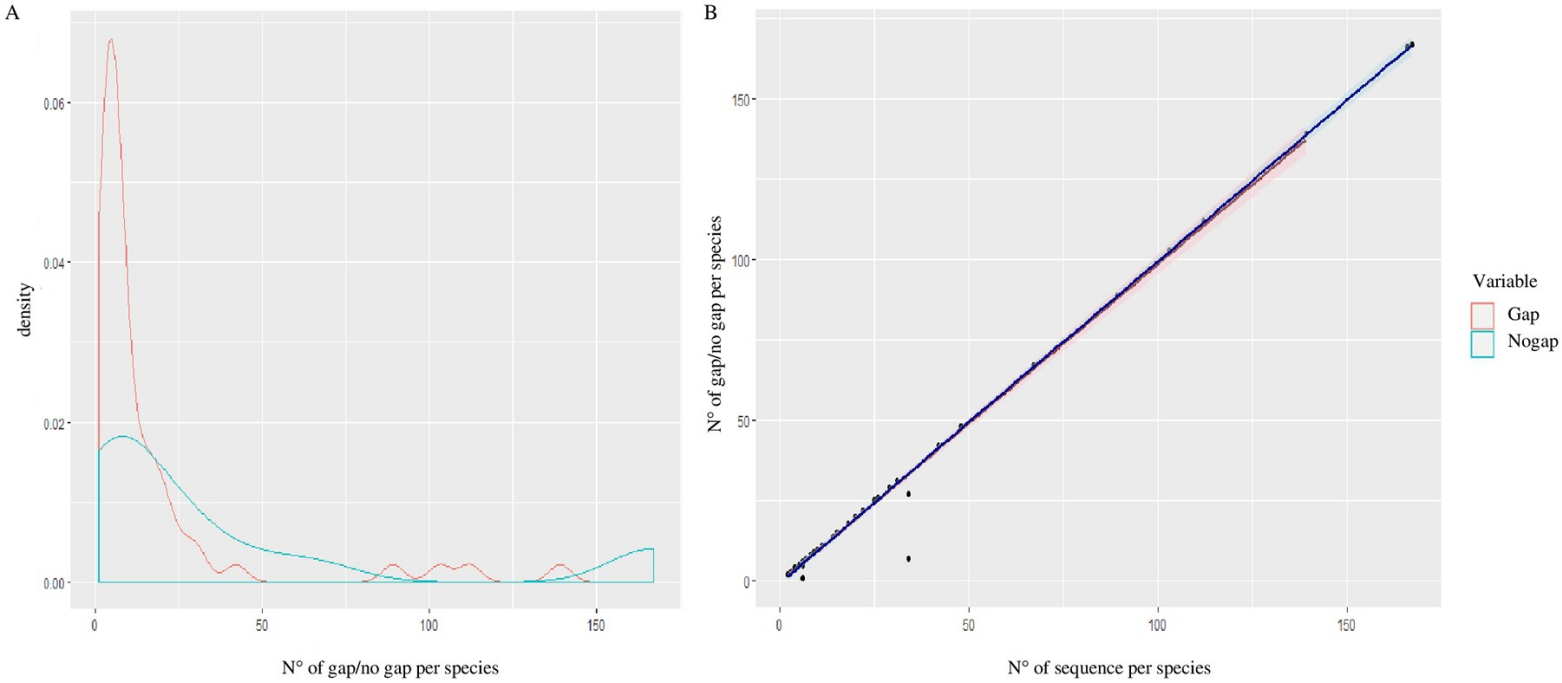
Effect of sequence sampling on the barcoding gap analyses. (a) Kernel Density Plots showing the distribution of instances of absence of the barcoding gap over the total number of sequences available for each species. (b) Linear regression models showing the association between the total number of sequences available for each species and the number instances of presence (red) and absence (blue) of barcoding gap.

The *cox1 Longitarsus* dataset includes 1502 sequences unevenly distributed among 78 species, which corresponds to ~ 11.1% of the described species diversity of the genus. The most represented species was *L*. *ordinatus* (Foudras, 1860) with 167 sequences, whereas 32 species were represented by less than 5 sequences. We found an overlap between the distribution of intra- and inter-specific pairwise K2P distances, resulting in the absence of an evident barcode gap in the *Longitarsus* datasets (Fig 3). Intraspecific K2P distance values ranged from 0 to 17.7% (mean = 1.4%). The maximum intraspecific value was observed among three sequences belonging to *L*. *erro* Horn, 1889, all collected in Canada. Interspecific K2P distances ranged from 0 to 27.6% (mean = 15.4%). A value of interspecific distance equal to 0 was found in several comparisons between sequences tagged as different species. The difference between the *maximum intraspecific distance* and the *minimum interspecific distance* calculated for each sequence resulted in 573 cases in which the barcoding gap is not present (38.2% of sequences) (Fig 3). The identification of a barcoding gap can be biased by reduced sampling of nucleotide variability at an inter- and intra-specific level [23, 62]. In this study, the presence of the barcoding gap was not associated to the number of sequences per species (Fig 4). The linear regression model shows an increase both in the absence (adjusted *R*^*2*^ = 0.9989, *p*-value = 2.2e-16) and in the presence (adjusted *R*^*2*^ = 0.9828, *p*-value = 2.2e-16) of the barcoding gap with the increase in the number of sequences per species (Fig 4B). Both the number of absences and presences of the barcoding gap have a higher density estimate in species represented by ~ 10 sequences (Fig 4A).

The barcoding identification efficiency on the original *Longitarsus* dataset evaluated through the best close match analysis, with the default 1% distance threshold, resulted in 95.2% of correct identification (1431 out of 1502). Remaining sequences resulted in: 36 ambiguous sequences, presenting more than one species as the closest match or within the distance threshold; 10 incorrect sequences, which present a different species as their closest match and 25 no id sequences that do not have a close match within the given threshold. It should be noted that all the ambiguous and incorrect sequences represent cases in which the barcoding gap is not present (Table 1). Results of the TaxCI analysis are overall in line with the other analyses and identified 28 heterospecific cluster of which 13 have sequences found in more than one cluster. Furthermore, TaxCI identified some new cases of inconsistent identification as reported in Table 2 (see also S6 Fig).

**Table 1:** Results of the best close match analyses. Taxonomic inconsistency for each species are reported as minimum intraspecific (Min inter dist) and maximum interspecific (Max intra dist) genetic distance, and the number of correct, ambiguous, incorrect, and non-identify (No id) sequences, for the original dataset, the optimal thresholds dataset, and final dataset.

			Original Dataset				Optimal Threshold Dataset				Final Dataset			
Species	Min inter dist	Max intra dist	Correct	Ambiguous	Incorrect	No id	Correct	Ambiguous	Incorrect	No id	Correct	Ambiguous	Incorrect	No id
*Longitarsus atricillus*	0.2	6.3	64	0	1	2	66	0	1	0	66	0	0	0
*Longitarsus bedeli*	0.2	0.9	0	0	2	0	0	0	2	0	0	0	2	0
*Longitarsus brisouti*	13.8	1.4	2	0	0	1	3	0	0	0	3	0	0	0
*Longitarsus isoplexidis*	12.0	1.7	4	0	0	1	5	0	0	0	5	0	0	0
*Longitarsus juncicola*	0	0.6	1	2	1	0	1	2	1	0	42	0	0	0
*Longitarsus minusculus*	6.8	9.0	8	0	0	1	9	0	0	0	9	0	0	0
*Longitarsus nigrocillus*	9.8	2.8	2	0	0	1	3	0	0	0	3	0	0	0
*Longitarsus obliteratus*	10.4	4.8	14	0	0	1	15	0	0	0	15	0	0	0
*Longitarsus ochroleucus*	2,8	15.2	29	0	0	1	29	0	1	0	29	0	0	0
*Longitarsus ordinatus*	0	8.8	152	15	0	0	152	15	0	0	129	0	0	0
*Longitarsus parvulus*	10.6	10.9	31	0	0	1	31	0	0	1	31	0	0	1
*Longitarsus pinguis*	11.8	13.7	5	0	0	1	5	0	0	1	5	0	0	1
*Longitarsus pratensis*	0	8.5	145	18	1	2	146	18	2	0	146	18	2	0
*Longitarsus refugiensis*	15.2	3.3	2	0	0	2	4	0	0	0	4	0	0	0
*Longitarsus reichei*	0	5.1	3	0	1	0	3	0	1	0	3	0	1	0
*Longitarsus salviae*	11.5	17.2	8	0	0	1	8	0	0	1	8	0	0	0
*Longitarsus scutellaris*	0	0.9	0	1	3	0	0	1	3	0	0	1	3	0
*Longitarsus succineus*	9.2	2.0	14	0	0	1	15	0	0	0	15	0	0	0

**Table 2:** Results of the TaxCI analyses. Taxonomic inconsistency for each species are reported as the number of: individuals of a given species not grouped as monophylum (tci), individuals of heterogeneous distance-based cluster (cl.het), individuals of a species found in more than one cluster (sp.split) and all individuals of a species in a homogeneous cluster with members in at least one other homogeneous cluster.

	Original Dataset				Optimal Threshold Dataset				Final Dataset			
Species	tci	cl.het	sp.split	other.homog	tci	cl.het	sp.split	other.homog	tci	cl.het	sp.split	other.homog
*L*. *aeneicollis*	0	8	8	0	0	8	0	0	0	8	0	0
*L*. *apicalis*	0	1	1	0	0	0	0	0	0	0	0	0
*L*. *atricillus*	67	67	67	0	67	67	67	0	67	67	0	0
*L*. *bedeli*	2	2	2	0	2	2	0	0	2	2	0	0
*L*. *curtus*	0	0	0	0	0	2	0	0	0	0	0	0
*L*. *erro*	48	48	0	48	48	45	45	0	48	45	45	0
*L*. *exsoletus*	0	89	0	89	0	0	0	0	0	0	0	0
*L*. *juncicola*	4	4	4	0	4	4	4	0	0	0	0	0
*L*. *kutscherae*	0	3	3	0	0	3	0	0	0	3	0	0
*L*. *lateripunctatus*	0	5	0	5	0	0	0	5	0	0	0	5
*L*. *lycopi*	0	15	0	15	0	0	0	15	0	0	0	15
*L*. *melanocephalus*	34	34	8	26	34	34	0	0	34	34	0	0
*L*. *minusculus*	9	9	0	9	9	0	0	9	9	0	0	9
*L*. *nasturtii*	0	0	0	0	0	3	0	0	0	3	0	0
*L*. *nigrocillus*	0	3	0	3	0	0	0	0	0	0	0	0
*L*. *nigrofasciatus*	26	26	0	26	26	0	0	26	26	0	0	26
*L*. *obliteratus*	0	15	0	15	0	0	0	0	0	0	0	0
*L*. *ochroleucus* s.str.	25	25	24	0	025	25	25	0	24	24	0	0
*L*. *ochroleucus lindbergi*	5	5	5	0	0	5	0	0	5	5	0	0
*L*. *ordinatus*	167	167	15	152	167	167	38	0	0	0	0	0
*L*. *parvulus*	0	32	0	32	0	0	0	32	0	0	0	32
*L*. *pellucidus*	0	22	0	22	0	0	0	0	0	0	0	0
*L*. *pinguis*	0	6	0	6	0	0	0	6	0	0	0	6
*L*. *pratensis*	166	166	151	15	166	166	166	0	166	166	0	0
*L*. *refugiensis*	0	4	0	4	0	0	0	0	0	0	0	0
*L*. *reichei*	4	4	1	0	4	4	4	0	4	4	0	0
*L*. *salviae*	9	9	0	9	9	9	0	9	0	0	0	0
*L*. *scutellaris*	4	4	4	0	4	4	4	0	4	4	0	0
*L*. *suturellus*	0	7	0	7	0	0	0	0	4	4	0	0
*L*. *tabidus*	0	25	0	25	0	0	0	0	0	0	0	0

### Optimal threshold dataset

**Fig 5:**
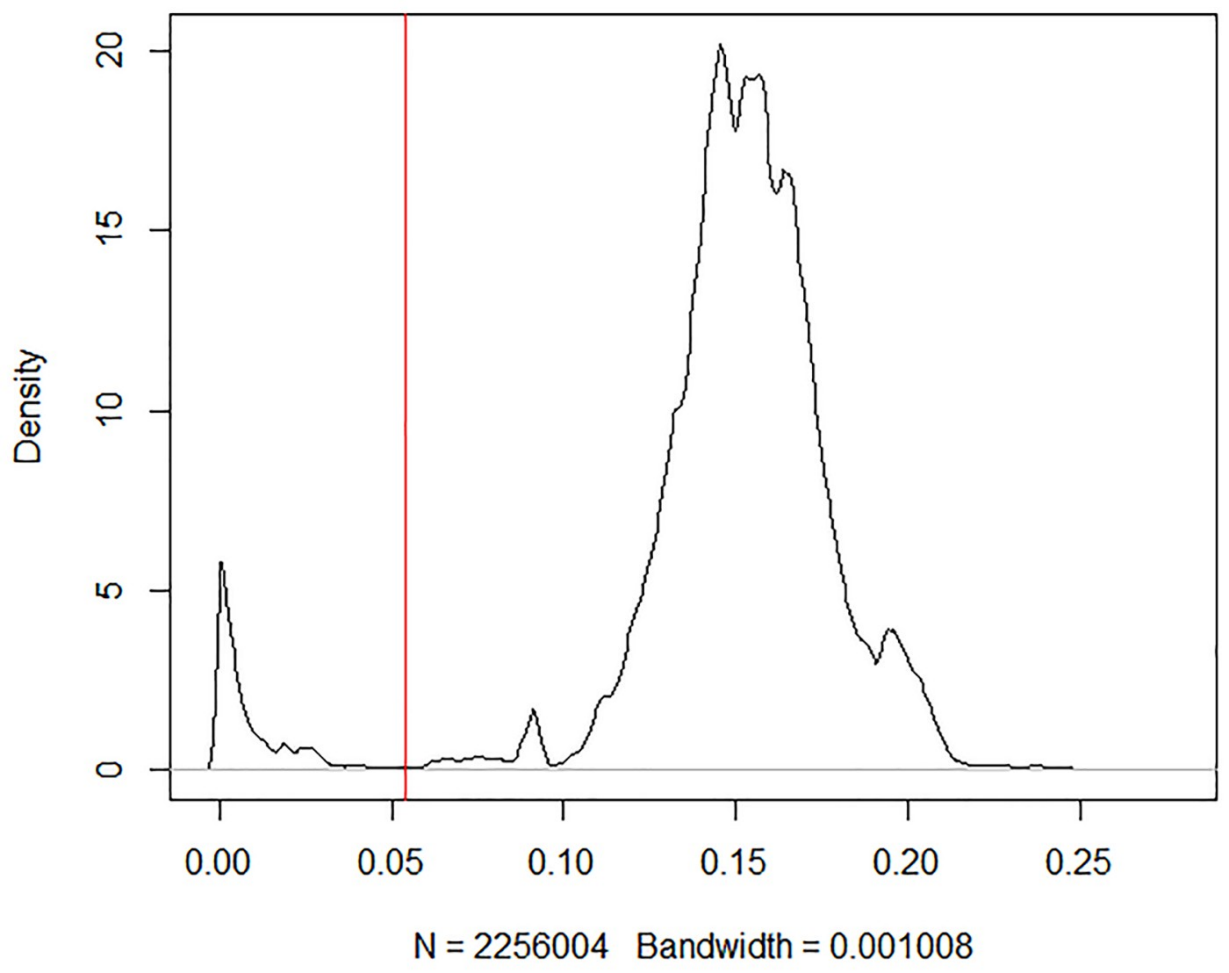
Results of the threshold optimisation analysis. The best threshold is identified as the dip in the density of genetic distances that indicates a transition between intra- and inter-specific distances. The optimised threshold is indicated by the red vertical line.

The optimal distance threshold for *Longitarsus* was estimated at 5.4% (Fig 5). Ten singleton sequences have been identified and removed [*L*. *apicalis* (Beck, 1817), *L*. *fallax* Weise, 1888, *L*. *fulgens* (Foudras, 1860), *L*. *linnaei* (Duftschmid, 1825), *L*. *nanus* (Foudras, 1860), *L*. *niger* (Koch, 1803), *L*. *nigripennis* Motschulsky, 1866, *L*. *rubellus* (Foudras, 1860), *L*. *saulicus* Gruev & Döberl, 2005 and *L*. *vilis* Wollaston, 1864]. Once the optimal threshold has been set and the singletons removed, the barcoding identification efficiency of *Longitarsus* evaluated through the best close match analysis, increased to 96% of correct identification (1442 out of 1492). Remaining sequences resulted in 36 ambiguous sequences, 11 incorrect sequences and a net decrease in the number of no id sequences (3 sequences) (Table 1). Consistent with the other analyses, following threshold optimization, TaxCI results show a decrease in heterospecific clusters, from 28 of the original dataset, to 16 cases (Table 2 and S7 Fig).

### Final dataset

**Fig 6:**
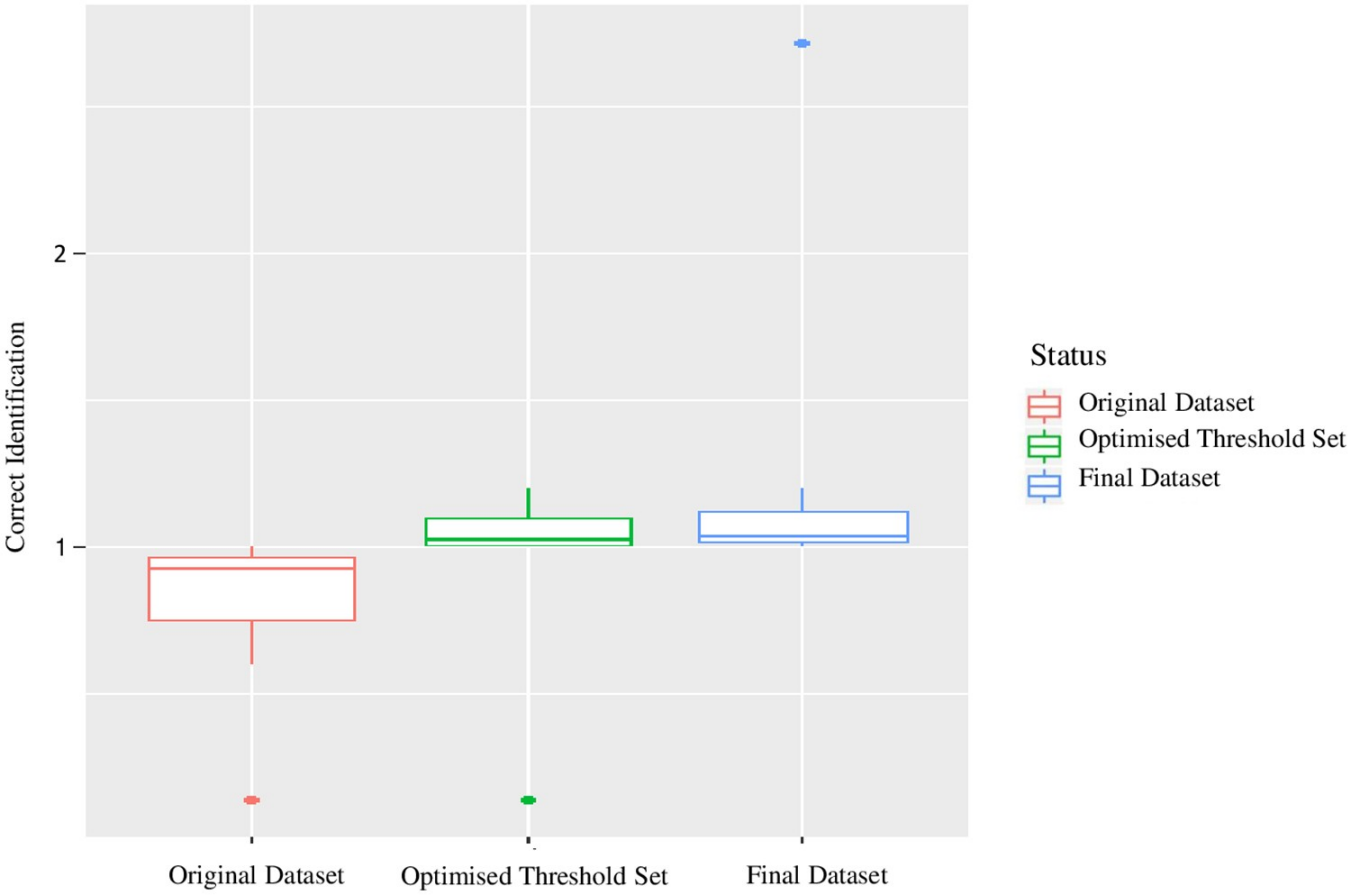
Results of the ANOVA analyses comparing the correct species identification ratio of the original dataset (red), the optimised threshold dataset (green), and the final dataset (blue).

As a last step, available voucher material relative to ambiguous and incorrect identification sequences was assessed by Maurizio Biondi to confirm or not the identification error. Thanks to this procedure we were able to identify at least 69 incorrect specimen identifications. Among these, based on morphological assessment of voucher materials we identified several *L*. *juncicola* (Foudras, 1860) that had been wrongly identified as *L*. *ordinatus* (Foudras, 1860). Three outlier sequences belonging to *L*. *atricillus*, *L*. *salviae* Gruev, 1975 and *L*. *ochroleucus* (Marsham, 1802) were removed from the final dataset because they failed taxonomic validation. These three sequences have a large genetic divergence relative to conspecific sequences (*L*. *atricillus*: 6.3%; *L*. *ochroleucus*: 15,2%; *L*. *salviae*: 17,2%). The sequences of *L*. *ochroleucus* and *L*. *salviae* do not cluster with conspecific sequences, but rather they form singletons (a single branch with no affinity to other species) suggesting ambiguous identification (*sensu* Meier et al., [27]); the sequence of *L*. *atricillus* clusters within an allospecific clade (within the *L*. *aeneicollis* clade), suggesting a misidentification (*sensu* Meier et al., [27]). On the other hand, all remaining sequences of *L*. *atricillus*, *L*. *salviae*, *L*. *ochroleucus* and *L*. *aeneicollis* form well-defined and homogeneous clusters and their identification was validated by our sequenced vouchers. After the taxonomic revision step, barcoding gap analysis, best close match analysis and TaxCI analysis were repeated to verify if the elimination and correction of erroneous sequences improved the identification accuracy of the barcoding tool. The difference between the *maximum intraspecific distance* and the *minimum interspecific distance* calculated for the remaining 1489 sequences indicates 361 cases in which the barcoding gap is not present (24% of sequences) (Fig 3). Overall, there was an average reduction in the absence of barcode gaps per species (24%). Furthermore, the barcoding identification efficiency, evaluated through the best close match analysis, resulted in 98.1% of correct identifications (1460 out of 1489). Most of the ambiguous and incorrect sequences (93%) belong to the *L*. *pratensis* group. The remaining cases regard two sequences of *L*. *bedeli* Uhagon, 1887; this species according to Baselga et al [65], at the mitochondrial DNA level is not differentiated from *L*. *atricillus* (Linnaeus, 1761) (Table 1). Also TaxCI analysis found an improvement in the taxonomic consistency of the final dataset, with 13 heterospecific clusters. The *L*. *pratensis* group represented 48% of the sequences belonging to non-monophyletic species. Seven species are identified as heterogeneous distance-based clusters by TaxCI: *L*. *aeneicollis*, *L*. *atricillus*, *L*. *bedeli*, *L*. *erro*, *L*. *kutscherae* (Rye, 1872), *L*. *melanocephalus* (Geer, 1775) and *L*. *nasturtii* (Fabricius, 1793) (Table 2 and S8 Fig). The significant increase of sequences per species identified as correct after taxonomic revision was confirmed by ANOVA results. While the increase of correct identifications from the original dataset to the dataset with the set threshold (*F*-value = 2.138, *p*-value = 0.159) was not significant, the increase of correct identifications from the original dataset to the final dataset was statistically significant (*F*-value = 3.38, *p*-value<0.05) (Fig 6).

## Discussion

DNA barcoding is a molecular tool for species identification, and like for any tool, it is essential to know its potential as much as its limits. In this study we focused on identifying errors that affect the accuracy of DNA barcoding, distinguishing between tool *extrinsic errors*, i.e. those relative to the quality of the reference dataset, and *intrinsic errors*, i.e. those due to all those biological processes that generate a mismatch between mtDNA groups and species boundaries, thus making the barcoding tool unreliable in identifying specimens to the species level. While *intrinsic errors* to be solved require an integrative taxonomic and evolutionary study approach, which goes beyond the idea of barcoding as identification tool, *extrinsic errors* are due to human mistakes and can be corrected much more easily. Thus, in a reference dataset free of *extrinsic errors* it would be easy to spot species identification inconsistencies that require further taxonomic research.

In this study we identified the *extrinsic errors* occurring in the available *cox1* sequence dataset of the taxonomically complex genus *Longitarsus*. Barcoding gap analyses of this dataset showed several instances of overlap between intra- and interspecific genetic distance within this genus. The use of an *ad hoc* distance threshold, optimised for this dataset, resulted in an improvement of the quality of identification in agreement with previous studies [36, 66, 67]. However, the use of an optimal threshold did not significantly reduce the taxonomic uncertainty of the barcoding tool that was mostly associated to the *extrinsic errors* occurring in the reference datasets. These kinds of errors were readily identified using bioinformatic pipelines such as TaxCI, amended through a taxonomic revision carried out by the group specialist, and implemented in the reference database. The correct assignment of these misidentified sequences significantly increased the barcoding identification accuracy up to 98.1%. This identification rate is comparable to that found in other studies on Alticini [68] or Chrysomelidae [69], showing the utility of DNA barcoding as molecular identification tool of taxonomically diverse groups.

Once the *extrinsic errors* were removed, we have been able to identify those areas of the dataset affected by *intrinsic errors*. Taxonomic uncertainty within the *L*. *pratensis* species group account for 93% of such *intrinsic errors*. This group is represented in the dataset by *L*. *pratensis* (Panzer, 1794), *L*. *scutellaris* (Rey, 1874) and *L*. *reichei* (Allard, 1860). All the analyses showed that specimens assigned to these species are genetically undifferentiated one each other. The high morphological similarity of these species and their sympatric distribution makes them extremely difficult to identify [70, 71]. Species boundaries within this group are not well defined due to the lack of a comprehensive and integrative taxonomic assessment combining morphological and molecular approaches. The remaining *intrinsic errors* identified in this study regard the species pair *L*. *atricillus* and *L*. *bedeli* and has been already discussed in a previous work [65]. These two species show morphological differences on elytral coloration and female genitalia, with no morphological intermediates, but mtDNA does not detect any distinguishable phylogenetic structure that allows to separate these two species [65]. Also in this case an integrative approach will be required to reach a firm taxonomic conclusion; the use of multiple nuclear loci would allow disentangling lineage sorting or mitochondrial introgression as the processes responsible for the observed mitochondrial pattern.

On the other hand, we identified different species that, despite being monophyletic in the TaxCI analyses, are characterized by (i) a high intraspecific divergence such as *L*. *pinguis* Weise, 1888, *L*. *parvulus* (Paykull, 1799), *L*. *lateripunctatus* Rosenhauer, 1856 and *L*. *lycopi* (Foudras, 1860); or by (ii) a low interspecific divergence such as between, *L*. *nasturtii* and *L*. *erro*, *L*. *atricillus* and *L*. *aeneicollis*. (i) In *L*. *pinguis* high genetic distance was observed between specimens collected in northern Italy (Lombardia region) and specimens from central Italy. As for *L*. *parvulus*, high genetic distance is found between the unique Greek specimen and specimens from central-western Europe. In *L*. *lateripunctatus* a high genetic distance is observed between specimens from the opposite sides of the Apennine mountains in central Italy. For these three species the high genetic distance seems to be associated to a geographic structure. Instead, genetic variation within *L*. *lycopi*, does not seem to have geographical structure. (ii) The species within the two pairs *L*. *nasturtii*/*L*. *erro* and *L*. *atricillus*/*L*. *aeneicollis* form two reciprocally monophyletic sister clades with limited genetic distance, suggesting a recent divergence. This analysis also confirms the monophyly of *L*. *ochroleucus lindbergi* (Madar, 1963) within the *Longitarsus ochroleucus* clade, supporting the validity of this subspecies that is endemic to Madeira (Portugal).

Moreover, TaxCI results identified some non-monophyletic species that deserve taxonomic attention. Sequences belonging to *L*. *kutscherae* (Rye, 1872) are nested within the clade of *L*. *melanocephalus*. These two species are morphologically very similar and have a sympatric distribution [70]. However, due to the absence of metadata associated to these sequences, we were unable to verify whether this phylogenetic pattern is due to an incorrect specimen identification, to a poorly established taxonomy of these species, or because of any biological processes causing *intrinsic* type errors. *L*. *minusculus* (Foudras, 1860), *L*. *nigrofasciatus* (Goeze, 1777) and *L*. *erro* are polyphyletic groups. All these species present a large distribution and the reasons for the absence of monophyly can be manifold and should be explored.

The importance of a dataset free of *extrinsic error* for the accuracy of the DNA barcoding tool cannot be overstated. Depositing only high-quality sequences correctly annotated with correct species names in public repositories would be the “golden standard” and is crucial for keeping the global barcode library functional and reliable [7, 8, 40]. The high number of taxonomists required to avoid any error in morphological identification of species should not be an impediment of large-scale DNA barcoding campaigns [72], especially in a period of risk for biodiversity that calls for a rapid assessment of species identification. On the other hand, this should not coincide with the risk of large but low-quality data production, thus it is fundamental maintaining a standard that allows a posteriori verification of identifications via morphological analysis [21, 73–75]. In this regard we proved the efficacy of a non-invasive DNA extraction protocol that allows successful amplification of the barcoding gene fragment in flea beetle specimens as small as 1.5 mm. Using this non-invasive extraction methods has allowed us to maintain a reference voucher sample for future taxonomic assessments.

In conclusion, results of this study show that while taxonomic inconsistencies in reference sequence databases greatly affect the DNA barcoding accuracy, they can be readily identified using bioinformatic pipelines, and resolved through a posteriori re-assessment by an expert taxonomist based on available metadata, vouchers, or newly generated sequences [75–77]. Once again, this study underlines the key role of taxonomists in any step of the DNA barcoding pipeline, from the initial association of DNA sequences with morphologically identified species to the *a posteriori* revision of the inconsistencies identified in the reference database. Furthermore, such step of taxonomic revision on existing data allows identifying hot research areas for *Longitarsus* taxonomy, further corroborating the intimate link between the accuracy of the DNA barcoding tool and taxonomic knowledge.

## Supporting information

S1 Table List of cox1 longitarsus sequences retrieved from GenBank and BOLD and used in this study. (PDF)

S2 Table List of specimens sequenced in this study. For each specimen are reported voucher info, place and date of collection, coordinates, collectors, BOLD and GenBank accession numbers. All specimens have been identified by Maurizio Biondi (University of L’Aquila).
(PDF)

S1 Fig Photographs of habitus and aedeagus or spermatheca of (a) *Longitarsus albineus* ♂; (b) *L*. *exsoletus* ♂; (c) *L*. *juncicola* ♂; (d) *L*. *ochroleucus lindbergi* ♂; (e) *L*. *laureolae* ♂; (f) *L*. *luridus* ♂. Scale bar 0.5 mm. (PDF)

S2 Fig Photographs of habitus and aedeagus or spermatheca of (a) *Longitarsus ordinatus* ♂; (b) *L*. *nigrofasciatus* ♂; (c) *L*. *melanocephalus* ♂; (d) *L*. *pinguis* ♂; (e) *L*. *parvulus* ♂; (f) *L*. *rectilineatus* ♂. Scale bar 0.5 mm. (PDF)

S3 Fig Photogaphs of habitus and aedeagus or spermatheca of (a) *Longitarsus salviae* ♂; (b) *L*. *strigicollis* ♂; (c) *L*. *springeri* ♂; (d) *L*. *succineus* ♂; (e) *L*. *tabidus* ♂; (f) *L*. *zangherii* ♂. Scale bar 0.5 mm. (PDF)

S4 Fig Linear regression models showing (a) the relationships between yield of DNA extraction and time of permanence in alcohol of the specimens, *tp*; and (b) the body size of the specimens, *bs*. For each species two specimens were selected and DNA extracted either with the invasive method, IM, or with the non-invasive method, NIM (*bs*-IM: *R2* = 0.1053, *p*-value = 0.1407; *bs*-NIM: *R2* = 0.0155, *p*-value = 0.581; *tp*-IM: *R2* = 0.07261, *p*-value = 0.2252; *tp*-NIM: *R2* = 0.1191, *p*-value = 0.1156).
(PDF)

S5 Fig Comparison of DNA extraction yield, amplification and sequencing success between the invasive DNA extraction method, IM, and the non-invasive DNA extraction method, NIM. (a) ANOVA results comparing the final amount of the DNA extracted with the two methods. (b) Amplification success and (c) Sanger sequencing success of the *cox1* gene fragment using DNA templates obtained with the two DNA extraction methods. Bands obtained with DNA templates extracted with the NIM are marked on the electrophoretic gel. Sequencing success rate for PCR products obtained using DNA templates extracted with the two methods: IM, 90% good, 10% poor and 0% failed; NIM, 91% good, 6% poor and 2% failed.
(PDF)

S6 Fig Tree output from TaxCI analysis on the original dataset. (PDF)

S7 Fig Tree output from TaxCI analysis on the optimised threshold dataset. (PDF)

S8 Fig Tree output from TaxCI analysis on the final dataset. (PDF)

S1 Raw images (PDF)
