# Supplementary material for: Sharpening the DNA barcoding tool through a posteriori taxonomic validation: The case of Longitarsus flea beetles (Coleoptera: Chrysomelidae)
Source: PLoS One. 2020 May 21;15(5):e0233573. doi: 10.1371/journal.pone.0233573 (PMC7241800; doi:10.1371/journal.pone.0233573)
Supplement: S1 Table — (PDF) [file pone.0233573.s001.pdf]

Supplementary Table S1. List of *cox1 Longitarsus* sequences retrieved from GenBank and BOLD and used in this study.

N	Species	BOLD ID	GenBank ID
1	*Longitarsus absynthii*	GCOL2597-16	KU911390
2	*Longitarsus absynthii*	GCOL2596-16	KU915191
3	*Longitarsus absynthii*	GCOL2595-16	KU907959
4	*Longitarsus aeneicollis*	MEDLB342-12	MH323184
5	*Longitarsus aeneicollis*	MEDLB345-12	MH323186
6	*Longitarsus aeneicollis*	MEDLB337-12	MH323185
7	*Longitarsus aeneicollis*		MK893902
8	*Longitarsus aeneus*	GBCCH9976-19	KF652626
9	*Longitarsus aeneus*	GBCCH9988-19	KF652304
10	*Longitarsus aeneus*	GBCCH9989-19	KF652455
11	*Longitarsus aeneus*	GBCCH9991-19	KF652465
12	*Longitarsus aeneus*	GBCCH9993-19	KF652469
13	*Longitarsus aeneus*	GBCCH9974-19	KF652606
14	*Longitarsus aeneus*	GBCCH9975-19	KF652625
15	*Longitarsus aeneus*	GBCCH9978-19	KF652671
16	*Longitarsus aeneus*	GBCCH9983-19	KF652910
17	*Longitarsus aeneus*	GBCCH9985-19	KF652919
18	*Longitarsus aeneus*	GBCCH9996-19	KF652482
19	*Longitarsus aeneus*	GBCCH9977-19	KF652627
20	*Longitarsus aeneus*	GBCCH10000-19	KF652544
21	*Longitarsus aeneus*	GBCCH9999-19	KF652517
22	*Longitarsus aeneus*	GBCCH9984-19	KF652911
23	*Longitarsus aeneus*	GBCCH10009-19	KF653934
24	*Longitarsus aeneus*	GBCCH9982-19	KF652715
25	*Longitarsus aeneus*	GBCCH9980-19	KF652704
26	*Longitarsus aeneus*	GBCCH9987-19	KF652921
27	*Longitarsus aeneus*	GBCCH9998-19	KF652516
28	*Longitarsus aeneus*	GBCCH10011-19	KF654077
29	*Longitarsus aeneus*	GBCCH9973-19	KF652605
30	*Longitarsus aeneus*	GBCCH9986-19	KF652920
31	*Longitarsus aeneus*	GBCCH10005-19	KF653635
32	*Longitarsus aeneus*	GBCCH10010-19	KF653987
33	*Longitarsus aeneus*	GBCCH10012-19	KF654085
34	*Longitarsus aeneus*	GBCCH9972-19	KF652747
35	*Longitarsus aeneus*	GBCCH9990-19	KF652464
36	*Longitarsus aeneus*	GBCCH9995-19	KF652480
37	*Longitarsus aeneus*	GBCCH10014-19	KX943357
38	*Longitarsus aeneus*	GBCCH10004-19	KF653389
39	*Longitarsus aeneus*	GBCCH10007-19	KF653637
40	*Longitarsus aeneus*	GBCCH9997-19	KF652490
41	*Longitarsus aeneus*	GBCCH10003-19	KF653385
42	*Longitarsus aeneus*	GBCCH10002-19	KF652549
43	*Longitarsus aeneus*	GBCCH10006-19	KF653636
44	*Longitarsus aeneus*	GBCCH9992-19	KF652468
45	*Longitarsus aeneus*	GBCCH9994-19	KF652478
46	*Longitarsus aeneus*	GBCCH10001-19	KF652545

47	*Longitarsus aeneus*	GBCCH10008-19	KF653653
48	*Longitarsus aeneus*	GBCCH10013-19	KP763017
49	*Longitarsus aeneus*	GBCCH9979-19	KF652703
50	*Longitarsus aeneus*	GBCCH9981-19	KF652713
51	*Longitarsus anchusae*	GBCCH10015-19	HQ165504
52	*Longitarsus anchusae*	GCOL2650-16	KU906769
53	*Longitarsus anchusae*	GBCOL441-12	KM448392
54	*Longitarsus anchusae*	GBCOU354-13	KM444377
55	*Longitarsus anchusae*	FBCOA528-10	HQ953413
56	*Longitarsus anchusae*	FBCOG940-12	KM445196
57	*Longitarsus anchusae*	FBCOK732-13	KM448881
58	*Longitarsus anchusae*	GBCOC415-12	KM451773
59	*Longitarsus anchusae*	GCOL12698-16	KU918470
60	*Longitarsus anchusae*	GBCOC465-12	KM441554
61	*Longitarsus anchusae*	GBCOD189-13	KM448991
62	*Longitarsus anchusae*	FBCOP371-13	KM450548
63	*Longitarsus anchusae*	GCOL2629-16	KU909318
64	*Longitarsus anchusae*	GCOL2652-16	KU919252
65	*Longitarsus anchusae*	GBCOC679-12	KM446101
66	*Longitarsus apicalis*	MEDLB184-10	JF890820
67	*Longitarsus atricillus*	GBCCH10025-19	KF652311
68	*Longitarsus atricillus*	GBCCH10042-19	KF653628
69	*Longitarsus atricillus*	GBCCH10063-19	KX943363
70	*Longitarsus atricillus*	GBCCH1732-14	KF134575
71	*Longitarsus atricillus*	GBCCH10021-19	KF652305
72	*Longitarsus atricillus*	GBCCH1713-14	KF134548
73	*Longitarsus atricillus*	GCOL2525-16	KU913100
74	*Longitarsus atricillus*	GBCCH10020-19	KF652300
75	*Longitarsus atricillus*	GBCCH10054-19	KF654487
76	*Longitarsus atricillus*	GBCCH1641-14	KF134570
77	*Longitarsus atricillus*	GBCCH10017-19	KF652303
78	*Longitarsus atricillus*	GBCCH10022-19	KF652306
79	*Longitarsus atricillus*	GBCCH10026-19	KF652315
80	*Longitarsus atricillus*	GBCCH10044-19	KF653631
81	*Longitarsus atricillus*	GBCCH10051-19	KF654236
82	*Longitarsus atricillus*	GBCCH1686-14	KF134566
83	*Longitarsus atricillus*	GBCCH1765-14	KF134574
84	*Longitarsus atricillus*	GBCCH10029-19	KF652466
85	*Longitarsus atricillus*	GBCCH10032-19	KF652489
86	*Longitarsus atricillus*	GBCCH10062-19	KP763034
87	*Longitarsus atricillus*	GBCCH10024-19	KF652308
88	*Longitarsus atricillus*	GBCCH10039-19	KF653618
89	*Longitarsus atricillus*	GBCCH10050-19	KF654235
90	*Longitarsus atricillus*	GBCCH10055-19	KF654664
91	*Longitarsus atricillus*	GBCCH10057-19	KF655011
92	*Longitarsus atricillus*	GBCCH10061-19	KF656131
93	*Longitarsus atricillus*	GBCCH1635-14	KF134581
94	*Longitarsus atricillus*	GBCCH10027-19	KF652385
95	*Longitarsus atricillus*	GBCCH10049-19	KF654232
96	*Longitarsus atricillus*	GBCCH10053-19	KF654486
97	*Longitarsus atricillus*	GCOL2440-16	KU915650

98	*Longitarsus atricillus*	GBCCH10028-19	KF652410
99	*Longitarsus atricillus*	GBCCH10056-19	KF655009
100	*Longitarsus atricillus*	GBCCH1640-14	KF134569
101	*Longitarsus atricillus*	GBCCH1712-14	KF134547
102	*Longitarsus atricillus*	GBCCH10038-19	KF653101
103	*Longitarsus atricillus*	GBCCH10043-19	KF653630
104	*Longitarsus atricillus*	GBCCH10046-19	KF653876
105	*Longitarsus atricillus*	GBCCH10023-19	KF652307
106	*Longitarsus atricillus*	GBCCH10031-19	KF652488
107	*Longitarsus atricillus*	GBCCH10034-19	KF652805
108	*Longitarsus atricillus*	GBCCH10048-19	KF653900
109	*Longitarsus atricillus*	GCOL2592-16	KU911269
110	*Longitarsus atricillus*	GBCCH10036-19	KF653024
111	*Longitarsus atricillus*	GBCCH10041-19	KF653626
112	*Longitarsus atricillus*	GBCCH10060-19	KF655357
113	*Longitarsus atricillus*	GBCCH10030-19	KF652479
114	*Longitarsus atricillus*	GBCCH10033-19	KF652787
115	*Longitarsus atricillus*	GBCCH10040-19	KF653622
116	*Longitarsus atricillus*	GBCCH10016-19	KF653025
117	*Longitarsus atricillus*	GBCCH10018-19	KF652312
118	*Longitarsus atricillus*	GBCCH10035-19	KF653023
119	*Longitarsus atricillus*	GBCCH1658-14	KF134554
120	*Longitarsus atricillus*	GBCCH10047-19	KF653877
121	*Longitarsus atricillus*	GBCCH10052-19	KF654447
122	*Longitarsus atricillus*	GBCCH1710-14	KF134545
123	*Longitarsus atricillus*	GBCCH10059-19	KF655350
124	*Longitarsus atricillus*	FBCOA720-10	HQ953558
125	*Longitarsus atricillus*	GBCCH10037-19	KF653026
126	*Longitarsus atricillus*	GBCCH10045-19	KF653670
127	*Longitarsus atricillus*	GBCCH10058-19	KF655024
128	*Longitarsus atricillus*	GBCCH1711-14	KF134546
129	*Longitarsus atricillus*	GBCCH1715-14	KF134560
130	*Longitarsus atricillus*	GBCCH1716-14	KF134561
131	*Longitarsus atricillus*	GBCCH10019-19	KF652624
132	*Longitarsus atricillus*	GBCOC705-12	KM450237
133	*Longitarsus atricillus*	GCOL2353-16	KU906420
134	*Longitarsus atricillus*	GBCCH1714-14	KF134549
135	*Longitarsus ballotae*	GBCCH10064-19	KF653462
136	*Longitarsus ballotae*	FBCOK031-13	KM441283
137	*Longitarsus ballotae*	GBCCH10068-19	MF979912
138	*Longitarsus ballotae*	GCOL6836-16	KU913658
139	*Longitarsus ballotae*	GBCOL451-12	KM447081
140	*Longitarsus ballotae*	GBCOU1923-13	KM446857
141	*Longitarsus ballotae*	GCOL2601-16	KU908505
142	*Longitarsus ballotae*	GBCOL527-12	KM450245
143	*Longitarsus ballotae*	GCOL2574-16	KU916512
144	*Longitarsus ballotae*	GBCCH10067-19	HQ164607
145	*Longitarsus ballotae*	GBCOL518-12	KM441312
146	*Longitarsus ballotae*	FBCOK030-13	KM439764
147	*Longitarsus ballotae*	GBCCH10065-19	KF653463
148	*Longitarsus ballotae*	GBMIN49043-17	KC185781

149	*Longitarsus ballotae*	GBCOL450-12	KM442044
150	*Longitarsus ballotae*	GCOL2658-16	KU915340
151	*Longitarsus ballotae*	GCOL6835-16	KU915077
152	*Longitarsus ballotae*	GBCCH10066-19	KC185681
153	*Longitarsus ballotae*	GBCOU1924-13	KM442685
154	*Longitarsus bedeli*	GBCCH1763-14	KF134559
155	*Longitarsus bedeli*	GBCCH10069-19	KP763019
156	*Longitarsus bedeli*	GBCCH1762-14	KF134558
157	*Longitarsus brisouti*	GBCOC414-12	KM439696
158	*Longitarsus brisouti*	FBCOG938-12	KM443993
159	*Longitarsus brisouti*	GBCOC413-12	KM440067
160	*Longitarsus brunneus*	COLFE245-12	KJ964332
161	*Longitarsus brunneus*	COLFF801-13	KJ962114
162	*Longitarsus brunneus*	GCOL2431-16	KU916779
163	*Longitarsus brunneus*	GBCCH10070-19	HQ165401
164	*Longitarsus brunneus*	GCOL2712-16	KU909519
165	*Longitarsus brunneus*	FBCOD1172-11	KM451771
166	*Longitarsus brunneus*	GCOL2667-16	KU906469
167	*Longitarsus brunneus*	FBCOK053-13	KM452511
168	*Longitarsus brunneus*	COLFE244-12	KJ962949
169	*Longitarsus brunneus*	COLFF802-13	KJ962718
170	*Longitarsus brunneus*	GCOL2432-16	KU910782
171	*Longitarsus candidulus*	GBCCH10071-19	KF654907
172	*Longitarsus candidulus*	GBCCH10076-19	KF654954
173	*Longitarsus candidulus*	GBCCH10078-19	KX943430
174	*Longitarsus candidulus*	GBCCH10072-19	KF654908
175	*Longitarsus candidulus*	GBCCH10074-19	KF654945
176	*Longitarsus candidulus*	GBCCH10077-19	KF654955
177	*Longitarsus candidulus*	GBCCH10073-19	KF654944
178	*Longitarsus candidulus*	GBCCH10075-19	KF654946
179	*Longitarsus celticus*	GCOL2728-16	KU913183
180	*Longitarsus celticus*	FBCOB336-10	HQ953958
181	*Longitarsus celticus*	GCOL2726-16	KU908777
182	*Longitarsus celticus*	GCOL2727-16	KU916791
183	*Longitarsus celticus*	FBCOB335-10	HQ953957
184	*Longitarsus cerinthes*	GBCCH10091-19	KF652281
185	*Longitarsus cerinthes*	GBCCH10111-19	KF652680
186	*Longitarsus cerinthes*	GBCCH10155-19	KF653659
187	*Longitarsus cerinthes*	GBCCH10168-19	KF654270
188	*Longitarsus cerinthes*	GBCCH10187-19	KF654916
189	*Longitarsus cerinthes*	GBCCH10188-19	KP762976
190	*Longitarsus cerinthes*	GBCCH10123-19	KF653608
191	*Longitarsus cerinthes*	GBCCH10100-19	KF652384
192	*Longitarsus cerinthes*	GBCCH10113-19	KF652909
193	*Longitarsus cerinthes*	GBCCH10156-19	KF653679
194	*Longitarsus cerinthes*	GBCCH10084-19	KF652553
195	*Longitarsus cerinthes*	GBCCH10092-19	KF652284
196	*Longitarsus cerinthes*	GBCCH10110-19	KF652679
197	*Longitarsus cerinthes*	GBCCH10118-19	KF653118
198	*Longitarsus cerinthes*	GBCCH10166-19	KF654234
199	*Longitarsus cerinthes*	GBCCH10177-19	KF654364

200	*Longitarsus cerinthes*	GBCCH10178-19	KF654372
201	*Longitarsus cerinthes*	GBCCH10096-19	KF652342
202	*Longitarsus cerinthes*	GBCCH10099-19	KF652369
203	*Longitarsus cerinthes*	GBCCH10104-19	KF652513
204	*Longitarsus cerinthes*	GBCCH10120-19	KF653120
205	*Longitarsus cerinthes*	GBCCH10124-19	KF653609
206	*Longitarsus cerinthes*	GBCCH10127-19	KF653612
207	*Longitarsus cerinthes*	GBCCH10082-19	KF654315
208	*Longitarsus cerinthes*	GBCCH10088-19	KF652250
209	*Longitarsus cerinthes*	GBCCH10112-19	KF652681
210	*Longitarsus cerinthes*	GBCCH10115-19	KF653103
211	*Longitarsus cerinthes*	GBCCH10157-19	KF653767
212	*Longitarsus cerinthes*	GBCCH10159-19	KF653769
213	*Longitarsus cerinthes*	GBCCH10164-19	KF653986
214	*Longitarsus cerinthes*	GBCCH10083-19	KF654332
215	*Longitarsus cerinthes*	GBCCH10093-19	KF652325
216	*Longitarsus cerinthes*	GBCCH10095-19	KF652341
217	*Longitarsus cerinthes*	GBCCH10103-19	KF652512
218	*Longitarsus cerinthes*	GBCCH10121-19	KF653121
219	*Longitarsus cerinthes*	GBCCH10125-19	KF653610
220	*Longitarsus cerinthes*	GBCCH10129-19	KF653614
221	*Longitarsus cerinthes*	GBCCH10145-19	KF653643
222	*Longitarsus cerinthes*	GBCCH10185-19	KF654914
223	*Longitarsus cerinthes*	GBCCH10089-19	KF652254
224	*Longitarsus cerinthes*	GBCCH10102-19	KF652506
225	*Longitarsus cerinthes*	GBCCH10116-19	KF653104
226	*Longitarsus cerinthes*	GBCCH10131-19	KF653616
227	*Longitarsus cerinthes*	GBCCH10132-19	KF653617
228	*Longitarsus cerinthes*	GBCCH10186-19	KF654915
229	*Longitarsus cerinthes*	GBCCH10122-19	KF653469
230	*Longitarsus cerinthes*	GBCCH10136-19	KF653624
231	*Longitarsus cerinthes*	GBCCH10147-19	KF653648
232	*Longitarsus cerinthes*	GBCCH10151-19	KF653654
233	*Longitarsus cerinthes*	GBCCH10161-19	KF653931
234	*Longitarsus cerinthes*	GBCCH10094-19	KF652326
235	*Longitarsus cerinthes*	GBCCH10106-19	KF652556
236	*Longitarsus cerinthes*	GBCCH10119-19	KF653119
237	*Longitarsus cerinthes*	GBCCH10133-19	KF653619
238	*Longitarsus cerinthes*	GBCCH10144-19	KF653642
239	*Longitarsus cerinthes*	GBCCH10146-19	KF653644
240	*Longitarsus cerinthes*	GBCCH10165-19	KF654233
241	*Longitarsus cerinthes*	GBCCH10173-19	KF654331
242	*Longitarsus cerinthes*	GBCCH10182-19	KF654383
243	*Longitarsus cerinthes*	GBCCH10080-19	KF653660
244	*Longitarsus cerinthes*	GBCCH10085-19	KF652657
245	*Longitarsus cerinthes*	GBCCH10135-19	KF653623
246	*Longitarsus cerinthes*	GBCCH10138-19	KF653627
247	*Longitarsus cerinthes*	GBCCH10081-19	KF652555
248	*Longitarsus cerinthes*	GBCCH10152-19	KF653655
249	*Longitarsus cerinthes*	GBCCH10153-19	KF653657
250	*Longitarsus cerinthes*	GBCCH10098-19	KF652358

251	*Longitarsus cerinthes*	GBCCH10140-19	KF653638
252	*Longitarsus cerinthes*	GBCCH10149-19	KF653650
253	*Longitarsus cerinthes*	GBCCH10158-19	KF653768
254	*Longitarsus cerinthes*	GBCCH10172-19	KF654330
255	*Longitarsus cerinthes*	GBCCH10189-19	KX943478
256	*Longitarsus cerinthes*	GBCCH10139-19	KF653629
257	*Longitarsus cerinthes*	GBCCH10171-19	KF654316
258	*Longitarsus cerinthes*	GBCCH10176-19	KF654363
259	*Longitarsus cerinthes*	GBCCH10181-19	KF654382
260	*Longitarsus cerinthes*	GBCCH10087-19	KF652669
261	*Longitarsus cerinthes*	GBCCH10117-19	KF653105
262	*Longitarsus cerinthes*	GBCCH10180-19	KF654381
263	*Longitarsus cerinthes*	GBCCH10109-19	KF652678
264	*Longitarsus cerinthes*	GBCCH10142-19	KF653640
265	*Longitarsus cerinthes*	GBCCH10167-19	KF654245
266	*Longitarsus cerinthes*	GBCCH10169-19	KF654286
267	*Longitarsus cerinthes*	GBCCH10184-19	KF654913
268	*Longitarsus cerinthes*	GBCCH10108-19	KF652558
269	*Longitarsus cerinthes*	GBCCH10163-19	KF653933
270	*Longitarsus cerinthes*	GBCCH10170-19	KF654296
271	*Longitarsus cerinthes*	GBCCH10175-19	KF654362
272	*Longitarsus cerinthes*	GBCCH10183-19	KF654912
273	*Longitarsus cerinthes*	GBCCH10097-19	KF652357
274	*Longitarsus cerinthes*	GBCCH10128-19	KF653613
275	*Longitarsus cerinthes*	GBCCH10148-19	KF653649
276	*Longitarsus cerinthes*	GBCCH10160-19	KF653930
277	*Longitarsus cerinthes*	GBCCH10086-19	KF652667
278	*Longitarsus cerinthes*	GBCCH10114-19	KF653102
279	*Longitarsus cerinthes*	GBCCH10090-19	KF652262
280	*Longitarsus cerinthes*	GBCCH10101-19	KF652500
281	*Longitarsus cerinthes*	GBCCH10107-19	KF652557
282	*Longitarsus cerinthes*	GBCCH10126-19	KF653611
283	*Longitarsus cerinthes*	GBCCH10137-19	KF653625
284	*Longitarsus cerinthes*	GBCCH10150-19	KF653651
285	*Longitarsus cerinthes*	GBCCH10162-19	KF653932
286	*Longitarsus cerinthes*	GBCCH10174-19	KF654361
287	*Longitarsus cerinthes*	GBCCH10079-19	KF653620
288	*Longitarsus cerinthes*	GBCCH10130-19	KF653615
289	*Longitarsus cerinthes*	GBCCH10141-19	KF653639
290	*Longitarsus cerinthes*	GBCCH10179-19	KF654373
291	*Longitarsus cerinthes*	GBCCH10105-19	KF652552
292	*Longitarsus cerinthes*	GBCCH10134-19	KF653621
293	*Longitarsus cerinthes*	GBCCH10143-19	KF653641
294	*Longitarsus cerinthes*	GBCCH10154-19	KF653658
295	*Longitarsus curtus*	GBCCH10191-19	KX943501
296	*Longitarsus curtus*	GBCCH10190-19	KF656258
297	*Longitarsus dorsalis*	GMGMF1070-14	
298	*Longitarsus dorsalis*	GBCCH10199-19	KF652644
299	*Longitarsus dorsalis*	GBCCH10202-19	KF652443
300	*Longitarsus dorsalis*	GBCCH10225-19	KF653320
301	*Longitarsus dorsalis*	GCOL1329-16	KU909761

302	*Longitarsus dorsalis*	GBCCH10205-19	KF652450
303	*Longitarsus dorsalis*	GBCCH10206-19	KF652451
304	*Longitarsus dorsalis*	GBCCH10214-19	KF652729
305	*Longitarsus dorsalis*	GBCCH10217-19	KF652766
306	*Longitarsus dorsalis*	GBCCH10229-19	KF653353
307	*Longitarsus dorsalis*	GBCCH10243-19	KF653405
308	*Longitarsus dorsalis*	GBCCH1660-14	KF134555
309	*Longitarsus dorsalis*	GBCCH1661-14	KF134556
310	*Longitarsus dorsalis*	GBCCH1676-14	KF134568
311	*Longitarsus dorsalis*	GCOL2625-16	KU918988
312	*Longitarsus dorsalis*	GBCCH10222-19	KF653287
313	*Longitarsus dorsalis*	GBCCH10270-19	KF654098
314	*Longitarsus dorsalis*	GBCCH10215-19	KF652735
315	*Longitarsus dorsalis*	GBCCH10216-19	KF652736
316	*Longitarsus dorsalis*	GBCCH10238-19	KF653400
317	*Longitarsus dorsalis*	GBCCH10267-19	KF653746
318	*Longitarsus dorsalis*	GCOL7046-16	KU917016
319	*Longitarsus dorsalis*	GBCCH10198-19	KF652640
320	*Longitarsus dorsalis*	GBCCH10213-19	KF652728
321	*Longitarsus dorsalis*	GBCCH10242-19	KF653404
322	*Longitarsus dorsalis*	GBCCH10265-19	KF653724
323	*Longitarsus dorsalis*	GBCCH1717-14	KF134550
324	*Longitarsus dorsalis*	GBCCH1718-14	KF134551
325	*Longitarsus dorsalis*	GBCCH10204-19	KF652448
326	*Longitarsus dorsalis*	GBCCH10212-19	KF652515
327	*Longitarsus dorsalis*	GBCCH10220-19	KF653273
328	*Longitarsus dorsalis*	GBCCH10230-19	KF653354
329	*Longitarsus dorsalis*	GBCCH10247-19	KF653633
330	*Longitarsus dorsalis*	GBCCH10253-19	KF653675
331	*Longitarsus dorsalis*	GBCCH10256-19	KF653678
332	*Longitarsus dorsalis*	GBCCH10273-19	KF654485
333	*Longitarsus dorsalis*	GBCOU2976-13	KM451023
334	*Longitarsus dorsalis*	GBCCH10194-19	KF653712
335	*Longitarsus dorsalis*	GBCCH10208-19	KF652457
336	*Longitarsus dorsalis*	GBCCH10228-19	KF653341
337	*Longitarsus dorsalis*	GBCCH10244-19	KF653406
338	*Longitarsus dorsalis*	GCOL5893-16	KU917671
339	*Longitarsus dorsalis*	GBCCH10258-19	KF653713
340	*Longitarsus dorsalis*	GBCCH10274-19	DQ155808
341	*Longitarsus dorsalis*	GBCCH10275-19	KP763018
342	*Longitarsus dorsalis*	GBCCH10234-19	KF653378
343	*Longitarsus dorsalis*	GBCCH10251-19	KF653669
344	*Longitarsus dorsalis*	GBCCH10263-19	KF653722
345	*Longitarsus dorsalis*	GBCCH10195-19	KF652449
346	*Longitarsus dorsalis*	GBCCH10211-19	KF652514
347	*Longitarsus dorsalis*	GBCCH10224-19	KF653319
348	*Longitarsus dorsalis*	GBCCH10239-19	KF653401
349	*Longitarsus dorsalis*	GBCCH10260-19	KF653717
350	*Longitarsus dorsalis*	GBCCH10207-19	KF652454
351	*Longitarsus dorsalis*	GBCCH10240-19	KF653402
352	*Longitarsus dorsalis*	GBCCH10248-19	KF653634

353	*Longitarsus dorsalis*	GBCCH10254-19	KF653676
354	*Longitarsus dorsalis*	GBCCH10271-19	KF654099
355	*Longitarsus dorsalis*	GBCCH10235-19	KF653381
356	*Longitarsus dorsalis*	GBCCH10250-19	KF653664
357	*Longitarsus dorsalis*	GBCCH10252-19	KF653674
358	*Longitarsus dorsalis*	GBCCH10268-19	KF654084
359	*Longitarsus dorsalis*	GBCCH1659-14	KF134553
360	*Longitarsus dorsalis*	GBCCH10201-19	KF652442
361	*Longitarsus dorsalis*	GBCCH10227-19	KF653322
362	*Longitarsus dorsalis*	GBCCH10276-19	KX943359
363	*Longitarsus dorsalis*	GBCCH1701-14	KF134567
364	*Longitarsus dorsalis*	GBCCH10231-19	KF653355
365	*Longitarsus dorsalis*	GBCCH10233-19	KF653377
366	*Longitarsus dorsalis*	GBCCH10255-19	KF653677
367	*Longitarsus dorsalis*	GBCCH10261-19	KF653718
368	*Longitarsus dorsalis*	GBCCH10200-19	KF652670
369	*Longitarsus dorsalis*	GBCCH10226-19	KF653321
370	*Longitarsus dorsalis*	GBCCH10249-19	KF653652
371	*Longitarsus dorsalis*	GBCCH10262-19	KF653721
372	*Longitarsus dorsalis*	GBCCH10223-19	KF653318
373	*Longitarsus dorsalis*	GBCCH10266-19	KF653725
374	*Longitarsus dorsalis*	GBCCH10197-19	KF652628
375	*Longitarsus dorsalis*	GBCCH10269-19	KF654097
376	*Longitarsus dorsalis*	GCOL2626-16	KU915282
377	*Longitarsus dorsalis*	GBCCH10246-19	KF653632
378	*Longitarsus dorsalis*	GBCCH10259-19	KF653715
379	*Longitarsus dorsalis*	GBCCH1700-14	KF134557
380	*Longitarsus dorsalis*	GBCOG394-13	KM448396
381	*Longitarsus dorsalis*	GBCCH10192-19	KF653716
382	*Longitarsus dorsalis*	GBCCH10219-19	KF653268
383	*Longitarsus dorsalis*	GBCCH10237-19	KF653399
384	*Longitarsus dorsalis*	GBCCH10272-19	KF654100
385	*Longitarsus dorsalis*	GBCCH1663-14	KF134565
386	*Longitarsus dorsalis*	GBCCH1757-14	KF134563
387	*Longitarsus dorsalis*	GBCCH10210-19	KF652481
388	*Longitarsus dorsalis*	GBCCH10218-19	KF652990
389	*Longitarsus dorsalis*	GBCCH10236-19	KF653398
390	*Longitarsus dorsalis*	GBCCH1662-14	KF134564
391	*Longitarsus dorsalis*	GBCCH10203-19	KF652444
392	*Longitarsus dorsalis*	GBCCH10209-19	KF652467
393	*Longitarsus dorsalis*	GBCCH10221-19	KF653274
394	*Longitarsus dorsalis*	GBCCH10264-19	KF653723
395	*Longitarsus dorsalis*	GCOL6983-16	KU911738
396	*Longitarsus dorsalis*	GBCCH10193-19	KF653714
397	*Longitarsus dorsalis*	GBCCH10196-19	KF652603
398	*Longitarsus dorsalis*	GBCCH10241-19	KF653403
399	*Longitarsus dorsalis*	GBCCH10245-19	KF653407
400	*Longitarsus dorsalis*	GBCCH10257-19	KF653711
401	*Longitarsus dorsalis*	GBCCH10232-19	KF653356
402	*Longitarsus echii*	FBCON357-13	KM439595
403	*Longitarsus echii*	GBCOG451-13	KM439361

404	*Longitarsus echii*	GBCOU3570-13	KM450124
405	*Longitarsus echii*	FBCOB313-10	HQ953939
406	*Longitarsus erro*	OPPQA280-17	
407	*Longitarsus erro*	SMTPO3312-15	MG058556
408	*Longitarsus erro*	SMTPF4348-14	KR488437
409	*Longitarsus erro*	SMTPO5266-15	MG057188
410	*Longitarsus erro*	SMTPO5281-15	MG059755
411	*Longitarsus erro*	SMTPO5282-15	MG054624
412	*Longitarsus erro*	SMTPF1725-14	KR489714
413	*Longitarsus erro*	SMTPF8658-14	KR485637
414	*Longitarsus erro*	SSBAC4315-13	KM842486
415	*Longitarsus erro*	SMTPO3310-15	MG062508
416	*Longitarsus erro*	CNROM1022-13	KR122127
417	*Longitarsus erro*	SMTPB13943-13	KR484486
418	*Longitarsus erro*	SMTPO5263-15	MG054293
419	*Longitarsus erro*	SMTPO5285-15	MG058460
420	*Longitarsus erro*	SSROB6403-14	KR490017
421	*Longitarsus erro*	HEMAY811-12	KR481303
422	*Longitarsus erro*	PHCOL021-11	KR483867
423	*Longitarsus erro*	SMTPF4989-14	KR482198
424	*Longitarsus erro*	SMTPO5280-15	MG056804
425	*Longitarsus erro*	SMTPB22359-13	KR490803
426	*Longitarsus erro*	SMTPO5274-15	MG057208
427	*Longitarsus erro*	HEMAY813-12	KR483661
428	*Longitarsus erro*	JSCOL106-11	KR480663
429	*Longitarsus erro*	SMTPO10435-15	MG058239
430	*Longitarsus erro*	PAAPR1658-12	KR484412
431	*Longitarsus erro*	SSROB5424-14	KR487131
432	*Longitarsus erro*	SMTPO5275-15	MG056144
433	*Longitarsus erro*	ASCMT269-11	MG061065
434	*Longitarsus erro*	SMTPO5284-15	MG061412
435	*Longitarsus erro*	SMTPO5269-15	MG061604
436	*Longitarsus erro*	SMTPO5278-15	MG061694
437	*Longitarsus erro*	SSROB6207-14	KR486831
438	*Longitarsus erro*	SMTPB13938-13	KR485986
439	*Longitarsus erro*	SMTPL3178-15	MG055558
440	*Longitarsus erro*	SMTPO5276-15	MG061927
441	*Longitarsus erro*	SSROB6674-14	KR481474
442	*Longitarsus erro*	SMTPF3606-14	KR490743
443	*Longitarsus erro*	SMTPO5265-15	MG053698
444	*Longitarsus erro*	SSROB5169-14	KR488287
445	*Longitarsus erro*	SSBAC4314-13	KM842063
446	*Longitarsus erro*	SSROB7193-14	KR489093
447	*Longitarsus erro*	BBCCN173-10	JF887838
448	*Longitarsus erro*	SMTPF4987-14	KR480026
449	*Longitarsus erro*	SMTPO5287-15	MG055331
450	*Longitarsus erro*	SMTPF4988-14	KR481185
451	*Longitarsus erro*	SMTPF7088-14	KR490925
452	*Longitarsus erro*	SSROB6661-14	KR491245
453	*Longitarsus erro*	SMTPO5270-15	MG061348
454	*Longitarsus exoletus*		HQ164948

455	*Longitarsus exsoletus*	AMRSG089-16	
456	*Longitarsus exsoletus*	FBCON788-13	KM442966
457	*Longitarsus exsoletus*	FBCOQ152-13	KM447252
458	*Longitarsus exsoletus*	GBCCH10282-19	KF654851
459	*Longitarsus exsoletus*	GBCCH10320-19	KF656257
460	*Longitarsus exsoletus*	GCOL2534-16	KU908319
461	*Longitarsus exsoletus*	COLFG280-13	KJ962683
462	*Longitarsus exsoletus*	GBCCH10319-19	KF656256
463	*Longitarsus exsoletus*	GBCCH10330-19	KF656578
464	*Longitarsus exsoletus*	GBCOG011-13	KM445819
465	*Longitarsus exsoletus*	FBCOK754-13	KM445352
466	*Longitarsus exsoletus*	GBCCH10299-19	KF655713
467	*Longitarsus exsoletus*	GBCCH10302-19	KF655723
468	*Longitarsus exsoletus*	GBCCH10325-19	KF656567
469	*Longitarsus exsoletus*	COLFG279-13	KJ962358
470	*Longitarsus exsoletus*	GBCCH10289-19	KF654863
471	*Longitarsus exsoletus*	GBCCH10291-19	KF654903
472	*Longitarsus exsoletus*	GBCCH10293-19	KF654905
473	*Longitarsus exsoletus*	GBCCH10311-19	KF655989
474	*Longitarsus exsoletus*	GBCCH10317-19	KF656202
475	*Longitarsus exsoletus*	GBCCH10327-19	KF656575
476	*Longitarsus exsoletus*	GBCCH10332-19	KF656587
477	*Longitarsus exsoletus*	GBCCH10303-19	KF655724
478	*Longitarsus exsoletus*	GBCCH10336-19	KX943418
479	*Longitarsus exsoletus*	GCOL11823-16	KU917527
480	*Longitarsus exsoletus*	GBCCH10277-19	HQ164948
481	*Longitarsus exsoletus*	GBCCH10313-19	KF656198
482	*Longitarsus exsoletus*	MEDLB500-12	MH323191
483	*Longitarsus exsoletus*	GBCCH10316-19	KF656201
484	*Longitarsus exsoletus*	GBCCH10331-19	KF656585
485	*Longitarsus exsoletus*	GBCOD697-13	KM452670
486	*Longitarsus exsoletus*	COLFE1508-13	KJ962586
487	*Longitarsus exsoletus*	GBCCH10281-19	KF654850
488	*Longitarsus exsoletus*	GBCCH10300-19	KF655714
489	*Longitarsus exsoletus*	GBCCH10304-19	KF655736
490	*Longitarsus exsoletus*	COLFE1506-13	KJ963703
491	*Longitarsus exsoletus*	GBCCH10307-19	KF655762
492	*Longitarsus exsoletus*	GBCCH10315-19	KF656200
493	*Longitarsus exsoletus*	GBCCH10334-19	KP306812
494	*Longitarsus exsoletus*	GCOL7025-16	KU907302
495	*Longitarsus exsoletus*	GBCCH10312-19	KF656197
496	*Longitarsus exsoletus*	GBCCH10335-19	KP763063
497	*Longitarsus exsoletus*	GCOL2417-16	KU912908
498	*Longitarsus exsoletus*	MEDLB498-12	MH323190
499	*Longitarsus exsoletus*	GBCCH10285-19	KF654859
500	*Longitarsus exsoletus*	GBCCH10301-19	KF655715
501	*Longitarsus exsoletus*	GBCCH10323-19	KF656503
502	*Longitarsus exsoletus*	GBCCH10328-19	KF656576
503	*Longitarsus exsoletus*	MEDLB439-12	MH323189
504	*Longitarsus exsoletus*	COLFG281-13	KJ964929
505	*Longitarsus exsoletus*	FBCOB337-10	HQ953959

506	*Longitarsus exsoletus*	GBCCH10283-19	KF654852
507	*Longitarsus exsoletus*	FBCOP370-13	KM449417
508	*Longitarsus exsoletus*	GBCCH10296-19	KF655703
509	*Longitarsus exsoletus*	GBCCH10308-19	KF655763
510	*Longitarsus exsoletus*	GBCCH10324-19	KF656548
511	*Longitarsus exsoletus*	GBCCH10329-19	KF656577
512	*Longitarsus exsoletus*	GBCCH10337-19	MH020464
513	*Longitarsus exsoletus*	GCOL2714-16	KU916041
514	*Longitarsus exsoletus*	GBCCH10305-19	KF655737
515	*Longitarsus exsoletus*	MEDLB441-12	MH323188
516	*Longitarsus exsoletus*	GBCCH10280-19	KF656568
517	*Longitarsus exsoletus*	GBCCH10297-19	KF655704
518	*Longitarsus exsoletus*	GBCCH10310-19	KF655810
519	*Longitarsus exsoletus*	GBCCH10314-19	KF656199
520	*Longitarsus exsoletus*	GBCCH10284-19	KF654853
521	*Longitarsus exsoletus*	GBCCH10318-19	KF656255
522	*Longitarsus exsoletus*	GCOL11816-16	KU915867
523	*Longitarsus exsoletus*	MEDLB442-12	MH323192
524	*Longitarsus exsoletus*	GBCCH10287-19	KF654861
525	*Longitarsus exsoletus*	GBCCH10306-19	KF655738
526	*Longitarsus exsoletus*	GBCCH10309-19	KF655788
527	*Longitarsus exsoletus*	GBCCH10322-19	KF656502
528	*Longitarsus exsoletus*	GBCCH10279-19	KF656586
529	*Longitarsus exsoletus*	GBCCH10292-19	KF654904
530	*Longitarsus exsoletus*	GBCOD696-13	KM443313
531	*Longitarsus exsoletus*	GBCCH10286-19	KF654860
532	*Longitarsus exsoletus*	GBCCH10290-19	KF654902
533	*Longitarsus exsoletus*	GBCCH10294-19	KF655701
534	*Longitarsus exsoletus*	GBCCH10295-19	KF655702
535	*Longitarsus exsoletus*	GBCCH10298-19	KF655705
536	*Longitarsus exsoletus*	GBCCH10326-19	KF656569
537	*Longitarsus exsoletus*	FBCOK755-13	KM442167
538	*Longitarsus exsoletus*	GBCCH10288-19	KF654862
539	*Longitarsus exsoletus*	GBCCH10321-19	KF656296
540	*Longitarsus exsoletus*	GBCCH10333-19	KU697479
541	*Longitarsus exsoletus*	MEDLB470-12	MH323187
542	*Longitarsus exsoletus*	GBCCH10278-19	AM283194
543	*Longitarsus exsoletus*	GBCOU2485-13	KM451563
544	*Longitarsus fallax*		MK893903
545	*Longitarsus ferruginipennis*	GBCCH10339-19	KF653461
546	*Longitarsus ferruginipennis*	GBCCH10340-19	KP763022
547	*Longitarsus ferruginipennis*	GBCCH10338-19	KF653454
548	*Longitarsus flavicornis*	GBCCH10341-19	HQ164779
549	*Longitarsus foudrasi*		MK893904
550	*Longitarsus fulgens*	FBCOB1030-10	HQ953772
551	*Longitarsus gracilis*	SMTPR10111-16	MG057846
552	*Longitarsus gracilis*	GCOL2735-16	KU916730
553	*Longitarsus gracilis*	GCOL5238-16	KU915449
554	*Longitarsus gracilis*	GCOL2734-16	KU914044
555	*Longitarsus helvolus*	GCOL2578-16	KU916658
556	*Longitarsus helvolus*	GCOL2645-16	KU916246

557	*Longitarsus helvolus*	GCOL2648-16	KU916133
558	*Longitarsus helvolus*	GCOL2649-16	KU913464
559	*Longitarsus holsaticus*	FBCOB614-10	HQ954167
560	*Longitarsus holsaticus*	COLFE1148-13	KJ965710
561	*Longitarsus ibericus*	GBCCH1636-14	KF134582
562	*Longitarsus ibericus*	GBCCH10342-19	KX943455
563	*Longitarsus isoplexidis*		MK893905
564	*Longitarsus jacobaeae*	INRMA2758-15	
565	*Longitarsus jacobaeae*	INRMA2760-15	
566	*Longitarsus jacobaeae*	INRMA2759-15	
567	*Longitarsus jacobaeae*	INRMA2757-15	
568	*Longitarsus jacobaeae*	GCOL5459-16	KU907539
569	*Longitarsus jacobaeae*	SMTPP2572-15	MG057485
570	*Longitarsus jacobaeae*	SMTPR8277-16	MG055773
571	*Longitarsus jacobaeae*	GCOL2684-16	KU915306
572	*Longitarsus jacobaeae*	GCOL2593-16	KU911886
573	*Longitarsus jacobaeae*	GCOL5237-16	KU914513
574	*Longitarsus kutscherae*	GCOL2542-16	KU907374
575	*Longitarsus kutscherae*	GCOL2690-16	KU917553
576	*Longitarsus kutscherae*	GCOL2541-16	KU906881
577	*Longitarsus languidus*	GCOL13204-16	KU906343
578	*Longitarsus languidus*	GCOL2594-16	KU907689
579	*Longitarsus lateripunctatus*		MK893906
580	*Longitarsus lewisii*	COLFE1523-13	KJ963996
581	*Longitarsus lewisii*	GCOL1801-16	KU908551
582	*Longitarsus lewisii*	SMTPR5625-16	MG060298
583	*Longitarsus lewisii*	GCOL2493-16	KU909091
584	*Longitarsus lewisii*	SMTPR5626-16	MG056231
585	*Longitarsus lewisii*	COLFE1521-13	KJ964861
586	*Longitarsus lewisii*	COLFE1522-13	KJ963504
587	*Longitarsus lewisii*	GCOL13512-16	KU906308
588	*Longitarsus lewisii*	GCOL2480-16	KU906165
589	*Longitarsus lewisii*	GCOL2543-16	KU909736
590	*Longitarsus lewisii*	GCOL2505-16	KU916232
591	*Longitarsus lewisii*	SMTPR5624-16	MG059587
592	*Longitarsus lewisii*	SMTPR5614-16	MG059785
593	*Longitarsus lewisii*	SMTPR5616-16	MG055894
594	*Longitarsus linnaei*		MK893908
595	*Longitarsus longipennis*	AMRSG005-16	
596	*Longitarsus longipennis*	AMRSG068-16	
597	*Longitarsus longipennis*	AMRSG011-16	
598	*Longitarsus longipennis*	AMRSG013-16	
599	*Longitarsus longipennis*	AMRSG025-16	
600	*Longitarsus longipennis*	AMRSG024-16	
601	*Longitarsus longiseta*	COLFD190-12	KJ962038
602	*Longitarsus longiseta*	COLFE711-13	KJ967257
603	*Longitarsus longiseta*	COLFD793-12	KJ961838
604	*Longitarsus luridus*	OPPEI2982-17	
605	*Longitarsus luridus*	CNTNJ864-14	
606	*Longitarsus luridus*	OPPEI2985-17	
607	*Longitarsus luridus*	OPPEI3030-17	

608	*Longitarsus luridus*	OPPEI3035-17	
609	*Longitarsus luridus*	OPPEG3353-17	
610	*Longitarsus luridus*	OPPEI2961-17	
611	*Longitarsus luridus*	OPPEI3015-17	
612	*Longitarsus luridus*	OPPDI2017-17	
613	*Longitarsus luridus*	OPPEG3381-17	
614	*Longitarsus luridus*	OPPEI2948-17	
615	*Longitarsus luridus*	OPPEI3013-17	
616	*Longitarsus luridus*	OPPEG3356-17	
617	*Longitarsus luridus*	OPPEI3012-17	
618	*Longitarsus luridus*	OPPEI3009-17	
619	*Longitarsus luridus*	OPPEG3391-17	
620	*Longitarsus luridus*	OPPEG3394-17	
621	*Longitarsus luridus*	OPPEI2989-17	
622	*Longitarsus luridus*	OPPEG3395-17	
623	*Longitarsus luridus*	OPPEI3039-17	
624	*Longitarsus luridus*	CNTNJ848-14	KR120663
625	*Longitarsus luridus*	CNTNJ852-14	KR122373
626	*Longitarsus luridus*	FBCOF855-12	KM448715
627	*Longitarsus luridus*	GBCCH10349-19	KF654855
628	*Longitarsus luridus*	MEDLB564-12	MH323194
629	*Longitarsus luridus*	RRSSC3397-15	MG060759
630	*Longitarsus luridus*	CNTNK668-14	KR131101
631	*Longitarsus luridus*	FBCOI604-12	KM450361
632	*Longitarsus luridus*	GBCCH1737-14	KF134572
633	*Longitarsus luridus*	GCOL11962-16	KU913563
634	*Longitarsus luridus*	CNBRH235-14	KR123152
635	*Longitarsus luridus*	CNTNJ860-14	KR119207
636	*Longitarsus luridus*	CNTNJ862-14	KR123803
637	*Longitarsus luridus*	GBCCH10343-19	HQ165086
638	*Longitarsus luridus*	GBCCH1703-14	KF134577
639	*Longitarsus luridus*	RBINA1129-13	KR484403
640	*Longitarsus luridus*	RRSSC7014-15	MG056720
641	*Longitarsus luridus*	CNTNJ851-14	KR127953
642	*Longitarsus luridus*	CNTNK665-14	KR119120
643	*Longitarsus luridus*	COLFE1472-13	KJ965803
644	*Longitarsus luridus*	GBCCH1724-14	KF134586
645	*Longitarsus luridus*	RRSSC5061-15	MG060921
646	*Longitarsus luridus*	RRSSC7011-15	MG060502
647	*Longitarsus luridus*	CNTNI1577-14	KR130528
648	*Longitarsus luridus*	CNTNI1596-14	KR128307
649	*Longitarsus luridus*	CNTNJ849-14	KR126712
650	*Longitarsus luridus*	GBCCH10351-19	KF654857
651	*Longitarsus luridus*	GBCCH10345-19	KF654795
652	*Longitarsus luridus*	GBCCH1727-14	KF134589
653	*Longitarsus luridus*	GBCOU1325-13	KM445211
654	*Longitarsus luridus*	CNTNI1578-14	KR121993
655	*Longitarsus luridus*	CNTNJ863-14	KR122822
656	*Longitarsus luridus*	GBCCH10354-19	KF655341
657	*Longitarsus luridus*	GBCCH10358-19	KF656641
658	*Longitarsus luridus*	HENOV009-12	KR481184

659	*Longitarsus luridus*	SMTPP4328-15	MG054230
660	*Longitarsus luridus*	GBCCH10355-19	KF656203
661	*Longitarsus luridus*	GBCCH1637-14	KF134578
662	*Longitarsus luridus*	GBCCH1736-14	KF134571
663	*Longitarsus luridus*	GBCOU2040-13	KM439860
664	*Longitarsus luridus*	SMTPP2425-15	MG062249
665	*Longitarsus luridus*	CNFNN540-14	KR122263
666	*Longitarsus luridus*	SMTPP1113-15	MG057960
667	*Longitarsus luridus*	SMTPP1140-15	MG053913
668	*Longitarsus luridus*	COLFB606-12	KJ961791
669	*Longitarsus luridus*	GBCCH1728-14	KF134590
670	*Longitarsus luridus*	GCOL2674-16	KU913944
671	*Longitarsus luridus*	GBCCH10357-19	KF656581
672	*Longitarsus luridus*	GCOL11960-16	KU914314
673	*Longitarsus luridus*	CNGBH658-14	KR128939
674	*Longitarsus luridus*	GBCCH10356-19	KF656259
675	*Longitarsus luridus*	GCOL2622-16	KU918572
676	*Longitarsus luridus*	PHCOL114-11	KR490736
677	*Longitarsus luridus*	RRSSC7028-15	MG058737
678	*Longitarsus luridus*	SMTPO1594-15	MG053866
679	*Longitarsus luridus*	SSROB6198-14	KR488616
680	*Longitarsus luridus*	CNTNI1589-14	KR128969
681	*Longitarsus luridus*	GBCCH1725-14	KF134587
682	*Longitarsus luridus*	GCOL12517-16	KU914776
683	*Longitarsus luridus*	RRSSC7017-15	MG053697
684	*Longitarsus luridus*	SMTPP4324-15	MG060131
685	*Longitarsus luridus*	COLFD829-12	KJ967400
686	*Longitarsus luridus*	FBCOD960-11	KM441466
687	*Longitarsus luridus*	GBCCH10344-19	KF656594
688	*Longitarsus luridus*	GCOL2435-16	KU911297
689	*Longitarsus luridus*	CNTNI1590-14	KR124936
690	*Longitarsus luridus*	COLFD438-12	KJ962293
691	*Longitarsus luridus*	FBCOC777-10	JF889787
692	*Longitarsus luridus*	GBCCH10359-19	KP763107
693	*Longitarsus luridus*	SMTPO8831-15	MG060828
694	*Longitarsus luridus*	CNBRH236-14	KR128356
695	*Longitarsus luridus*	CNBRH238-14	KR119567
696	*Longitarsus luridus*	CNTNI1579-14	KR130867
697	*Longitarsus luridus*	CNTNJ850-14	KR125515
698	*Longitarsus luridus*	FBCOH602-12	KM451908
699	*Longitarsus luridus*	GBCCH10347-19	KF654797
700	*Longitarsus luridus*	GBCCH10360-19	KX943364
701	*Longitarsus luridus*	GCOL2688-16	KU908764
702	*Longitarsus luridus*	RRSSC7023-15	MG058682
703	*Longitarsus luridus*	SMTPO3493-15	MG060353
704	*Longitarsus luridus*	CNTNK669-14	KR120371
705	*Longitarsus luridus*	GBCCH10348-19	KF654854
706	*Longitarsus luridus*	GBCCH1721-14	KF134583
707	*Longitarsus luridus*	SMTPB17013-13	KR481430
708	*Longitarsus luridus*	SMTPB17019-13	KR489660
709	*Longitarsus luridus*	SMTPJ1035-14	KR484048

710	*Longitarsus luridus*	GBCCH1639-14	KF134580
711	*Longitarsus luridus*	GBCCH1726-14	KF134588
712	*Longitarsus luridus*	GCOL8976-16	KU906478
713	*Longitarsus luridus*	AGAKP294-17	MG058066
714	*Longitarsus luridus*	CNBRH232-14	KR123264
715	*Longitarsus luridus*	CNTNI1586-14	KR123652
716	*Longitarsus luridus*	CNTNK666-14	KR126242
717	*Longitarsus luridus*	GBCCH10353-19	KF655340
718	*Longitarsus luridus*	GBCCH1702-14	KF134576
719	*Longitarsus luridus*	GCOL2486-16	KU918878
720	*Longitarsus luridus*	GCOL2719-16	KU907806
721	*Longitarsus luridus*	BBCEC385-10	HQ551558
722	*Longitarsus luridus*	CNTNI1592-14	KR128362
723	*Longitarsus luridus*	CNTNJ855-14	KR128910
724	*Longitarsus luridus*	CNTNK667-14	KR124289
725	*Longitarsus luridus*	GBCCH1638-14	KF134579
726	*Longitarsus luridus*	MEDLB436-12	MH323193
727	*Longitarsus luridus*	SMTPR3838-16	MG061542
728	*Longitarsus luridus*	COLFE1471-13	KJ967377
729	*Longitarsus luridus*	GBCCH10346-19	KF654796
730	*Longitarsus luridus*	GBCCH10350-19	KF654856
731	*Longitarsus luridus*	GBCCH1723-14	KF134585
732	*Longitarsus luridus*	GBCCH1738-14	KF134573
733	*Longitarsus luridus*	MEDLB437-12	MH323195
734	*Longitarsus luridus*	RRINV1922-15	KT708605
735	*Longitarsus luridus*	CNGBH647-14	KR130297
736	*Longitarsus luridus*	CNTNI1587-14	KR126756
737	*Longitarsus luridus*	CNTNJ861-14	KR127636
738	*Longitarsus luridus*	GBCCH1722-14	KF134584
739	*Longitarsus luridus*	RRSSC7012-15	MG059993
740	*Longitarsus luridus*	RRSSC7016-15	MG060155
741	*Longitarsus luridus*	SMTPR3847-16	MG058544
742	*Longitarsus luridus*	CNTNI1593-14	KR128206
743	*Longitarsus luridus*	GBCCH10352-19	KF654858
744	*Longitarsus luridus*	GCOL2464-16	KU915662
745	*Longitarsus lycopi*	GBCCH10365-19	KF653668
746	*Longitarsus lycopi*	GBCCH10364-19	KF653667
747	*Longitarsus lycopi*	GBCCH10362-19	KF653599
748	*Longitarsus lycopi*	GCOL2738-16	KU909570
749	*Longitarsus lycopi*	FBCOB538-10	HQ954113
750	*Longitarsus lycopi*	GCOL5262-16	KU911216
751	*Longitarsus lycopi*	GBCCH10366-19	KF653671
752	*Longitarsus lycopi*	GBCCH10363-19	KF653605
753	*Longitarsus lycopi*	GBCCH10368-19	KX943332
754	*Longitarsus lycopi*	GCOL2718-16	KU907616
755	*Longitarsus lycopi*	GBCCH10361-19	KF653347
756	*Longitarsus lycopi*	GBCCH10367-19	KF654534
757	*Longitarsus melanocephalus*	GBCCH10371-19	KF656339
758	*Longitarsus melanocephalus*	COLFD659-12	KJ965048
759	*Longitarsus melanocephalus*	COLFD797-12	KJ963049
760	*Longitarsus melanocephalus*	FBCOA197-10	KM448415

761	*Longitarsus melanocephalus*	COLFD658-12	KJ964850
762	*Longitarsus melanocephalus*	GBCOL034-12	KM446690
763	*Longitarsus melanocephalus*	GCOL2434-16	KU916745
764	*Longitarsus melanocephalus*	COLFD806-12	KJ966105
765	*Longitarsus melanocephalus*	COLFE1605-13	KJ963029
766	*Longitarsus melanocephalus*	COLFE1606-13	KJ962770
767	*Longitarsus melanocephalus*	GBCCH10372-19	KF656515
768	*Longitarsus melanocephalus*	FBCOO713-13	KM445664
769	*Longitarsus melanocephalus*	GBCCH10373-19	KF656556
770	*Longitarsus melanocephalus*	GBCCH10369-19	KF656384
771	*Longitarsus melanocephalus*	GCOL2483-16	KU911419
772	*Longitarsus melanocephalus*	COLFA312-12	KJ963328
773	*Longitarsus melanocephalus*	GBCCH10374-19	KF656557
774	*Longitarsus melanocephalus*	FBCOQ047-13	KM451334
775	*Longitarsus melanocephalus*	GCOL2462-16	KU912501
776	*Longitarsus melanocephalus*	FBCOG939-12	KM444736
777	*Longitarsus melanocephalus*	GBCCH10375-19	KF656579
778	*Longitarsus melanocephalus*	GBCCH10370-19	KF655010
779	*Longitarsus melanocephalus*	GCOL5256-16	KU908529
780	*Longitarsus melanocephalus*	FBCOD961-11	KM444234
781	*Longitarsus melanocephalus*	GBCOD186-13	KM446110
782	*Longitarsus melanocephalus*	GCOL5261-16	KU916447
783	*Longitarsus melanocephalus*	GBCCH10376-19	KX943469
784	*Longitarsus melanocephalus*	GBCOD221-13	KM441949
785	*Longitarsus melanocephalus*	FBCOK604-13	KM444679
786	*Longitarsus melanocephalus*	GCOL2478-16	KU914299
787	*Longitarsus membranaceus*	GBCCH10380-19	KF652954
788	*Longitarsus membranaceus*	GBCCH10396-19	DQ156004
789	*Longitarsus membranaceus*	GBCCH10383-19	KF652957
790	*Longitarsus membranaceus*	GBCCH10393-19	KF653707
791	*Longitarsus membranaceus*	GBCCH10388-19	KF652974
792	*Longitarsus membranaceus*	GBCCH10394-19	KF653708
793	*Longitarsus membranaceus*	GBCCH10401-19	KF656635
794	*Longitarsus membranaceus*	GBCCH10398-19	KF656611
795	*Longitarsus membranaceus*	GBCCH10402-19	KF656636
796	*Longitarsus membranaceus*	GBCCH10404-19	KF656652
797	*Longitarsus membranaceus*	GBCCH10384-19	KF652970
798	*Longitarsus membranaceus*	GBCCH10389-19	KF653501
799	*Longitarsus membranaceus*	GBCCH10406-19	KX943473
800	*Longitarsus membranaceus*	GBCCH10382-19	KF652956
801	*Longitarsus membranaceus*	GBCCH10399-19	KF656619
802	*Longitarsus membranaceus*	GBCCH10379-19	KF652953
803	*Longitarsus membranaceus*	GBCCH10395-19	KF653709
804	*Longitarsus membranaceus*	GBCCH10377-19	KF652951
805	*Longitarsus membranaceus*	GBCCH10390-19	KF653684
806	*Longitarsus membranaceus*	GBCCH10392-19	KF653706
807	*Longitarsus membranaceus*	GBCCH10405-19	KF656661
808	*Longitarsus membranaceus*	GBCCH10386-19	KF652972
809	*Longitarsus membranaceus*	GBCCH10397-19	KF656618
810	*Longitarsus membranaceus*	GBCCH10385-19	KF652971
811	*Longitarsus membranaceus*	GBCCH10387-19	KF652973

812	*Longitarsus membranaceus*	GBCCH10391-19	KF653705
813	*Longitarsus membranaceus*	GBCCH10378-19	KF652952
814	*Longitarsus membranaceus*	GBCCH10403-19	KF656640
815	*Longitarsus membranaceus*	GBCCH10381-19	KF652955
816	*Longitarsus membranaceus*	GBCCH10400-19	KF656627
817	*Longitarsus minusculus*	FBCOP716-13	KM448968
818	*Longitarsus minusculus*	GBCOF884-13	KM442213
819	*Longitarsus minusculus*	GBCCH10408-19	KF653323
820	*Longitarsus minusculus*	GBCOF885-13	KM439395
821	*Longitarsus minusculus*	FBCOP717-13	KM445851
822	*Longitarsus minusculus*	GBCCH10407-19	KF652641
823	*Longitarsus minusculus*	GBCCH10409-19	KF653382
824	*Longitarsus minusculus*	GBCCH10410-19	KP763011
825	*Longitarsus minusculus*	FBCOQ018-13	KM444827
826	*Longitarsus minusculus*	GCOL2616-16	KU907958
827	*Longitarsus nanus*	GCOL2666-16	KU917715
828	*Longitarsus nasturtii*	FBCOB610-10	KM442400
829	*Longitarsus nasturtii*	GBCOU3564-13	KM444937
830	*Longitarsus nasturtii*	FBCOA229-10	KM442304
831	*Longitarsus nasturtii*	GBCCH10411-19	MH271196
832	*Longitarsus niger*	GBCCH10412-19	KP763081
833	*Longitarsus niger*	GBCCH10413-19	KX943504
834	*Longitarsus nigerrimus*	FBCOO401-13	KM450462
835	*Longitarsus nigerrimus*	COLFE1007-13	KJ963122
836	*Longitarsus nigerrimus*	COLFC826-12	KJ966220
837	*Longitarsus nigerrimus*	GBCOE027-13	KM443315
838	*Longitarsus nigerrimus*	FBCOE401-12	KM447058
839	*Longitarsus nigerrimus*	FBCOA172-10	HQ948200
840	*Longitarsus nigerrimus*	FBCOO454-13	KM451930
841	*Longitarsus nigerrimus*	GBCOE071-13	KM443860
842	*Longitarsus nigripennis*	GBCCH10414-19	MF495677
843	*Longitarsus nigrocillus*	GBCCH10415-19	KX943464
844	*Longitarsus nigrocillus*	GBCCH1739-14	KF134592
845	*Longitarsus nigrocillus*	GBCCH1749-14	KF134591
846	*Longitarsus nigrofasciatus*	GMGMI949-14	
847	*Longitarsus nigrofasciatus*	GBCCH10429-19	KF655097
848	*Longitarsus nigrofasciatus*	GCOL2610-16	KU907531
849	*Longitarsus nigrofasciatus*	GBCCH10427-19	KF654906
850	*Longitarsus nigrofasciatus*	GCOL2651-16	KU919171
851	*Longitarsus nigrofasciatus*	GBCCH10416-19	KF652918
852	*Longitarsus nigrofasciatus*	GBCCH10431-19	KF655146
853	*Longitarsus nigrofasciatus*	GBCCH10423-19	KF653002
854	*Longitarsus nigrofasciatus*	GBCCH10434-19	KX943438
855	*Longitarsus nigrofasciatus*	GBCCH10425-19	KF654886
856	*Longitarsus nigrofasciatus*	GBCCH10426-19	KF654887
857	*Longitarsus nigrofasciatus*	GBCCH10430-19	KF655145
858	*Longitarsus nigrofasciatus*	GBCCH10417-19	KF652942
859	*Longitarsus nigrofasciatus*	GBCCH10428-19	KF655076
860	*Longitarsus nigrofasciatus*	GBCCH10433-19	KP763073
861	*Longitarsus nigrofasciatus*	GCOL2576-16	KU910986
862	*Longitarsus nigrofasciatus*	GBCCH10422-19	KF653001

863	*Longitarsus nigrofasciatus*	GBCCH10419-19	KF652997
864	*Longitarsus nigrofasciatus*	GBCCH10420-19	KF652998
865	*Longitarsus nigrofasciatus*	GBCCH10421-19	KF652999
866	*Longitarsus nigrofasciatus*	GBCCH10432-19	KF655388
867	*Longitarsus nigrofasciatus*	GBCCH10418-19	KF652943
868	*Longitarsus nigrofasciatus*	GBCCH10424-19	KF653710
869	*Longitarsus obliteratus*	FBCOP711-13	KM441382
870	*Longitarsus obliteratus*	GBCOF881-13	KM448002
871	*Longitarsus obliteratus*	GCOL2577-16	KU914564
872	*Longitarsus obliteratus*	GCOL2664-16	KU910608
873	*Longitarsus obliteratus*	GCOL2715-16	KU907822
874	*Longitarsus obliteratus*	FBCOK294-13	KM448242
875	*Longitarsus obliteratus*	GCOL10288-16	KU914104
876	*Longitarsus obliteratus*	GCOL2665-16	KU916904
877	*Longitarsus obliteratus*	GBCOU3549-13	KM447410
878	*Longitarsus obliteratus*	GCOL5314-16	KU910056
879	*Longitarsus obliteratus*	GCOL5315-16	KU918904
880	*Longitarsus obliteratus*	GCOL2621-16	KU913143
881	*Longitarsus obliteratus*	GBCOU3517-13	KM450525
882	*Longitarsus obliteratus*	FBCOP710-13	KM445197
883	*Longitarsus obliteratus*	GCOL10285-16	KU908711
884	*Longitarsus ochroleucus*	GMGRG3968-13	
885	*Longitarsus ochroleucus*	GBCCH10443-19	KF653155
886	*Longitarsus ochroleucus*	GBCCH1758-14	KF134552
887	*Longitarsus ochroleucus*	GBCCH10439-19	KF652554
888	*Longitarsus ochroleucus*	FBCOK730-13	KM444273
889	*Longitarsus ochroleucus*	GBCCH10444-19	KF653645
890	*Longitarsus ochroleucus*	GBCCH10441-19	KF653153
891	*Longitarsus ochroleucus*	FBCOK637-13	KM449566
892	*Longitarsus ochroleucus*	GBCCH10435-19	HQ164752
893	*Longitarsus ochroleucus*	FBCOA866-10	KM441446
894	*Longitarsus ochroleucus*	GBCCH10446-19	KF653647
895	*Longitarsus ochroleucus*	GBCCH10437-19	KF652668
896	*Longitarsus ochroleucus*	GBCCH10440-19	KF652788
897	*Longitarsus ochroleucus*	GBCOD188-13	KM448633
898	*Longitarsus ochroleucus*	GBCCH10442-19	KF653154
899	*Longitarsus ochroleucus*	GBCOF617-13	KM449710
900	*Longitarsus ochroleucus*	GBCCH10447-19	KF653656
901	*Longitarsus ochroleucus*	GBCOG010-13	KM447486
902	*Longitarsus ochroleucus*	GCOL7466-16	KU910625
903	*Longitarsus ochroleucus*	GBCCH10448-19	KP762981
904	*Longitarsus ochroleucus*	GBCCH10436-19	KF652639
905	*Longitarsus ochroleucus*	GBCCH10438-19	KF652505
906	*Longitarsus ochroleucus*	FBCOQ148-13	KM443981
907	*Longitarsus ochroleucus*	GBCOD187-13	KM441507
908	*Longitarsus ochroleucus*	FBCOK731-13	KM441904
909	*Longitarsus ochroleucus*	GBCCH10445-19	KF653646
910	*Longitarsus ochroleucus*	GBCCH1759-14	KF134562
911	*Longitarsus ochroleucus lindbergi*		MK893907
912	*Longitarsus ordinatus*	GBCCH10467-19	KF653127
913	*Longitarsus ordinatus*	GBCCH10475-19	KF653245

914	*Longitarsus ordinatus*	GBCCH10503-19	KF653579
915	*Longitarsus ordinatus*	GBCCH10518-19	KF653600
916	*Longitarsus ordinatus*	GBCCH10519-19	KF653601
917	*Longitarsus ordinatus*	GBCCH10570-19	KF653742
918	*Longitarsus ordinatus*	GBCCH10576-19	KF653749
919	*Longitarsus ordinatus*	GBCCH10601-19	KF654552
920	*Longitarsus ordinatus*	GBCCH10605-19	KF654574
921	*Longitarsus ordinatus*	GBCCH10608-19	KF654593
922	*Longitarsus ordinatus*	GBCCH10491-19	KF653480
923	*Longitarsus ordinatus*	GBCCH10482-19	KF653363
924	*Longitarsus ordinatus*	GBCCH10495-19	KF653484
925	*Longitarsus ordinatus*	GBCCH10543-19	KF653692
926	*Longitarsus ordinatus*	GBCCH10548-19	KF653697
927	*Longitarsus ordinatus*	GBCCH10557-19	KF653727
928	*Longitarsus ordinatus*	GBCCH10471-19	KF653157
929	*Longitarsus ordinatus*	GBCCH10486-19	KF653367
930	*Longitarsus ordinatus*	GBCCH10507-19	KF653588
931	*Longitarsus ordinatus*	GBCCH10533-19	KF653681
932	*Longitarsus ordinatus*	GBCCH10536-19	KF653685
933	*Longitarsus ordinatus*	GBCCH10539-19	KF653688
934	*Longitarsus ordinatus*	GBCCH10591-19	KF653873
935	*Longitarsus ordinatus*	GBCCH1664-14	KF134544
936	*Longitarsus ordinatus*	GBCCH10465-19	KF653125
937	*Longitarsus ordinatus*	GBCCH10483-19	KF653364
938	*Longitarsus ordinatus*	GBCCH10488-19	KF653369
939	*Longitarsus ordinatus*	GBCCH10517-19	KF653598
940	*Longitarsus ordinatus*	GBCCH10535-19	KF653683
941	*Longitarsus ordinatus*	GBCCH10560-19	KF653731
942	*Longitarsus ordinatus*	GBCCH10582-19	KF653755
943	*Longitarsus ordinatus*	GBCCH10584-19	KF653822
944	*Longitarsus ordinatus*	GBCCH10452-19	KF653740
945	*Longitarsus ordinatus*	GBCCH10454-19	KF652597
946	*Longitarsus ordinatus*	GBCCH10464-19	KF653124
947	*Longitarsus ordinatus*	GBCCH10529-19	KF653666
948	*Longitarsus ordinatus*	GBCCH10542-19	KF653691
949	*Longitarsus ordinatus*	GBCCH10590-19	KF653872
950	*Longitarsus ordinatus*	GBCCH10599-19	KF654550
951	*Longitarsus ordinatus*	GBCCH10481-19	KF653361
952	*Longitarsus ordinatus*	GBCCH10502-19	KF653537
953	*Longitarsus ordinatus*	GBCCH10521-19	KF653603
954	*Longitarsus ordinatus*	GBCCH10534-19	KF653682
955	*Longitarsus ordinatus*	GBCCH10537-19	KF653686
956	*Longitarsus ordinatus*	GBCCH10573-19	KF653745
957	*Longitarsus ordinatus*	GBCCH10574-19	KF653747
958	*Longitarsus ordinatus*	GBCCH10597-19	KF654532
959	*Longitarsus ordinatus*	GBCCH10453-19	KF654627
960	*Longitarsus ordinatus*	GBCCH10462-19	KF652575
961	*Longitarsus ordinatus*	GBCCH10490-19	KF653380
962	*Longitarsus ordinatus*	GBCCH10541-19	KF653690
963	*Longitarsus ordinatus*	GBCCH10564-19	KF653735
964	*Longitarsus ordinatus*	GBCCH10580-19	KF653753

965	*Longitarsus ordinatus*	GBCCH10588-19	KF653833
966	*Longitarsus ordinatus*	GBCCH10612-19	KF655944
967	*Longitarsus ordinatus*	GBCCH10613-19	KP763012
968	*Longitarsus ordinatus*	GBCCH10461-19	KF652572
969	*Longitarsus ordinatus*	GBCCH10520-19	KF653602
970	*Longitarsus ordinatus*	GBCCH10552-19	KF653701
971	*Longitarsus ordinatus*	GBCCH10565-19	KF653736
972	*Longitarsus ordinatus*	GBCCH10569-19	KF653741
973	*Longitarsus ordinatus*	GBCCH10592-19	KF653874
974	*Longitarsus ordinatus*	GBCCH10603-19	KF654564
975	*Longitarsus ordinatus*	GBCCH10614-19	KX943383
976	*Longitarsus ordinatus*	GBCCH10484-19	KF653365
977	*Longitarsus ordinatus*	GBCCH10492-19	KF653481
978	*Longitarsus ordinatus*	GBCCH10496-19	KF653485
979	*Longitarsus ordinatus*	GBCCH10498-19	KF653487
980	*Longitarsus ordinatus*	GBCCH10504-19	KF653580
981	*Longitarsus ordinatus*	GBCCH10456-19	KF652599
982	*Longitarsus ordinatus*	GBCCH10457-19	KF652604
983	*Longitarsus ordinatus*	GBCCH10458-19	KF652436
984	*Longitarsus ordinatus*	GBCCH10469-19	KF653129
985	*Longitarsus ordinatus*	GBCCH10476-19	KF653324
986	*Longitarsus ordinatus*	GBCCH10479-19	KF653359
987	*Longitarsus ordinatus*	GBCCH10524-19	KF653607
988	*Longitarsus ordinatus*	GBCCH10545-19	KF653694
989	*Longitarsus ordinatus*	GBCCH10550-19	KF653699
990	*Longitarsus ordinatus*	GBCCH10566-19	KF653737
991	*Longitarsus ordinatus*	GBCCH10474-19	KF653231
992	*Longitarsus ordinatus*	GBCCH10497-19	KF653486
993	*Longitarsus ordinatus*	GBCCH10506-19	KF653587
994	*Longitarsus ordinatus*	GBCCH10511-19	KF653592
995	*Longitarsus ordinatus*	GBCCH10528-19	KF653665
996	*Longitarsus ordinatus*	GBCCH10531-19	KF653673
997	*Longitarsus ordinatus*	GBCCH10554-19	KF653703
998	*Longitarsus ordinatus*	GBCCH10558-19	KF653728
999	*Longitarsus ordinatus*	GBCCH10575-19	KF653748
1000	*Longitarsus ordinatus*	GBCCH10578-19	KF653751
1001	*Longitarsus ordinatus*	GBCCH10611-19	KF654626
1002	*Longitarsus ordinatus*	GBCCH10494-19	KF653483
1003	*Longitarsus ordinatus*	GBCCH10514-19	KF653595
1004	*Longitarsus ordinatus*	GBCCH10516-19	KF653597
1005	*Longitarsus ordinatus*	GBCCH10525-19	KF653661
1006	*Longitarsus ordinatus*	GBCCH10553-19	KF653702
1007	*Longitarsus ordinatus*	GBCCH10556-19	KF653726
1008	*Longitarsus ordinatus*	GBCCH10579-19	KF653752
1009	*Longitarsus ordinatus*	GBCCH10589-19	KF653834
1010	*Longitarsus ordinatus*	GBCCH10485-19	KF653366
1011	*Longitarsus ordinatus*	GBCCH10489-19	KF653379
1012	*Longitarsus ordinatus*	GBCCH10509-19	KF653590
1013	*Longitarsus ordinatus*	GBCCH10546-19	KF653695
1014	*Longitarsus ordinatus*	GBCCH10559-19	KF653729
1015	*Longitarsus ordinatus*	GBCCH10568-19	KF653739

1016	*Longitarsus ordinatus*	GBCCH10593-19	KF653875
1017	*Longitarsus ordinatus*	GBCCH10480-19	KF653360
1018	*Longitarsus ordinatus*	GBCCH10501-19	KF653490
1019	*Longitarsus ordinatus*	GBCCH10594-19	KF654217
1020	*Longitarsus ordinatus*	GBCCH10463-19	KF652760
1021	*Longitarsus ordinatus*	GBCCH10513-19	KF653594
1022	*Longitarsus ordinatus*	GBCCH10572-19	KF653744
1023	*Longitarsus ordinatus*	GBCCH10449-19	KF653730
1024	*Longitarsus ordinatus*	GBCCH10451-19	KF652569
1025	*Longitarsus ordinatus*	GBCCH10459-19	KF652570
1026	*Longitarsus ordinatus*	GBCCH10470-19	KF653130
1027	*Longitarsus ordinatus*	GBCCH10512-19	KF653593
1028	*Longitarsus ordinatus*	GBCCH10523-19	KF653606
1029	*Longitarsus ordinatus*	GBCCH10530-19	KF653672
1030	*Longitarsus ordinatus*	GBCCH10547-19	KF653696
1031	*Longitarsus ordinatus*	GBCCH10549-19	KF653698
1032	*Longitarsus ordinatus*	GBCCH10583-19	KF653756
1033	*Longitarsus ordinatus*	GBCCH10585-19	KF653823
1034	*Longitarsus ordinatus*	GBCCH10587-19	KF653825
1035	*Longitarsus ordinatus*	GBCCH10600-19	KF654551
1036	*Longitarsus ordinatus*	GBCCH10606-19	KF654575
1037	*Longitarsus ordinatus*	GBCCH10450-19	KF653325
1038	*Longitarsus ordinatus*	GBCCH10505-19	KF653581
1039	*Longitarsus ordinatus*	GBCCH10508-19	KF653589
1040	*Longitarsus ordinatus*	GBCCH10526-19	KF653662
1041	*Longitarsus ordinatus*	GBCCH10561-19	KF653732
1042	*Longitarsus ordinatus*	GBCCH10567-19	KF653738
1043	*Longitarsus ordinatus*	GBCCH10586-19	KF653824
1044	*Longitarsus ordinatus*	GBCCH10596-19	KF654531
1045	*Longitarsus ordinatus*	GBCCH10610-19	KF654625
1046	*Longitarsus ordinatus*	GBCCH10493-19	KF653482
1047	*Longitarsus ordinatus*	GBCCH10499-19	KF653488
1048	*Longitarsus ordinatus*	GBCCH10522-19	KF653604
1049	*Longitarsus ordinatus*	GBCCH10551-19	KF653700
1050	*Longitarsus ordinatus*	GBCCH10563-19	KF653734
1051	*Longitarsus ordinatus*	GBCCH10598-19	KF654533
1052	*Longitarsus ordinatus*	GBCCH10468-19	KF653128
1053	*Longitarsus ordinatus*	GBCCH10472-19	KF653158
1054	*Longitarsus ordinatus*	GBCCH10473-19	KF653159
1055	*Longitarsus ordinatus*	GBCCH10477-19	KF653357
1056	*Longitarsus ordinatus*	GBCCH10487-19	KF653368
1057	*Longitarsus ordinatus*	GBCCH10515-19	KF653596
1058	*Longitarsus ordinatus*	GBCCH10540-19	KF653689
1059	*Longitarsus ordinatus*	GBCCH10562-19	KF653733
1060	*Longitarsus ordinatus*	GBCCH10571-19	KF653743
1061	*Longitarsus ordinatus*	GBCCH10609-19	KF654594
1062	*Longitarsus ordinatus*	GBCCH10460-19	KF652571
1063	*Longitarsus ordinatus*	GBCCH10510-19	KF653591
1064	*Longitarsus ordinatus*	GBCCH10527-19	KF653663
1065	*Longitarsus ordinatus*	GBCCH10544-19	KF653693
1066	*Longitarsus ordinatus*	GBCCH10577-19	KF653750

1067	*Longitarsus ordinatus*	GBCCH10581-19	KF653754
1068	*Longitarsus ordinatus*	GBCCH10595-19	KF654218
1069	*Longitarsus ordinatus*	GBCCH10602-19	KF654563
1070	*Longitarsus ordinatus*	GBCCH10607-19	KF654592
1071	*Longitarsus ordinatus*	GBCCH10455-19	KF652598
1072	*Longitarsus ordinatus*	GBCCH10478-19	KF653358
1073	*Longitarsus ordinatus*	GBCCH10500-19	KF653489
1074	*Longitarsus ordinatus*	GBCCH10538-19	KF653687
1075	*Longitarsus ordinatus*	GBCCH10555-19	KF653704
1076	*Longitarsus ordinatus*	GBCCH10604-19	KF654565
1077	*Longitarsus ordinatus*	GBCCH10466-19	KF653126
1078	*Longitarsus ordinatus*	GBCCH10532-19	KF653680
1079	*Longitarsus parvulus*	COLNO009-09	
1080	*Longitarsus parvulus*	GBCCH10624-19	KF653502
1081	*Longitarsus parvulus*	GBCCH10630-19	KF653565
1082	*Longitarsus parvulus*	GBCCH10637-19	KF655419
1083	*Longitarsus parvulus*	GBCCH10633-19	HQ164815
1084	*Longitarsus parvulus*	COLFE176-12	KJ962080
1085	*Longitarsus parvulus*	GBCCH10635-19	HQ165575
1086	*Longitarsus parvulus*	GBCCH10622-19	KF653383
1087	*Longitarsus parvulus*	GBCCH10623-19	KF653384
1088	*Longitarsus parvulus*	GBCCH10617-19	KF652645
1089	*Longitarsus parvulus*	GBCCH10628-19	KF653526
1090	*Longitarsus parvulus*	GBCCH10632-19	DQ155875
1091	*Longitarsus parvulus*	GBCCH10638-19	KP763031
1092	*Longitarsus parvulus*	GBCOL897-12	KM443581
1093	*Longitarsus parvulus*	GBCCH10619-19	KF653257
1094	*Longitarsus parvulus*	FBCOA068-10	HQ563304
1095	*Longitarsus parvulus*	GBCCH10634-19	HQ165173
1096	*Longitarsus parvulus*	GBCCH10620-19	KF653348
1097	*Longitarsus parvulus*	FBCOG1222-12	KM441931
1098	*Longitarsus parvulus*	FBCOI948-12	KM446553
1099	*Longitarsus parvulus*	GBCCH10625-19	KF653522
1100	*Longitarsus parvulus*	GBCCH10631-19	KF653720
1101	*Longitarsus parvulus*	GBCCH10618-19	KF652456
1102	*Longitarsus parvulus*	GBCCH10639-19	KX943391
1103	*Longitarsus parvulus*	GCOL5316-16	KU914918
1104	*Longitarsus parvulus*	GBCCH10627-19	KF653525
1105	*Longitarsus parvulus*	FBCOJ429-12	KM451659
1106	*Longitarsus parvulus*	GBCCH10626-19	KF653524
1107	*Longitarsus parvulus*	GBCCH10636-19	KF655418
1108	*Longitarsus parvulus*	GBCCH10621-19	KF653362
1109	*Longitarsus parvulus*	GBCCH10629-19	KF653528
1110	*Longitarsus parvulus*	GBCCH10615-19	KF653527
1111	*Longitarsus parvulus*	GBCCH10616-19	KF653523
1112	*Longitarsus pellucidus*	FBCOD392-11	
1113	*Longitarsus pellucidus*	AGAKN759-17	MG054415
1114	*Longitarsus pellucidus*	AGAKO539-17	MG054895
1115	*Longitarsus pellucidus*	SMTPI6695-14	KR484411
1116	*Longitarsus pellucidus*	AGAKO538-17	MG059377
1117	*Longitarsus pellucidus*	AGAKO540-17	MG055065

1118	*Longitarsus pellucidus*	AGAKM228-17	MG056145
1119	*Longitarsus pellucidus*	AGAKL2329-17	MG057469
1120	*Longitarsus pellucidus*	AGAKT2416-17	MG056417
1121	*Longitarsus pellucidus*	SMTPD3340-13	KR487382
1122	*Longitarsus pellucidus*	SMTPD3355-13	KR488383
1123	*Longitarsus pellucidus*	GCOL2582-16	KU914889
1124	*Longitarsus pellucidus*	GCOL2416-16	KU908543
1125	*Longitarsus pellucidus*	AGAKN768-17	MG060779
1126	*Longitarsus pellucidus*	AGAKO533-17	MG053748
1127	*Longitarsus pellucidus*	CRHID586-15	MG055292
1128	*Longitarsus pellucidus*	SMTPD3344-13	KR480207
1129	*Longitarsus pellucidus*	AGAKO541-17	MG057935
1130	*Longitarsus pellucidus*	GCOL2739-16	KU906283
1131	*Longitarsus pinguis*	MEDLB566-12	MH323196
1132	*Longitarsus pinguis*		MK893909
1133	*Longitarsus pratensis*	OPPEI2993-17	
1134	*Longitarsus pratensis*	OPPEI3040-17	
1135	*Longitarsus pratensis*	OPPEI3455-17	
1136	*Longitarsus pratensis*	OPPEI2975-17	
1137	*Longitarsus pratensis*	BARSJ441-16	
1138	*Longitarsus pratensis*	OPPEI3448-17	
1139	*Longitarsus pratensis*	OPPEI3453-17	
1140	*Longitarsus pratensis*	OPPEI3463-17	
1141	*Longitarsus pratensis*	OPPEI3036-17	
1142	*Longitarsus pratensis*	OPPEI3022-17	
1143	*Longitarsus pratensis*	OPPEI3018-17	
1144	*Longitarsus pratensis*	BARSB267-16	
1145	*Longitarsus pratensis*	OPPEI3021-17	
1146	*Longitarsus pratensis*	OPPEI2997-17	
1147	*Longitarsus pratensis*	OPPEI3032-17	
1148	*Longitarsus pratensis*	BARSG539-16	
1149	*Longitarsus pratensis*	OPPEI3023-17	
1150	*Longitarsus pratensis*	OPPEI3027-17	
1151	*Longitarsus pratensis*	OPPEI3452-17	
1152	*Longitarsus pratensis*	OPPEI3016-17	
1153	*Longitarsus pratensis*	OPPEI3034-17	
1154	*Longitarsus pratensis*	OPPEI2996-17	
1155	*Longitarsus pratensis*	GBCCH10644-19	KP763070
1156	*Longitarsus pratensis*	PHCOL119-11	KR485404
1157	*Longitarsus pratensis*	SMTPO522-15	MG056813
1158	*Longitarsus pratensis*	SMTPO701-15	MG061708
1159	*Longitarsus pratensis*	SMTPR2410-16	MG057598
1160	*Longitarsus pratensis*	JSCOL377-11	KR480761
1161	*Longitarsus pratensis*	MBIOH599-13	KR490284
1162	*Longitarsus pratensis*	SMTPO6706-15	MG060737
1163	*Longitarsus pratensis*	SMTPR3662-16	MG054080
1164	*Longitarsus pratensis*	PHSEP1965-11	KR490324
1165	*Longitarsus pratensis*	SMTPD3342-13	KR480557
1166	*Longitarsus pratensis*	SMTPI3230-14	KR485032
1167	*Longitarsus pratensis*	SMTPO4866-15	MG058363
1168	*Longitarsus pratensis*	SMTPO6710-15	MG058392

1169	*Longitarsus pratensis*	SMTPR1332-16	MG062332
1170	*Longitarsus pratensis*	CRHIG033-16	MG061761
1171	*Longitarsus pratensis*	FBCOB641-10	HQ954183
1172	*Longitarsus pratensis*	PHCOL108-11	KR484810
1173	*Longitarsus pratensis*	SMTPD3351-13	KR482604
1174	*Longitarsus pratensis*	SMTPR240-16	MG055279
1175	*Longitarsus pratensis*	GBCCH10640-19	KF654702
1176	*Longitarsus pratensis*	SMTPR2432-16	MG062077
1177	*Longitarsus pratensis*	SMTPR3653-16	MG058152
1178	*Longitarsus pratensis*	AGAKT2414-17	MG058354
1179	*Longitarsus pratensis*	CRHIB820-15	MG060053
1180	*Longitarsus pratensis*	CRHIJ008-16	MG059462
1181	*Longitarsus pratensis*	CRHIJ579-16	MG055637
1182	*Longitarsus pratensis*	GBCCH10643-19	KF656428
1183	*Longitarsus pratensis*	SMTPB13446-13	KR480124
1184	*Longitarsus pratensis*	SMTPO5279-15	MG053928
1185	*Longitarsus pratensis*	SMTPO711-15	MG054759
1186	*Longitarsus pratensis*	SMTPR6733-16	MG059578
1187	*Longitarsus pratensis*	SMTPR7165-16	MG061730
1188	*Longitarsus pratensis*	SMTPR7349-16	MG056655
1189	*Longitarsus pratensis*	CRHIB1034-16	MG059547
1190	*Longitarsus pratensis*	HESEP875-12	KR480834
1191	*Longitarsus pratensis*	SMTPO6704-15	MG055432
1192	*Longitarsus pratensis*	CRHIB825-15	MG056611
1193	*Longitarsus pratensis*	SMTPI9795-14	KR487634
1194	*Longitarsus pratensis*	SMTPO712-15	MG054361
1195	*Longitarsus pratensis*	SMTPR3836-16	MG054324
1196	*Longitarsus pratensis*	SMTPR3845-16	MG056183
1197	*Longitarsus pratensis*	SMTPR7361-16	MG055430
1198	*Longitarsus pratensis*	SMTPR7370-16	MG058777
1199	*Longitarsus pratensis*	CRHIJ836-16	MG055614
1200	*Longitarsus pratensis*	JSCOL117-11	KR490475
1201	*Longitarsus pratensis*	PAOCT002-12	KR480364
1202	*Longitarsus pratensis*	SMTPB1054-13	KR489964
1203	*Longitarsus pratensis*	SMTPJ714-14	KR491284
1204	*Longitarsus pratensis*	SMTPL148-15	MG054537
1205	*Longitarsus pratensis*	SMTPO6709-15	MG060660
1206	*Longitarsus pratensis*	SMTPO695-15	MG058030
1207	*Longitarsus pratensis*	SMTPR4656-16	MG060741
1208	*Longitarsus pratensis*	CRHIB1468-16	MG054921
1209	*Longitarsus pratensis*	CRHIJ511-16	MG053596
1210	*Longitarsus pratensis*	CRHIJ562-16	MG059163
1211	*Longitarsus pratensis*	CRHIJ837-16	MG053549
1212	*Longitarsus pratensis*	HEOCT1044-12	KR488814
1213	*Longitarsus pratensis*	SMTPB1048-13	KR480130
1214	*Longitarsus pratensis*	SMTPD495-13	KR488280
1215	*Longitarsus pratensis*	SMTPI1307-14	KR489279
1216	*Longitarsus pratensis*	SMTPO4858-15	MG060285
1217	*Longitarsus pratensis*	SMTPR235-16	MG056091
1218	*Longitarsus pratensis*	COLON018-10	HQ942791
1219	*Longitarsus pratensis*	CRHIB371-15	MG055394

1220	*Longitarsus pratensis*	CRHIB372-15	MG059339
1221	*Longitarsus pratensis*	GCOL10087-16	KU919592
1222	*Longitarsus pratensis*	GCOL5255-16	KU907078
1223	*Longitarsus pratensis*	SMTPO698-15	MG060904
1224	*Longitarsus pratensis*	SMTPR3107-16	MG058633
1225	*Longitarsus pratensis*	SMTPR7347-16	MG055493
1226	*Longitarsus pratensis*	SMTPR7363-16	MG058266
1227	*Longitarsus pratensis*	CNGBN2842-14	KR126393
1228	*Longitarsus pratensis*	HESEP882-12	KR482097
1229	*Longitarsus pratensis*	PHCOL092-11	KR488608
1230	*Longitarsus pratensis*	PHNOV523-11	KR486228
1231	*Longitarsus pratensis*	PHOCT617-11	KR482841
1232	*Longitarsus pratensis*	PHSEP1976-11	KR484199
1233	*Longitarsus pratensis*	SMTPD3350-13	KR489500
1234	*Longitarsus pratensis*	CNGBA992-13	KR122926
1235	*Longitarsus pratensis*	CRHIB370-15	MG053800
1236	*Longitarsus pratensis*	CRHIJ835-16	MG056581
1237	*Longitarsus pratensis*	HEOCT1042-12	KR487716
1238	*Longitarsus pratensis*	JSCOL363-11	KR488474
1239	*Longitarsus pratensis*	PASEP001-12	KR481305
1240	*Longitarsus pratensis*	PHCOL097-11	KR484826
1241	*Longitarsus pratensis*	SMTPR3839-16	MG057195
1242	*Longitarsus pratensis*	SMTPR7368-16	MG053855
1243	*Longitarsus pratensis*	ASCMT297-11	MG062601
1244	*Longitarsus pratensis*	CNGBG1601-14	KR125821
1245	*Longitarsus pratensis*	CNGBO1101-14	KR130105
1246	*Longitarsus pratensis*	GCOL2729-16	KU907084
1247	*Longitarsus pratensis*	HESEP880-12	KR483422
1248	*Longitarsus pratensis*	SMTPI9312-14	KR489822
1249	*Longitarsus pratensis*	CRHIG454-16	MG057941
1250	*Longitarsus pratensis*	GBCCH10645-19	KX943360
1251	*Longitarsus pratensis*	SMTPB1058-13	KR485678
1252	*Longitarsus pratensis*	SMTPO697-15	MG059994
1253	*Longitarsus pratensis*	SMTPP1120-15	MG054632
1254	*Longitarsus pratensis*	SMTPR10545-16	MG062333
1255	*Longitarsus pratensis*	COLON034-10	JF888941
1256	*Longitarsus pratensis*	CRHIB367-15	MG053886
1257	*Longitarsus pratensis*	CRHIG261-16	MG054540
1258	*Longitarsus pratensis*	GBCCH10641-19	KF655025
1259	*Longitarsus pratensis*	SMTPI1452-14	KR481516
1260	*Longitarsus pratensis*	SMTPO110-15	MG055102
1261	*Longitarsus pratensis*	CRHIG034-16	MG055940
1262	*Longitarsus pratensis*	GBCCH10642-19	KF655098
1263	*Longitarsus pratensis*	HEOCT1046-12	KR485790
1264	*Longitarsus pratensis*	SMTPI1309-14	KR489548
1265	*Longitarsus pratensis*	SMTPR7341-16	MG059916
1266	*Longitarsus pratensis*	CNGBG1618-14	KR119941
1267	*Longitarsus pratensis*	SMTPR2275-16	MG056442
1268	*Longitarsus pratensis*	SMTPR5097-16	MG055624
1269	*Longitarsus pratensis*	SMTPR7356-16	MG057787
1270	*Longitarsus pratensis*	SMTPR7366-16	MG057927

1271	*Longitarsus pratensis*	JSCOL107-11	KR487457
1272	*Longitarsus pratensis*	SMTPJ315-14	KR488627
1273	*Longitarsus pratensis*	SMTPR7364-16	MG057346
1274	*Longitarsus pratensis*	BARSF378-16	MG053727
1275	*Longitarsus pratensis*	SMTPB1060-13	KR490677
1276	*Longitarsus pratensis*	SMTPB13950-13	KR486740
1277	*Longitarsus pratensis*	SMTPF8184-14	KR486529
1278	*Longitarsus pratensis*	SMTPR2271-16	MG055051
1279	*Longitarsus pratensis*	SMTPR357-16	MG061457
1280	*Longitarsus pratensis*	SMTPR4799-16	MG059791
1281	*Longitarsus pratensis*	CNGBK1554-14	KR122849
1282	*Longitarsus pratensis*	CRHIJ495-16	MG061126
1283	*Longitarsus pratensis*	GCOL2588-16	KU918331
1284	*Longitarsus pratensis*	HESEP881-12	KR486278
1285	*Longitarsus pratensis*	SMTPD3354-13	KR491188
1286	*Longitarsus pratensis*	SMTPD831-13	KR480203
1287	*Longitarsus pratensis*	SMTPL808-15	MG062432
1288	*Longitarsus pratensis*	SMTPS800-16	MG053745
1289	*Longitarsus pratensis*	BARSE703-16	MG057311
1290	*Longitarsus pratensis*	CRHIB369-15	MG061041
1291	*Longitarsus pratensis*	CRHIE935-16	MG056138
1292	*Longitarsus pratensis*	SMTPR246-16	MG056688
1293	*Longitarsus pratensis*	SMTPR3657-16	MG059745
1294	*Longitarsus pratensis*	SMTPO699-15	MG055711
1295	*Longitarsus pulmonariae*	GCOL2488-16	KU908294
1296	*Longitarsus pulmonariae*	GCOL5242-16	KU909407
1297	*Longitarsus pulmonariae*	GCOL2471-16	KU916503
1298	*Longitarsus pulmonariae*	GCOL2680-16	KU917685
1299	*Longitarsus pulmonariae*	GCOL5240-16	KU907544
1300	*Longitarsus pulmonariae*	GCOL2504-16	KU907407
1301	*Longitarsus pulmonariae*	GCOL5226-16	KU907377
1302	*Longitarsus quadriguttatus*	AMRSG017-16	
1303	*Longitarsus quadriguttatus*	GCOL2418-16	KU908955
1304	*Longitarsus quadriguttatus*	FBCOB760-10	HQ954268
1305	*Longitarsus quadriguttatus*	GCOL2433-16	KU909023
1306	*Longitarsus quadriguttatus*	FBCON028-13	KM446524
1307	*Longitarsus quadriguttatus*	FBCOB759-10	HQ954267
1308	*Longitarsus rectilineatus*		MK893910
1309	*Longitarsus refugiensis*	MEDLB476-12	MH323199
1310	*Longitarsus refugiensis*	MEDLB478-12	MH323197
1311	*Longitarsus refugiensis*	MEDLB373-12	MH323198
1312	*Longitarsus refugiensis*	MEDLB639-12	MH323200
1313	*Longitarsus reichei*	GCOL2701-16	KU917139
1314	*Longitarsus reichei*	GCOL2485-16	KU918316
1315	*Longitarsus reichei*	GBCCH10646-19	HQ164727
1316	*Longitarsus reichei*	GCOL5229-16	KU907319
1317	*Longitarsus reichei*	GCOL2484-16	KU914666
1318	*Longitarsus reichei*		KJ963049
1319	*Longitarsus rubellus*	GBCOU2795-13	KM450669
1320	*Longitarsus rubiginosus*	COLFD821-12	KJ965978
1321	*Longitarsus rubiginosus*	FBCOD391-11	KM448549

1322	*Longitarsus rubiginosus*	RBINA2651-13	KR481842
1323	*Longitarsus rubiginosus*	GBCOB715-12	KM452579
1324	*Longitarsus rubiginosus*	GCOL2707-16	KU912815
1325	*Longitarsus rubiginosus*	GCOL5247-16	KU919152
1326	*Longitarsus rubiginosus*	FBCOC933-10	KM439133
1327	*Longitarsus rubiginosus*	GCOL2733-16	KU910130
1328	*Longitarsus rubiginosus*	GCOL2710-16	KU912038
1329	*Longitarsus rubiginosus*	GBCOB714-12	KM448522
1330	*Longitarsus rubiginosus*	SMTPP1116-15	MG059726
1331	*Longitarsus rubiginosus*	RBINA2251-13	KR484997
1332	*Longitarsus rubiginosus*	FBCOD390-11	KM445763
1333	*Longitarsus rubiginosus*	SMTPP1129-15	MG054058
1334	*Longitarsus rubiginosus*	RBINA2343-13	KR485140
1335	*Longitarsus rubiginosus*	GCOL5248-16	KU906379
1336	*Longitarsus rubiginosus*	JSAUG1576-11	KR481901
1337	*Longitarsus rubiginosus*	RBINA2252-13	KR491441
1338	*Longitarsus rutilus*	GBCCH10647-19	KF653536
1339	*Longitarsus rutilus*	GBCCH10653-19	KF653123
1340	*Longitarsus rutilus*	GBCCH10668-19	KX943491
1341	*Longitarsus rutilus*	GBCCH10656-19	KF653429
1342	*Longitarsus rutilus*	GBCCH10658-19	KF653431
1343	*Longitarsus rutilus*	GBCCH10662-19	KF653519
1344	*Longitarsus rutilus*	GBCCH10648-19	KF652682
1345	*Longitarsus rutilus*	GBCCH10663-19	HQ164589
1346	*Longitarsus rutilus*	GBCCH10666-19	KF654885
1347	*Longitarsus rutilus*	GBCCH10660-19	KF653446
1348	*Longitarsus rutilus*	GBCCH10654-19	KF653156
1349	*Longitarsus rutilus*	GBCCH10661-19	KF653447
1350	*Longitarsus rutilus*	GBCCH10664-19	KF654794
1351	*Longitarsus rutilus*	GBCCH10665-19	KF654849
1352	*Longitarsus rutilus*	GBCCH10649-19	KF652815
1353	*Longitarsus rutilus*	GBCCH10655-19	KF653417
1354	*Longitarsus rutilus*	GBCCH10667-19	KP763059
1355	*Longitarsus rutilus*	GBCCH10657-19	KF653430
1356	*Longitarsus rutilus*	GBCCH10650-19	KF652839
1357	*Longitarsus rutilus*	GBCCH10651-19	KF652855
1358	*Longitarsus rutilus*	GBCCH10652-19	KF653122
1359	*Longitarsus rutilus*	GBCCH10659-19	KF653445
1360	*Longitarsus salviae*	GBCCH10669-19	KF653386
1361	*Longitarsus salviae*	GCOL2586-16	KU912486
1362	*Longitarsus salviae*	GCOL2611-16	KU907298
1363	*Longitarsus salviae*	GCOL2638-16	KU916622
1364	*Longitarsus salviae*	GBCCH10670-19	KP763015
1365	*Longitarsus salviae*	FBCOH626-12	KM444365
1366	*Longitarsus saulicus*		MK893911
1367	*Longitarsus scutellaris*	FBCOP298-13	KM451412
1368	*Longitarsus scutellaris*	FBCOB604-10	HQ954159
1369	*Longitarsus scutellaris*	JSCOL389-11	MG058553
1370	*Longitarsus scutellaris*	FBCOB603-10	HQ954158
1371	*Longitarsus scutellaris*		KR126393
1372	*Longitarsus scutellaris*		KR125821

1373	*Longitarsus scutellaris*	KR122926
1374	*Longitarsus scutellaris*	KR122849
1375	*Longitarsus scutellaris*	KR130105
1376	*Longitarsus scutellaris*	KR119941
1377	*Longitarsus scutellaris*	KR487634
1378	*Longitarsus scutellaris*	KR487457
1379	*Longitarsus scutellaris*	KR486740
1380	*Longitarsus scutellaris*	KR486529
1381	*Longitarsus scutellaris*	KR486278
1382	*Longitarsus scutellaris*	KR486228
1383	*Longitarsus scutellaris*	KR485790
1384	*Longitarsus scutellaris*	KR485678
1385	*Longitarsus scutellaris*	KR485404
1386	*Longitarsus scutellaris*	KR485032
1387	*Longitarsus scutellaris*	KR484826
1388	*Longitarsus scutellaris*	KR484810
1389	*Longitarsus scutellaris*	KR484199
1390	*Longitarsus scutellaris*	KR483422
1391	*Longitarsus scutellaris*	KR482841
1392	*Longitarsus scutellaris*	KR482604
1393	*Longitarsus scutellaris*	KR482097
1394	*Longitarsus scutellaris*	KR481516
1395	*Longitarsus scutellaris*	KR481305
1396	*Longitarsus scutellaris*	KR480834
1397	*Longitarsus scutellaris*	KR480761
1398	*Longitarsus scutellaris*	KR480557
1399	*Longitarsus scutellaris*	KR480364
1400	*Longitarsus scutellaris*	KR480203
1401	*Longitarsus scutellaris*	KR480130
1402	*Longitarsus scutellaris*	KR480124
1403	*Longitarsus scutellaris*	KR491284
1404	*Longitarsus scutellaris*	KR491188
1405	*Longitarsus scutellaris*	KR490677
1406	*Longitarsus scutellaris*	KR490475
1407	*Longitarsus scutellaris*	KR490324
1408	*Longitarsus scutellaris*	KR489964
1409	*Longitarsus scutellaris*	KR489548
1410	*Longitarsus scutellaris*	KR489500
1411	*Longitarsus scutellaris*	KR489279
1412	*Longitarsus scutellaris*	KR488814
1413	*Longitarsus scutellaris*	KR488627
1414	*Longitarsus scutellaris*	KR488608
1415	*Longitarsus scutellaris*	KR488474
1416	*Longitarsus scutellaris*	KR488280
1417	*Longitarsus scutellaris*	KR487716
1418	*Longitarsus scutellaris*	MG062601
1419	*Longitarsus scutellaris*	MG062432
1420	*Longitarsus scutellaris*	MG061708
1421	*Longitarsus scutellaris*	MG060904
1422	*Longitarsus scutellaris*	MG060737
1423	*Longitarsus scutellaris*	MG060660

1424	*Longitarsus scutellaris*		MG060285
1425	*Longitarsus scutellaris*		MG059994
1426	*Longitarsus scutellaris*		MG058392
1427	*Longitarsus scutellaris*		MG058363
1428	*Longitarsus scutellaris*		MG058030
1429	*Longitarsus scutellaris*		MG056813
1430	*Longitarsus scutellaris*		MG055711
1431	*Longitarsus scutellaris*		MG055637
1432	*Longitarsus scutellaris*		MG055432
1433	*Longitarsus scutellaris*		MG055102
1434	*Longitarsus scutellaris*		MG054759
1435	*Longitarsus scutellaris*		MG054632
1436	*Longitarsus scutellaris*		MG054537
1437	*Longitarsus scutellaris*		MG054361
1438	*Longitarsus scutellaris*		MG053928
1439	*Longitarsus sencieri*	GBCCH10671-19	KP763014
1440	*Longitarsus springeri*		MK893912
1441	*Longitarsus strigicollis*	GCOL5239-16	KU914767
1442	*Longitarsus strigicollis*	GCOL2607-16	KU907304
1443	*Longitarsus strigicollis*	GBCCH10673-19	KF653533
1444	*Longitarsus strigicollis*	GBCCH10675-19	KF653521
1445	*Longitarsus strigicollis*	GBCCH10674-19	KF653520
1446	*Longitarsus succineus*	AMRSG007-16	
1447	*Longitarsus succineus*	COLFD828-12	KJ962137
1448	*Longitarsus succineus*	COLFF933-13	KJ967090
1449	*Longitarsus succineus*	GCOL1809-16	KU918059
1450	*Longitarsus succineus*	COLFE199-12	KJ962131
1451	*Longitarsus succineus*	GCOL2481-16	KU917423
1452	*Longitarsus succineus*	MEDLB746-12	MH323205
1453	*Longitarsus succineus*	COLFE1489-13	KJ965295
1454	*Longitarsus succineus*	GBCCH10676-19	HQ164656
1455	*Longitarsus succineus*	MEDLB747-12	MH323206
1456	*Longitarsus succineus*	MEDLB745-12	MH323207
1457	*Longitarsus succineus*	GCOL2437-16	KU912727
1458	*Longitarsus suspectus*	SSROB2951-14	KR479974
1459	*Longitarsus suspectus*	SSROB1000-14	KR487316
1460	*Longitarsus suspectus*	RBINA2253-13	KR483697
1461	*Longitarsus suturellus*	GCOL11743-16	KU919071
1462	*Longitarsus suturellus*	GCOL8188-16	KU913427
1463	*Longitarsus suturellus*	COLFC442-12	KJ965495
1464	*Longitarsus suturellus*	GCOL8577-16	KU912271
1465	*Longitarsus suturellus*	GCOL8189-16	KU915636
1466	*Longitarsus suturellus*	GCOL8578-16	KU909995
1467	*Longitarsus suturellus*	GCOL11742-16	KU915687
1468	*Longitarsus symphyti*	GCOL2429-16	KU908859
1469	*Longitarsus symphyti*	GCOL2570-16	KU909558
1470	*Longitarsus symphyti*	GCOL2681-16	KU917412
1471	*Longitarsus symphyti*	GCOL2571-16	KU907585
1472	*Longitarsus symphyti*	GCOL2696-16	KU917194
1473	*Longitarsus symphyti*	GCOL2430-16	KU912680
1474	*Longitarsus tabidus*	FBCOK094-13	KM451576

1475	*Longitarsus tabidus*	GBCCH10677-19	KF652996
1476	*Longitarsus tabidus*	FBCOB189-10	HQ953850
1477	*Longitarsus tabidus*	GBCCH10681-19	KF655389
1478	*Longitarsus tabidus*	GCOL12494-16	KU914608
1479	*Longitarsus tabidus*	GCOL2575-16	KU909321
1480	*Longitarsus tabidus*	GBCCH10680-19	HQ164914
1481	*Longitarsus tabidus*	GCOL2346-16	KU910013
1482	*Longitarsus tabidus*	FBCON006-13	KM441889
1483	*Longitarsus tabidus*	GBCCH10683-19	KP763037
1484	*Longitarsus tabidus*	FBCON007-13	KM439382
1485	*Longitarsus tabidus*	GBCOG673-13	KM440725
1486	*Longitarsus tabidus*	GBCOU3558-13	KM446886
1487	*Longitarsus tabidus*	GBCCH10682-19	KP306813
1488	*Longitarsus tabidus*	GBCOU3592-13	KM441772
1489	*Longitarsus tabidus*	GCOL12482-16	KU918450
1490	*Longitarsus tabidus*	GBCCH10678-19	KF653901
1491	*Longitarsus tabidus*	GBCCH10684-19	KX943424
1492	*Longitarsus tabidus*	GCOL7777-16	KU918996
1493	*Longitarsus tabidus*	COLFD940-12	KJ962415
1494	*Longitarsus tabidus*	GBCCH10679-19	KF654096
1495	*Longitarsus tabidus*	GBCOU3593-13	KM449602
1496	*Longitarsus tabidus*	GCOL5312-16	KU917773
1497	*Longitarsus tabidus*	GCOL7778-16	KU918442
1498	*Longitarsus testaceus*		KR128307
1499	*Longitarsus testaceus*		KR128206
1500	*Longitarsus testaceus*		KR127953
1501	*Longitarsus testaceus*		KR127636
1502	*Longitarsus testaceus*		KR126756
1503	*Longitarsus testaceus*		KR126712
1504	*Longitarsus testaceus*		KR126242
1505	*Longitarsus testaceus*		KR125515
1506	*Longitarsus testaceus*		KR124936
1507	*Longitarsus testaceus*		KR124289
1508	*Longitarsus testaceus*		KR123803
1509	*Longitarsus testaceus*		KR123652
1510	*Longitarsus testaceus*		KR123264
1511	*Longitarsus testaceus*		KR123152
1512	*Longitarsus testaceus*		KR122822
1513	*Longitarsus testaceus*		KR122373
1514	*Longitarsus testaceus*		KR122263
1515	*Longitarsus testaceus*		KR131101
1516	*Longitarsus testaceus*		KR130867
1517	*Longitarsus testaceus*		KR130528
1518	*Longitarsus testaceus*		KR130297
1519	*Longitarsus testaceus*		KR128969
1520	*Longitarsus testaceus*		KR128939
1521	*Longitarsus testaceus*		KR128910
1522	*Longitarsus testaceus*		KR128362
1523	*Longitarsus testaceus*		KR128356
1524	*Longitarsus testaceus*		KR121993
1525	*Longitarsus testaceus*		KR120663

1526	*Longitarsus testaceus*		KR120371
1527	*Longitarsus testaceus*		KR119567
1528	*Longitarsus testaceus*		KR119207
1529	*Longitarsus testaceus*		KR119120
1530	*Longitarsus testaceus*		KR484403
1531	*Longitarsus testaceus*		KR484048
1532	*Longitarsus testaceus*		KR481430
1533	*Longitarsus testaceus*		KR481184
1534	*Longitarsus testaceus*		KR490736
1535	*Longitarsus testaceus*		KR489660
1536	*Longitarsus vilis*	GBCCH10685-19	KF653346
1537	*Longitarsus zangherii*		MK893913
