# Supplementary material for: Sharpening the DNA barcoding tool through a posteriori taxonomic validation: The case of Longitarsus flea beetles (Coleoptera: Chrysomelidae)
Source: PLoS One. 2020 May 21;15(5):e0233573. doi: 10.1371/journal.pone.0233573 (PMC7241800; doi:10.1371/journal.pone.0233573)
Supplement: S2 Table — For each specimen are reported voucher info, place and date of collection, coordinates, collectors, BOLD and GenBank accession numbers. All specimens have been identified by Maurizio Biondi (University of L’Aquila). (PDF) [file pone.0233573.s002.pdf]

Supplementary Table S2. List of specimens sequenced in this study. For each specimen are reported voucher info, place and date of collection, coordinates, collectors, BOLD and GenBank accession numbers. All specimens have been identified by Maurizio Biondi (University of L'Aquila).

Species	Voucher	Country	Coordinate (Lat Lon)		Altitude (m a.s.l.)	Collector	BOLD ID	GenBank ID
Longitarsus aenicollis	db46	Italy, Abruzzo (TE), Prati di Tivo	42.51	13.55	1344m	M. Biondi & P. D'Alessandro	BARLG110-20	MK893902
Longitarsus aenicollis	9B	Italy, Abruzzo (AQ), Campo Imperatore	42.43	13.61	1669m	M. Biondi & P. D'Alessandro	BARLG084-20	MT372333
Longitarsus aenicollis	9D	Italy, Abruzzo (AQ), Campo Imperatore	42.43	13.61	1669m	M. Biondi & P. D'Alessandro	BARLG092-20	MT372331
Longitarsus aenicollis	9A	Italy, Abruzzo (AQ), Campo Imperatore	42.43	13.61	1669m	M. Biondi & P. D'Alessandro	BARLG093-20	MT372332
Longitarsus albineus	10D	Italy, Puglia (LE), Ruffano	40.00	18.25	106m	L. De Vitis	BARLG088-20	MT372334
Longitarsus albineus	10C	Italy, Puglia (LE), Ruffano	40.00	18.25	106m	L. De Vitis	BARLG089-20	MT372335
Longitarsus albineus	10B	Italy, Puglia (LE), Ruffano	40.00	18.25	106m	L. De Vitis	BARLG090-20	MT372337
Longitarsus albineus	10A	Italy, Puglia (LE), Ruffano	40.00	18.25	106m	L. De Vitis	BARLG091-20	MT372336
Longitarsus anchusae	14D	Italy, Abruzzo (AQ), 2.6 km SW of Aprati	42.54	13.45	862m	M. Biondi & P. D'Alessandro	BARLG078-20	MT372339
Longitarsus anchusae	14A	Italy, Abruzzo (AQ), 2.6 km SW of Aprati	42.54	13.45	862m	M. Biondi & P. D'Alessandro	BARLG079-20	MT372338
Longitarsus anchusae	db19a	Italy, Abruzzo (AQ), 2.6 km SW of Aprati	42.54	13.45	862m	M. Biondi & P. D'Alessandro	BARLG099-20	MT372341
Longitarsus anchusae	db19b	Italy, Abruzzo (AQ), 2.6 km SW of Aprati	42.54	13.45	862m	M. Biondi & P. D'Alessandro	BARLG114-20	MT372340
Longitarsus ballotae	11A	Italy, Abruzzo (TE), Faraone Antico	42.80	13.68	317m	M. Biondi	BARLG083-20	MT372343
Longitarsus ballotae	11B	Italy, Abruzzo (TE), Faraone Antico	42.80	13.68	317m	M. Biondi	BARLG087-20	MT372342
Longitarsus ballotae	db28	Italy, Abruzzo (TE), Faraone Antico	42.80	13.68	317m	M. Biondi	BARLG102-20	MT372344
Longitarsus candidulus	35D	Italy, Lazio (LT), Lungomare di Sabaudia	41.25	13.04	9m	C. Mantoni, L. De Vitis, M. Iannella, G. Console	BARLG007-20	MT372345
Longitarsus candidulus	35B	Italy, Lazio (LT), Lungomare di Sabaudia	41.25	13.04	9m	C. Mantoni, L. De Vitis, M. Iannella, G. Console	BARLG008-20	MT372346
Longitarsus candidulus	35A	Italy, Lazio (LT), Lungomare di Sabaudia	41.25	13.04	9m	C. Mantoni, L. De Vitis, M. Iannella, G. Console	BARLG009-20	MT372347
Longitarsus cerinthes	db1	Portugal, Madeira Island, 1.5 km SE of Nogueira	32.64	-16.83	22m	M. Biondi	BARLG095-20	MT372349
Longitarsus cerinthes	db2	Portugal, Madeira Island, 1 km SE of Nogueira	32.64	-16.83	107m	M. Biondi	BARLG096-20	MT372348
Longitarsus corynthius metallescens	26D	Italy, Abruzzo (AQ), Area Archeologica Amiternum	42.40	13.31	669m	E. Berrilli, P. D'Alessandro, L. De Vitis, D. Salvi	BARLG045-20	MT372351
Longitarsus corynthius metallescens	26C	Italy, Abruzzo (AQ), Area Archeologica Amiternum	42.40	13.31	669m	E. Berrilli, P. D'Alessandro, L. De Vitis, D. Salvi	BARLG046-20	MT372352
Longitarsus corynthius metallescens	26B	Italy, Abruzzo (AQ), Area Archeologica Amiternum	42.40	13.31	669m	E. Berrilli, P. D'Alessandro, L. De Vitis, D. Salvi	BARLG047-20	MT372350
Longitarsus corynthius metallescens	26A	Italy, Abruzzo (AQ), Area Archeologica Amiternum	42.40	13.31	669m	E. Berrilli, P. D'Alessandro, L. De Vitis, D. Salvi	BARLG048-20	MT372353
Longitarsus echii	29D	Italy, Abruzzo (AQ), Area Archeologica Amiternum	42.40	13.31	669m	E. Berrilli, P. D'Alessandro, L. De Vitis, D. Salvi	BARLG035-20	MT372356
Longitarsus echii	29C	Italy, Abruzzo (AQ), Area Archeologica Amiternum	42.40	13.31	669m	E. Berrilli, P. D'Alessandro, L. De Vitis, D. Salvi	BARLG036-20	MT372354
Longitarsus echii	29B	Italy, Abruzzo (AQ), Area Archeologica Amiternum	42.40	13.31	669m	E. Berrilli, P. D'Alessandro, L. De Vitis, D. Salvi	BARLG037-20	MT372355
Longitarsus echii	29A	Italy, Abruzzo (AQ), Area Archeologica Amiternum	42.40	13.31	669m	E. Berrilli, P. D'Alessandro, L. De Vitis, D. Salvi	BARLG038-20	MT372357
Longitarsus exoletus	33C	Italy, Abruzzo (AQ), near Coppito	42.37	13.35	650m	P. D'Alessandro	BARLG020-20	MT372358
Longitarsus exoletus	33B	Italy, Abruzzo (AQ), near Coppito	42.37	13.35	650m	P. D'Alessandro	BARLG021-20	MT372360
Longitarsus exoletus	33A	Italy, Abruzzo (AQ), near Coppito	42.37	13.35	650m	P. D'Alessandro	BARLG022-20	MT372359
Longitarsus exsoletus	db18	Italy, Abruzzo (AQ), near Coppito	42.37	13.35	650m	P. D'Alessandro	BARLG112-20	MT372361
Longitarsus foudrasi	19C	Italy, Puglia (LE), Cardigliano, Specchia	39.94	18.27	160m	L. De Vitis	BARLG067-20	MT372362
Longitarsus foudrasi	19B	Italy, Puglia (LE), Cardigliano, Specchia	39.94	18.27	160m	L. De Vitis	BARLG068-20	MT372363
Longitarsus foudrasi	19A	Italy, Puglia (LE), Cardigliano, Specchia	39.94	18.27	160m	L. De Vitis	BARLG069-20	MT372364
Longitarsus foudrasi	db43	Italy, Abruzzo (AQ), Casaline	42.42	13.20	1126m	M. Biondi & P. D'Alessandro	BARLG117-20	MK893904
Longitarsus holsaticus	30D	Italy, Abruzzo (AQ), Lago di Campotosto	42.56	13.34	1315m	E. Berrilli & L. De Vitis	BARLG031-20	MT372365
Longitarsus holsaticus	30C	Italy, Abruzzo (AQ), Lago di Campotosto	42.56	13.34	1315m	E. Berrilli & L. De Vitis	BARLG032-20	MT372367
Longitarsus holsaticus	30B	Italy, Abruzzo (AQ), Lago di Campotosto	42.56	13.34	1315m	E. Berrilli & L. De Vitis	BARLG033-20	MT372366
Longitarsus holsaticus	30A	Italy, Abruzzo (AQ), Lago di Campotosto	42.56	13.34	1315m	E. Berrilli & L. De Vitis	BARLG034-20	MT372368
Longitarsus isoplexidis	12D	Portugal, Madeira Island, Paul da Serra, road to Encumeada	32.74	17.05	1316 m	M. Biondi	BARLG080-20	MT372370
Longitarsus isoplexidis	12B	Portugal, Madeira Island, Paul da Serra, road to Encumeada	32.74	17.05	1316 m	M. Biondi	BARLG081-20	MT372372
Longitarsus isoplexidis	12A	Portugal, Madeira Island, Paul da Serra, road to Encumeada	32.74	17.05	1316 m	M. Biondi	BARLG082-20	MT372369
Longitarsus isoplexidis	db7a	Portugal, Madeira Island, Paul da Serra, road to Encumeada	32.74	17.05	1316 m	M. Biondi	BARLG097-20	MT372371
Longitarsus juncicola	32D	Italy, Calabria (CS), Macchia della Giumenta-San Salvatore	39.38	16.70	1206m	E. Berrilli & L. De Vitis	BARLG023-20	MT372374
Longitarsus juncicola	32C	Italy, Calabria (CS), Macchia della Giumenta-San Salvatore	39.38	16.70	1206m	E. Berrilli & L. De Vitis	BARLG024-20	MT372373
Longitarsus juncicola	32B	Italy, Calabria (CS), Macchia della Giumenta-San Salvatore	39.38	16.70	1206m	E. Berrilli & L. De Vitis	BARLG025-20	MT372375
Longitarsus juncicola	32A	Italy, Calabria (CS), Macchia della Giumenta-San Salvatore	39.38	16.70	1206m	E. Berrilli & L. De Vitis	BARLG026-20	MT372376
Longitarsus lateripunctatus	25C	Italy, Abruzzo (TE), Guazzano	42.73	13.65	563m	M. Biondi & P. D'Alessandro	BARLG049-20	MT372378
Longitarsus lateripunctatus	25B	Italy, Abruzzo (TE), Guazzano	42.73	13.65	563m	M. Biondi & P. D'Alessandro	BARLG050-20	MT372380
Longitarsus lateripunctatus	25A	Italy, Abruzzo (TE), Guazzano	42.73	13.65	563m	M. Biondi & P. D'Alessandro	BARLG051-20	MT372379
Longitarsus lateripunctatus	db16a	Italy, Lazio (RM), Rome	41.88	12.46	50m	M. Biondi	BARLG098-20	MT372377
Longitarsus laureolae	31D	Italy, Sicilia (ME), Bosco di Malabotta	37.97	15.05	1238m	E. Berrilli & L. De Vitis	BARLG027-20	MT372382
Longitarsus laureolae	31C	Italy, Sicilia (ME), Bosco di Malabotta	37.97	15.05	1238m	E. Berrilli & L. De Vitis	BARLG028-20	MT372383
Longitarsus laureolae	31B	Italy, Sicilia (ME), Bosco di Malabotta	37.97	15.05	1238m	E. Berrilli & L. De Vitis	BARLG029-20	MT372381
Longitarsus laureolae	31A	Italy, Sicilia (ME), Bosco di Malabotta	37.97	15.05	1238m	E. Berrilli & L. De Vitis	BARLG030-20	MT372384
Longitarsus ochroleucus lindbergi	24C	Portugal, Madeira Island, Paul da Serra, road to Encumeada	32.74	17.05	1316m	M. Biondi	BARLG052-20	MT372397
Longitarsus ochroleucus lindbergi	24B	Portugal, Madeira Island, Paul da Serra, road to Encumeada	32.74	17.05	1316m	M. Biondi	BARLG053-20	MT372398
Longitarsus ochroleucus lindbergi	24A	Portugal, Madeira Island, Paul da Serra, road to Encumeada	32.74	17.05	1316m	M. Biondi	BARLG054-20	MT372399
Longitarsus ochroleucus lindbergi	db23	Portugal, Madeira Island, Paul da Serra, road to Encumeada	32.74	17.05	1316m	M. Biondi	BARLG100-20	MK893907
Longitarsus luridus	db33	Italy, Abruzzo (AQ), 2.6 km SW of Aprati	42.54	13.45	862m	M. Biondi & P. D'Alessandro	BARLG106-20	MT372385
Longitarsus ordinatus	27C	Italy, Abruzzo (AQ), San Benedetto in Perillis	42.18	13.77	722m	E. Berrilli & L. De Vitis	BARLG042-20	MT372400
Longitarsus lycopi	27B	Italy, Abruzzo (AQ), San Benedetto in Perillis	42.18	13.77	722m	E. Berrilli & L. De Vitis	BARLG043-20	MT372387
Longitarsus lycopi	27A	Italy, Abruzzo (AQ), San Benedetto in Perillis	42.18	13.77	722m	E. Berrilli & L. De Vitis	BARLG044-20	MT372388
Longitarsus lycopi	db50	Italy, Abruzzo (TE), Prato Selva	42.53	13.51	1178m	M. Biondi & P. D'Alessandro	BARLG115-20	MT372386
Longitarsus melanocephalus	23C	Italy, Abruzzo (AQ), 4 km SW of Ortolano	42.50	13.39	1133m	M. Biondi & P. D'Alessandro	BARLG055-20	MT372391
Longitarsus melanocephalus	23B	Italy, Abruzzo (AQ), 4 km SW of Ortolano	42.50	13.39	1133m	M. Biondi & P. D'Alessandro	BARLG056-20	MT372390
Longitarsus melanocephalus	23A	Italy, Abruzzo (AQ), 4 km SW of Ortolano	42.50	13.39	1133m	M. Biondi & P. D'Alessandro	BARLG057-20	MT372389
Longitarsus melanocephalus	db32	Italy, Abruzzo (AQ), 4 km SW of Ortolano	42.50	13.39	1133m	M. Biondi & P. D'Alessandro	BARLG105-20	MT372392
Longitarsus nigrofasciatus	17D	Italy, Abruzzo (AQ), Rovere Sirente	42.17	13.53	1499m	E. Berrilli & G. Simbula	BARLG074-20	MT372393
Longitarsus nigrofasciatus	17C	Italy, Abruzzo (AQ), Rovere Sirente	42.17	13.53	1499m	E. Berrilli & G. Simbula	BARLG075-20	MT372394
Longitarsus nigrofasciatus	17B	Italy, Abruzzo (AQ), Rovere Sirente	42.17	13.53	1499m	E. Berrilli & G. Simbula	BARLG076-20	MT372395
Longitarsus nigrofasciatus	17A	Italy, Abruzzo (AQ), Rovere Sirente	42.17	13.53	1499m	E. Berrilli & G. Simbula	BARLG077-20	MT372396
Longitarsus parvulus	36C	Italy, Abruzzo (PE), Popoli, Contrada S. Callisto	42.18	13.80	431m	E. Berrilli & L. De Vitis	BARLG004-20	MT372401
Longitarsus parvulus	36B	Italy, Abruzzo (PE), Popoli, Contrada S. Callisto	42.18	13.80	431m	E. Berrilli & L. De Vitis	BARLG005-20	MT372404
Longitarsus parvulus	36A	Italy, Abruzzo (PE), Popoli, Contrada S. Callisto	42.18	13.80	431m	E. Berrilli & L. De Vitis	BARLG006-20	MT372403
Longitarsus parvulus	db48	Italy, Abruzzo (PE), Popoli, Contrada S. Callisto	42.18	13.80	431m	E. Berrilli & L. De Vitis	BARLG111-20	MT372402
Longitarsus pellucidus	18D	Italy, Abruzzo (TE), near Guazzano	42.74	13.63	798m	M. Biondi	BARLG070-20	MT372408
Longitarsus pellucidus	18C	Italy, Abruzzo (TE), near Guazzano	42.74	13.63	798m	M. Biondi	BARLG071-20	MT372407
Longitarsus pellucidus	18B	Italy, Abruzzo (TE), near Guazzano	42.74	13.63	798m	M. Biondi	BARLG072-20	MT372406
Longitarsus pellucidus	18A	Italy, Abruzzo (TE), near Guazzano	42.74	13.63	798m	M. Biondi	BARLG073-20	MT372405
Longitarsus pinguis	8B	Italy, Abruzzo (AQ), Campo imperatore	42.44	13.56	2230m	M. Biondi & P. D'Alessandro leg.	BARLG085-20	MT372410
Longitarsus pinguis	8A	Italy, Abruzzo (AQ), Campo imperatore	42.44	13.56	2230m	M. Biondi & P. D'Alessandro leg.	BARLG086-20	MT372409
Longitarsus pinguis	8D	Italy, Abruzzo (AQ), Campo imperatore	42.44	13.56	2230m	M. Biondi & P. D'Alessandro leg.	BARLG094-20	MT372411
Longitarsus pinguis	db21	Italy, Abruzzo (AQ), Campo imperatore	42.44	13.56	1760m	M. Biondi & P. D'Alessandro leg.	BARLG113-20	MK893909
Longitarsus pratensis	6D	Italy, Liguria (IM), Santuario SS. Cosma e Damiano, ,	44.09	7.96	1051m	M. Biondi	BARLG013-20	MT372413
Longitarsus pratensis	6C	Italy, Liguria (IM), Santuario SS. Cosma e Damiano, ,	44.09	7.96	1051m	M. Biondi	BARLG014-20	MT372416
Longitarsus pratensis	6A	Italy, Liguria (IM), Santuario SS. Cosma e Damiano, ,	44.09	7.96	1051m	M. Biondi	BARLG015-20	MT372415
Longitarsus pratensis	db25	Italy, Marche (AP), Colle San Marco	42.66	13.58	858m	F. Cerasoli	BARLG101-20	MT372412
Longitarsus pratensis	db36	Italy, Abruzzo (AQ), Colle Cavallari, near Pizzoli	41.42	13.30	690m	P. D'Alessandro & F. Cerasoli	BARLG108-20	MT372414
Longitarsus rectilineatus	21C	Italy, Lazio (RI), Monti della Duchessa, Val di Fua	42.18	13.32	1300-1500m	M. Biondi	BARLG061-20	MT372417
Longitarsus rectilineatus	21B	Italy, Lazio (RI), Monti della Duchessa, Val di Fua	42.18	13.32	1300-1500m	M. Biondi	BARLG062-20	MT372418
Longitarsus rectilineatus	21A	Italy, Lazio (RI), Monti della Duchessa, Val di Fua	42.18	13.32	1300-1500m	M. Biondi	BARLG063-20	MT372419
Longitarsus rectilineatus	db35	Italy, Lazio (RI), Monti della Duchessa, Val di Fua	42.18	13.32	1300-1500m	M. Biondi	BARLG107-20	MK893910
Longitarsus salviae	37C	Italy, Abruzzo (AQ), Colle Cavallari, near Pizzoli	41.42	13.30	690m	P. D'Alessandro & F. Cerasoli	BARLG001-20	MT372423
Longitarsus salviae	37B	Italy, Abruzzo (AQ), Colle Cavallari, near Pizzoli	41.42	13.30	690m	P. D'Alessandro & F. Cerasoli	BARLG002-20	MT372422
Longitarsus salviae	37A	Italy, Abruzzo (AQ), Colle Cavallari, near Pizzoli	41.42	13.30	690m	P. D'Alessandro & F. Cerasoli	BARLG003-20	MT372421
Longitarsus salviae	db29	Italy, Abruzzo (AQ), Ortolano	42.50	13.39	1133m	M. Biondi	BARLG103-20	MT372420
Longitarsus springeri	20C	Italy, Abruzzo (AQ), Campo Imperatore	42.43	13.61	2140m	M. Biondi	BARLG064-20	MT372425
Longitarsus springeri	20B	Italy, Abruzzo (AQ), Campo Imperatore	42.43	13.61	2140m	M. Biondi	BARLG065-20	MT372424
Longitarsus springeri	20A	Italy, Abruzzo (AQ), Campo Imperatore	42.43	13.61	2140m	M. Biondi	BARLG066-20	MT372426
Longitarsus strigicollis	28C	Italy, Abruzzo (AQ), Area Archeologica Amiternum	42.40	13.31	669m	E. Berrilli, L. De Vitis, M. Biondi	BARLG039-20	MT372427
Longitarsus strigicollis	28B	Italy, Abruzzo (AQ), Area Archeologica Amiternum	42.40	13.31	669m	E. Berrilli, L. De Vitis, M. Biondi	BARLG040-20	MT372428

Longitarsus strigicollis	28A	Italy, Abruzzo (AQ), Area Archeologica Amiternum	42.40	13.31	669m	E. Berrilli, L. De Vitis, M. Biondi	BARLG041-20	MT372429
Longitarsus strigicollis	db31	Italy, Abruzzo (AQ), 4 km SW of Ortolano	42.50	13.39	1133m	M. Biondi & P. D'Alessandro	BARLG104-20	MT372430
Longitarsus succineus	22C	Italy, Liguria (IM), Santuario SS. Cosma e Damiano	44.10	7.98	1060m	M. Biondi	BARLG058-20	MT372434
Longitarsus succineus	22B	Italy, Liguria (IM), Santuario SS. Cosma e Damiano	44.10	7.98	1060m	M. Biondi	BARLG059-20	MT372431
Longitarsus succineus	22A	Italy, Liguria (IM), Santuario SS. Cosma e Damiano	44.10	7.98	1060m	M. Biondi	BARLG060-20	MT372432
Longitarsus succineus	db51	Italy, Abruzzo (AQ), Valico delle Capannelle	42.46	13.35	1312m	M. Biondi & P. D'Alessandro	BARLG109-20	MT372433
Longitarsus tabidus	2	Italiy, Abruzzo (AQ),Meta, Monte Viglio	41.88	13.40	1028m	E. Berrilli & G. Simbula	BARLG016-20	MT372435
Longitarsus tabidus	1B	Italiy, Abruzzo (AQ),Meta, Monte Viglio	41.88	13.40	1028m	E. Berrilli & G. Simbula	BARLG017-20	MT372436
Longitarsus tabidus	1A	Italiy, Abruzzo (AQ),Meta, Monte Viglio	41.88	13.40	1028m	E. Berrilli & G. Simbula	BARLG018-20	MT372437
Longitarsus tabidus	1	Italiy, Abruzzo (AQ),Meta, Monte Viglio	41.88	13.40	1028m	E. Berrilli & G. Simbula	BARLG019-20	MT372438
Longitarsus zangheri	34C	Italy, Abruzzo (TE), Ceppo	42.66	13.46	1412m	E. Berrilli & L. De Vitis	BARLG010-20	MT372439
Longitarsus zangheri	34B	Italy, Abruzzo (TE), Ceppo	42.66	13.46	1412m	E. Berrilli & L. De Vitis	BARLG011-20	MT372440
Longitarsus zangheri	34A	Italy, Abruzzo (TE), Ceppo	42.66	13.46	1412m	E. Berrilli & L. De Vitis	BARLG012-20	MT372441
Longitarsus zangherii	db47	Italy, Abruzzo (TE), Prati di Tivo	42.51	13.55	1344m	M. Biondi & P. D'Alessandro	BARLG116-20	MK893913
