# Supplementary material for: Sharpening the DNA barcoding tool through a posteriori taxonomic validation: The case of Longitarsus flea beetles (Coleoptera: Chrysomelidae)
Source: PLoS One. 2020 May 21;15(5):e0233573. doi: 10.1371/journal.pone.0233573 (PMC7241800; doi:10.1371/journal.pone.0233573)
Supplement: S1 Fig — (PDF) [file pone.0233573.s003.pdf]

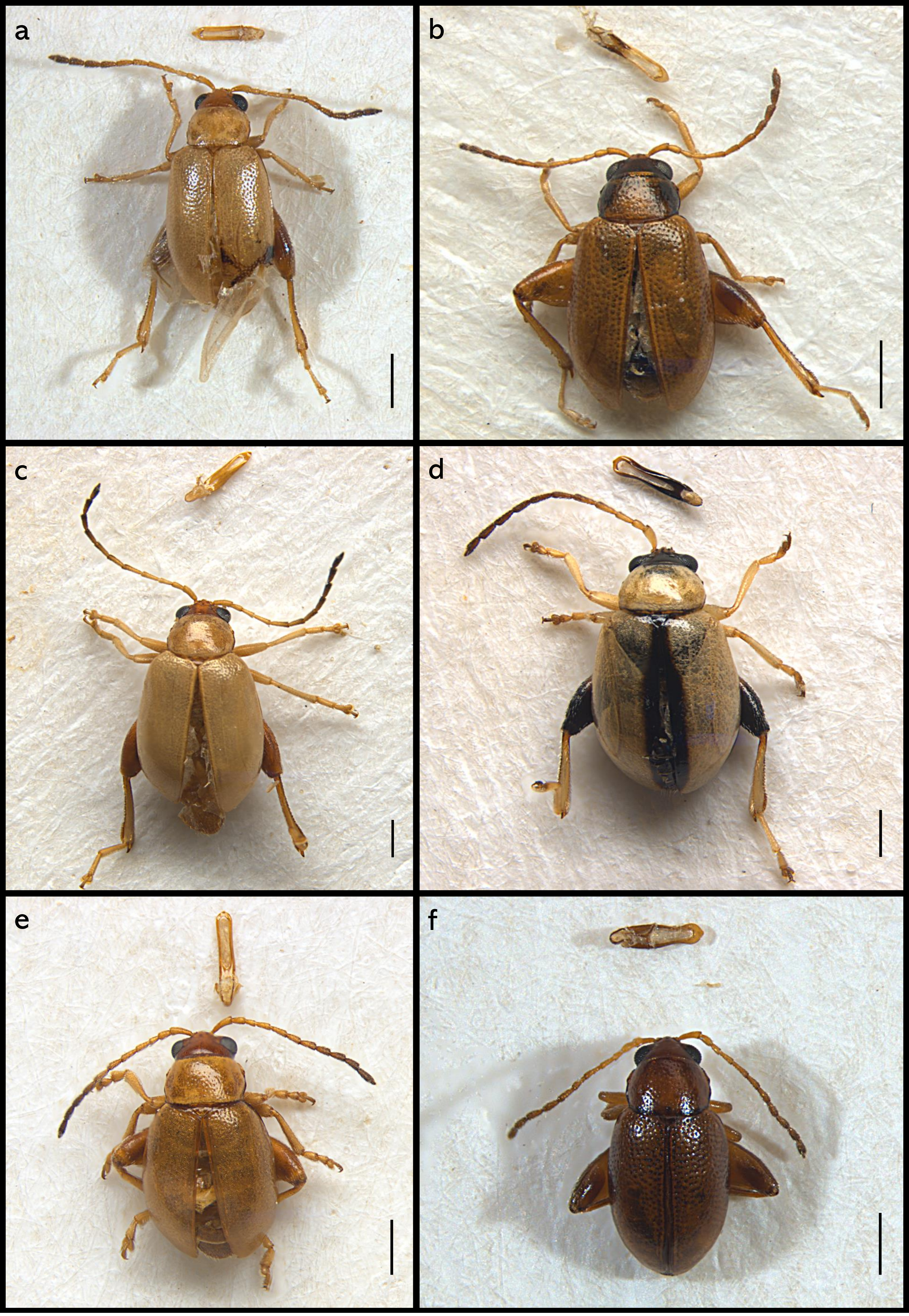

Supplementary Figure S1. Photographs of habitus and aedeagus of (a) *Longitarsus albineus* ♂; (b) *L. exsoletus* ♂; (c) *L. juncicola* ♂; (d) *L. lindbergi* ♂; (e) *L. laureolae* ♂; (f) *L. luridus* ♂. Scale bar 0.5 mm.
