# Supplementary material for: Sharpening the DNA barcoding tool through a posteriori taxonomic validation: The case of Longitarsus flea beetles (Coleoptera: Chrysomelidae)
Source: PLoS One. 2020 May 21;15(5):e0233573. doi: 10.1371/journal.pone.0233573 (PMC7241800; doi:10.1371/journal.pone.0233573)
Supplement: S2 Fig — (PDF) [file pone.0233573.s004.pdf]

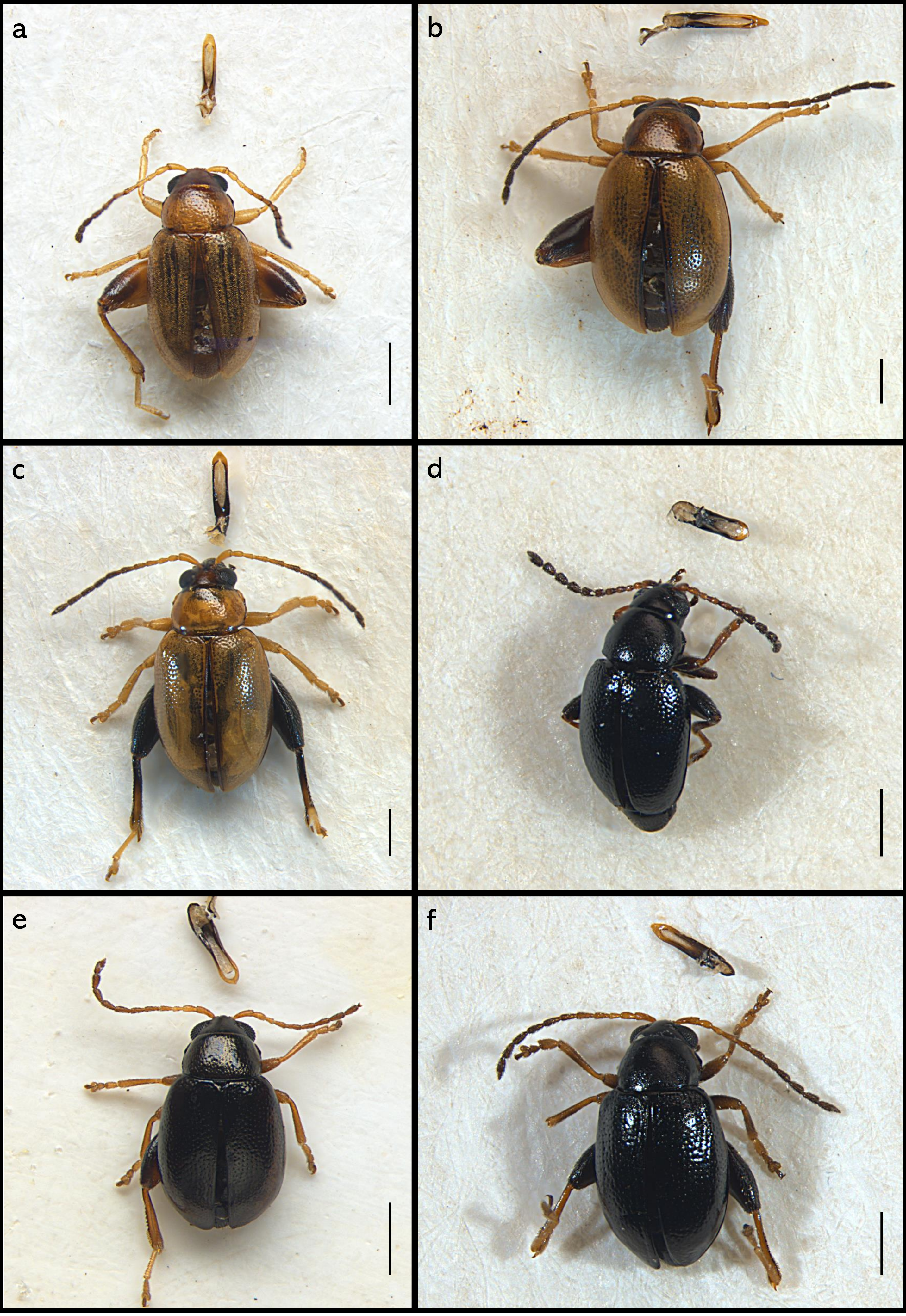

Supplementary Figure S2. Photographs of habitus and aedeagus of (a) *Longitarsus ordinatus* ♂; (b) *L. nigrofasciatus* ♂; (c) *L. melanocephalus* ♂; (d) *L. pinguis* ♂; (e) *L. parvulus* ♂; (f) *L. rectilineatus* ♂. Scale bar 0.5 mm.
