# Supplementary material for: Sharpening the DNA barcoding tool through a posteriori taxonomic validation: The case of Longitarsus flea beetles (Coleoptera: Chrysomelidae)
Source: PLoS One. 2020 May 21;15(5):e0233573. doi: 10.1371/journal.pone.0233573 (PMC7241800; doi:10.1371/journal.pone.0233573)
Supplement: S3 Fig — (PDF) [file pone.0233573.s005.pdf]

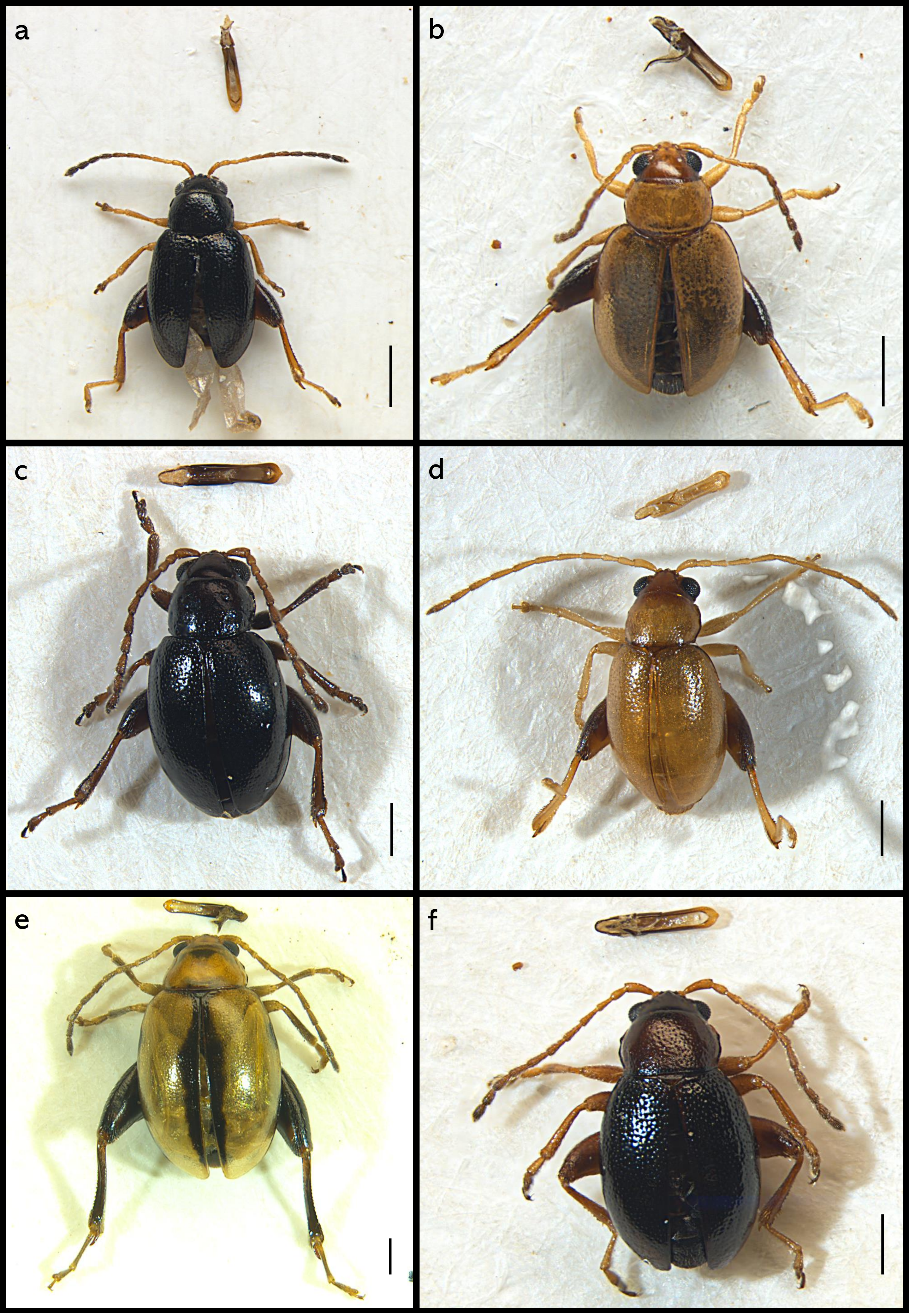

Supplementary Figure S3. Photographs of habitus and aedeagus of (a) *Longitarsus salviae* ♂; (b) *L. strigicollis* ♂; (c) *L. springeri* ♂; (d) *L. succineus* ♂; (e) *L. tabidus* ♂; (f) *L. zangherii* ♂. Scale bar 0.5 mm.
