# Supplementary material for: Sharpening the DNA barcoding tool through a posteriori taxonomic validation: The case of Longitarsus flea beetles (Coleoptera: Chrysomelidae)
Source: PLoS One. 2020 May 21;15(5):e0233573. doi: 10.1371/journal.pone.0233573 (PMC7241800; doi:10.1371/journal.pone.0233573)
Supplement: S4 Fig — For each species two specimens were selected and DNA extracted either with the invasive method, IM, or with the non-invasive method, NIM (bs-IM: R2 = 0.1053, p-value = 0.1407; bs-NIM: R2 = 0.0155, p-value = 0.581; tp-IM: R2 = 0.07261, p-value = 0.2252; tp-NIM: R2 = 0.1191, p-value = 0.1156). (PDF) [file pone.0233573.s006.pdf]

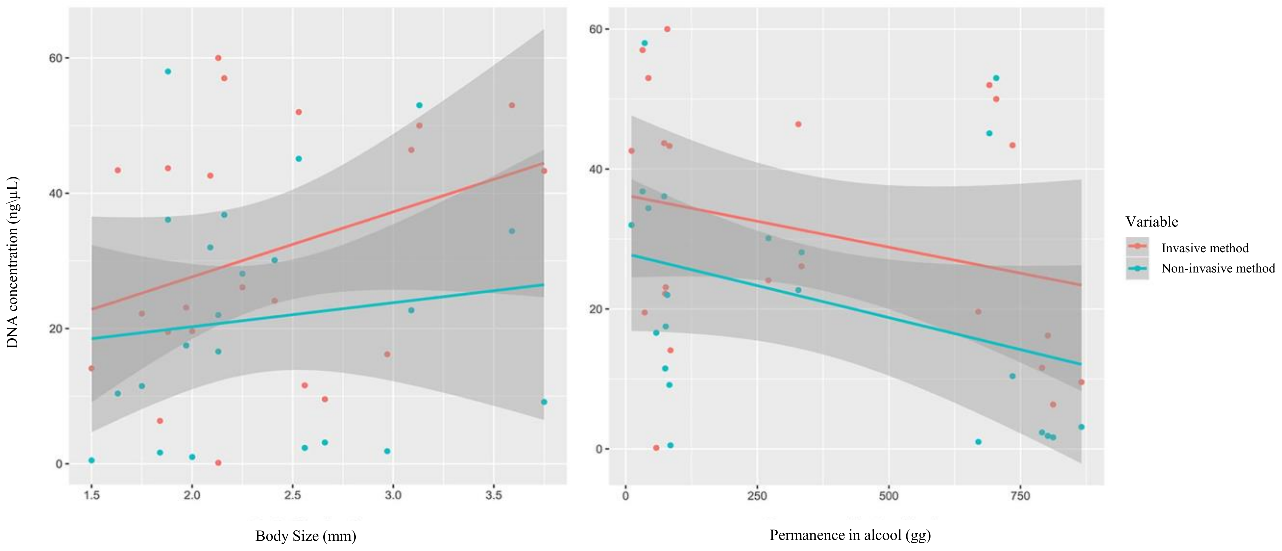

Supplementary Figure S4. Linear regression models showing (a) the relationships between yield of DNA extraction and time of permanence in alcohol of the specimens, *tp*; and (b) the body size of the specimens, *bs*. For each species two specimens were selected and DNA extracted either with the invasive method, IM, or with the non-invasive method, NIM (*bs*-IM: *R2*=0.1053, *p*-value=0.1407; *bs*-NIM: *R2*=0.0155, *p*-value=0.581; *tp*-IM: *R2*=0.07261, *p*-value=0.2252; *tp*-NIM: *R2*=0.1191, *p*-value=0.1156).

.
