# Supplementary material for: Sharpening the DNA barcoding tool through a posteriori taxonomic validation: The case of Longitarsus flea beetles (Coleoptera: Chrysomelidae)
Source: PLoS One. 2020 May 21;15(5):e0233573. doi: 10.1371/journal.pone.0233573 (PMC7241800; doi:10.1371/journal.pone.0233573)
Supplement: S5 Fig — (a) ANOVA results comparing the final amount of the DNA extracted with the two methods. (b) Amplification success and (c) Sanger sequencing success of the cox1 gene fragment using DNA templates obtained with the two DNA extraction methods. Bands obtained with DNA templates extracted with the NIM are marked on the electrophoretic gel. Sequencing success rate for PCR products obtained using DNA templates extracted with the two methods: IM, 90% good, 10% poor and 0% failed; NIM, 91% good, 6% poor and 2% failed. (PDF) [file pone.0233573.s007.pdf]

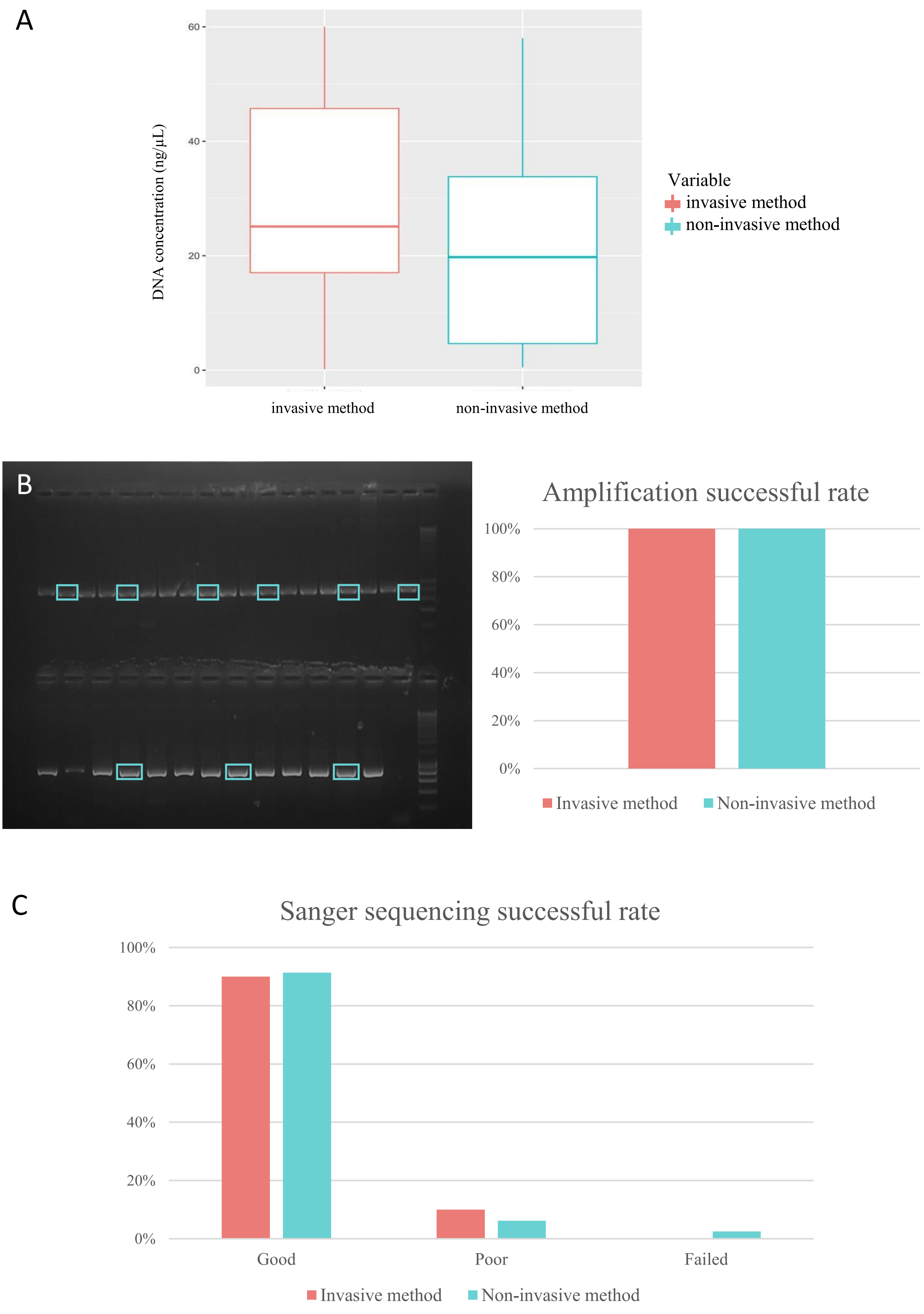

Supplementary Figure S5. Comparison of DNA extraction yield, amplification and sequencing success between the invasive DNA extraction method, IM, and the non-invasive DNA extraction method, NIM. (a) ANOVA results comparing the final amount of the DNA extracted with the two methods. (b) Amplification success and (c) Sanger sequencing success of the cox1 gene fragment using DNA templates obtained with the two DNA extraction methods. Bands obtained with DNA templates extracted with the NIM are marked on the electrophoretic gel. Sequencing success rate for PCR products obtained using DNA templates extracted with the two methods: IM, 90% good, 10% poor and 0% failed; NIM, 91% good, 6% poor and 2% failed.
