# Supplementary material for: Sharpening the DNA barcoding tool through a posteriori taxonomic validation: The case of Longitarsus flea beetles (Coleoptera: Chrysomelidae)
Source: PLoS One. 2020 May 21;15(5):e0233573. doi: 10.1371/journal.pone.0233573 (PMC7241800; doi:10.1371/journal.pone.0233573)
Supplement: S8 Fig — (PDF) [file pone.0233573.s010.pdf]

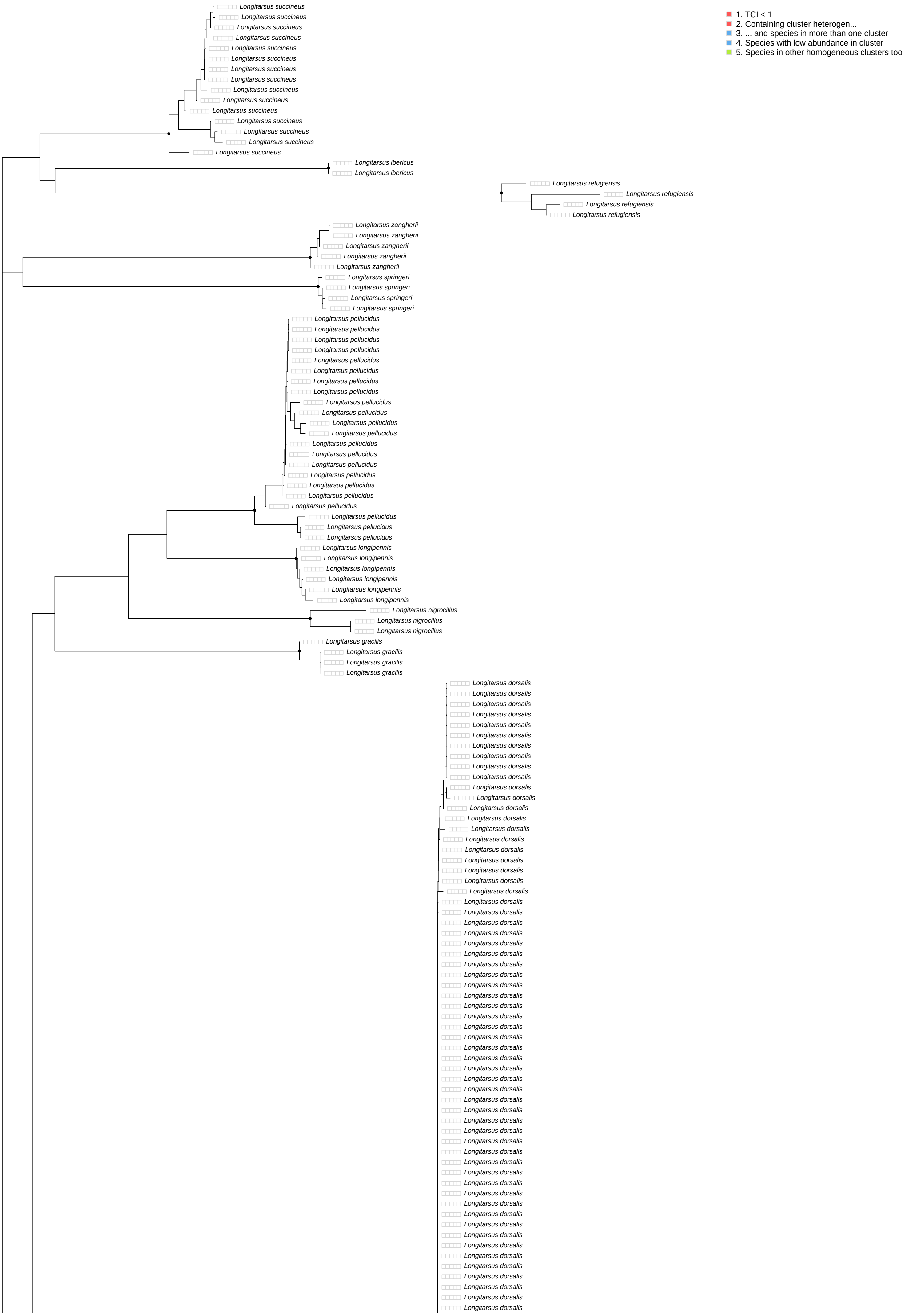
1. TCI < 1
2. Containing cluster heterogen...
3. ... and species in more than one cluster
4. Species with low abundance in cluster
5. Species in other homogeneous clusters too
Longitarsus succineus
Longitarsus succineus
Longitarsus succineus
Longitarsus succineus
Longitarsus succineus
Longitarsus succineus
Longitarsus succineus
Longitarsus succineus
Longitarsus succineus
Longitarsus succineus
Longitarsus succineus
Longitarsus succineus
Longitarsus succineus
Longitarsus succineus
Longitarsus succineus
Longitarsus ibericus
Longitarsus ibericus
Longitarsus refugiensis
Longitarsus refugiensis
Longitarsus refugiensis
Longitarsus refugiensis
Longitarsus zangherii
Longitarsus zangherii
Longitarsus zangherii
Longitarsus zangherii
Longitarsus zangherii
Longitarsus springeri
Longitarsus springeri
Longitarsus springeri
Longitarsus springeri
Longitarsus pellucidus
Longitarsus pellucidus
Longitarsus pellucidus
Longitarsus pellucidus
Longitarsus pellucidus
Longitarsus pellucidus
Longitarsus pellucidus
Longitarsus pellucidus
Longitarsus pellucidus
Longitarsus pellucidus
Longitarsus pellucidus
Longitarsus pellucidus
Longitarsus pellucidus
Longitarsus pellucidus
Longitarsus pellucidus
Longitarsus pellucidus
Longitarsus pellucidus
Longitarsus pellucidus
Longitarsus pellucidus
Longitarsus pellucidus
Longitarsus pellucidus
Longitarsus pellucidus
Longitarsus longipennis
Longitarsus longipennis
Longitarsus longipennis
Longitarsus longipennis
Longitarsus longipennis
Longitarsus longipennis
Longitarsus nigrocillus
Longitarsus nigrocillus
Longitarsus nigrocillus
Longitarsus gracilis
Longitarsus gracilis
Longitarsus gracilis
Longitarsus gracilis
Longitarsus dorsalis
Longitarsus dorsalis
Longitarsus dorsalis
Longitarsus dorsalis
Longitarsus dorsalis
Longitarsus dorsalis
Longitarsus dorsalis
Longitarsus dorsalis
Longitarsus dorsalis
Longitarsus dorsalis
Longitarsus dorsalis
Longitarsus dorsalis
Longitarsus dorsalis
Longitarsus dorsalis
Longitarsus dorsalis
Longitarsus dorsalis
Longitarsus dorsalis
Longitarsus dorsalis
Longitarsus dorsalis
Longitarsus dorsalis
Longitarsus dorsalis
Longitarsus dorsalis
Longitarsus dorsalis
Longitarsus dorsalis
Longitarsus dorsalis
Longitarsus dorsalis
Longitarsus dorsalis
Longitarsus dorsalis
Longitarsus dorsalis
Longitarsus dorsalis
Longitarsus dorsalis
Longitarsus dorsalis
Longitarsus dorsalis
Longitarsus dorsalis
Longitarsus dorsalis
Longitarsus dorsalis
Longitarsus dorsalis
Longitarsus dorsalis
Longitarsus dorsalis
Longitarsus dorsalis
Longitarsus dorsalis
Longitarsus dorsalis
Longitarsus dorsalis
Longitarsus dorsalis
Longitarsus dorsalis
Longitarsus dorsalis
Longitarsus dorsalis
Longitarsus dorsalis
Longitarsus dorsalis
Longitarsus dorsalis
Longitarsus dorsalis
Longitarsus dorsalis
Longitarsus dorsalis
Longitarsus dorsalis
Longitarsus dorsalis
Longitarsus dorsalis
Longitarsus dorsalis
Longitarsus dorsalis
Longitarsus dorsalis
Longitarsus dorsalis
Longitarsus dorsalis
Longitarsus dorsalis
Longitarsus dorsalis
Longitarsus dorsalis
Longitarsus dorsalis

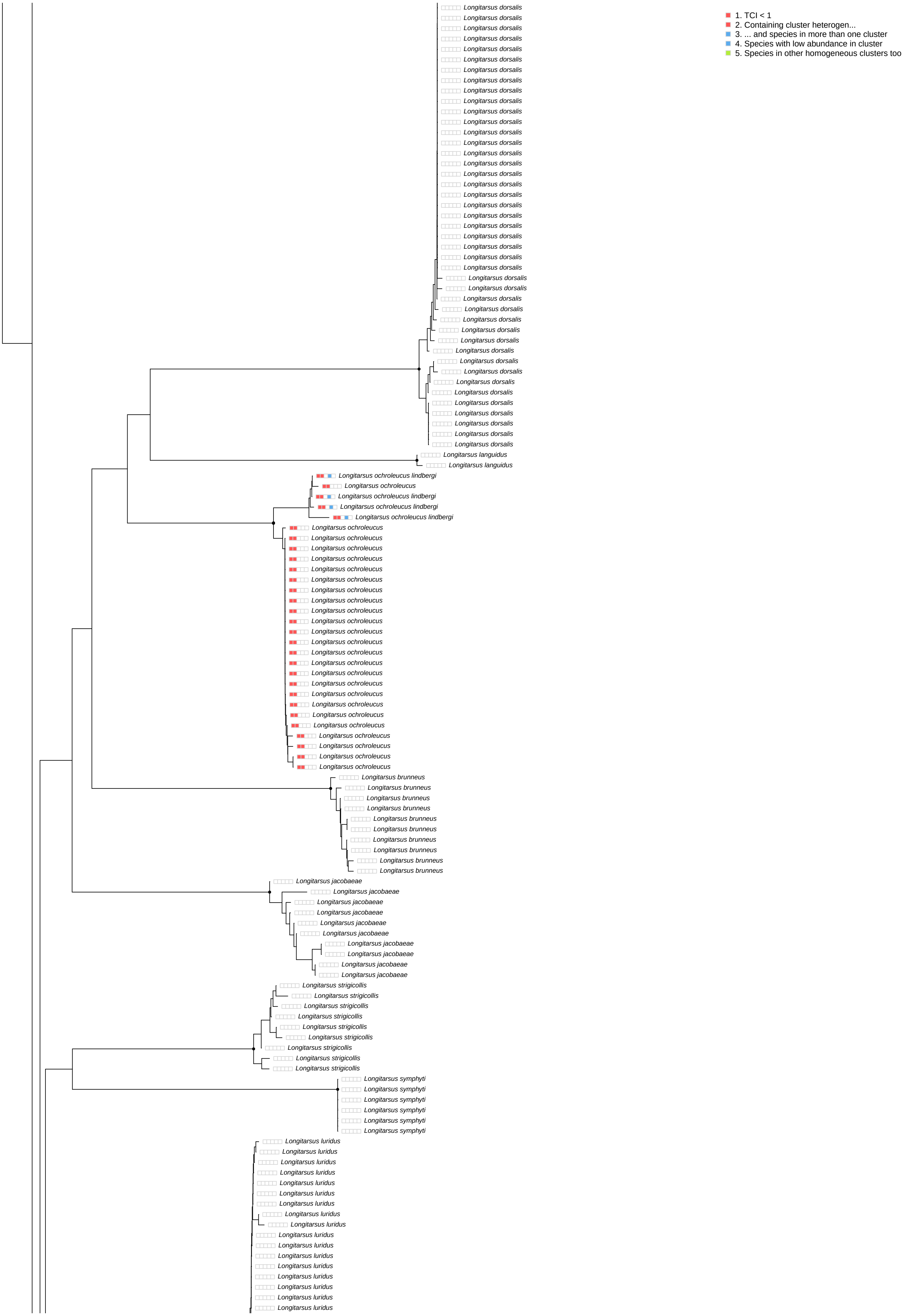
1. TCI < 1
2. Containing cluster heterogen...
3. ... and species in more than one cluster
4. Species with low abundance in cluster
5. Species in other homogeneous clusters too
Longitarsus dorsalis
Longitarsus dorsalis
Longitarsus dorsalis
Longitarsus dorsalis
Longitarsus dorsalis
Longitarsus dorsalis
Longitarsus dorsalis
Longitarsus dorsalis
Longitarsus dorsalis
Longitarsus dorsalis
Longitarsus dorsalis
Longitarsus dorsalis
Longitarsus dorsalis
Longitarsus dorsalis
Longitarsus dorsalis
Longitarsus dorsalis
Longitarsus dorsalis
Longitarsus dorsalis
Longitarsus dorsalis
Longitarsus dorsalis
Longitarsus dorsalis
Longitarsus dorsalis
Longitarsus dorsalis
Longitarsus dorsalis
Longitarsus dorsalis
Longitarsus dorsalis
Longitarsus dorsalis
Longitarsus dorsalis
Longitarsus dorsalis
Longitarsus dorsalis
Longitarsus dorsalis
Longitarsus dorsalis
Longitarsus dorsalis
Longitarsus dorsalis
Longitarsus dorsalis
Longitarsus dorsalis
Longitarsus dorsalis
Longitarsus dorsalis
Longitarsus dorsalis
Longitarsus dorsalis
Longitarsus dorsalis
Longitarsus dorsalis
Longitarsus dorsalis
Longitarsus languidus
Longitarsus languidus
Longitarsus ochroleucus lindbergi
Longitarsus ochroleucus
Longitarsus ochroleucus lindbergi
Longitarsus ochroleucus lindbergi
Longitarsus ochroleucus lindbergi
Longitarsus ochroleucus
Longitarsus ochroleucus
Longitarsus ochroleucus
Longitarsus ochroleucus
Longitarsus ochroleucus
Longitarsus ochroleucus
Longitarsus ochroleucus
Longitarsus ochroleucus
Longitarsus ochroleucus
Longitarsus ochroleucus
Longitarsus ochroleucus
Longitarsus ochroleucus
Longitarsus ochroleucus
Longitarsus ochroleucus
Longitarsus ochroleucus
Longitarsus ochroleucus
Longitarsus ochroleucus
Longitarsus ochroleucus
Longitarsus ochroleucus
Longitarsus ochroleucus
Longitarsus ochroleucus
Longitarsus ochroleucus
Longitarsus ochroleucus
Longitarsus ochroleucus
Longitarsus brunneus
Longitarsus brunneus
Longitarsus brunneus
Longitarsus brunneus
Longitarsus brunneus
Longitarsus brunneus
Longitarsus brunneus
Longitarsus brunneus
Longitarsus brunneus
Longitarsus brunneus
Longitarsus jacobaeae
Longitarsus jacobaeae
Longitarsus jacobaeae
Longitarsus jacobaeae
Longitarsus jacobaeae
Longitarsus jacobaeae
Longitarsus jacobaeae
Longitarsus jacobaeae
Longitarsus jacobaeae
Longitarsus jacobaeae
Longitarsus strigicollis
Longitarsus strigicollis
Longitarsus strigicollis
Longitarsus strigicollis
Longitarsus strigicollis
Longitarsus strigicollis
Longitarsus strigicollis
Longitarsus strigicollis
Longitarsus strigicollis
Longitarsus symphyti
Longitarsus symphyti
Longitarsus symphyti
Longitarsus symphyti
Longitarsus symphyti
Longitarsus symphyti
Longitarsus luridus
Longitarsus luridus
Longitarsus luridus
Longitarsus luridus
Longitarsus luridus
Longitarsus luridus
Longitarsus luridus
Longitarsus luridus
Longitarsus luridus
Longitarsus luridus
Longitarsus luridus
Longitarsus luridus
Longitarsus luridus
Longitarsus luridus
Longitarsus luridus
Longitarsus luridus
Longitarsus luridus

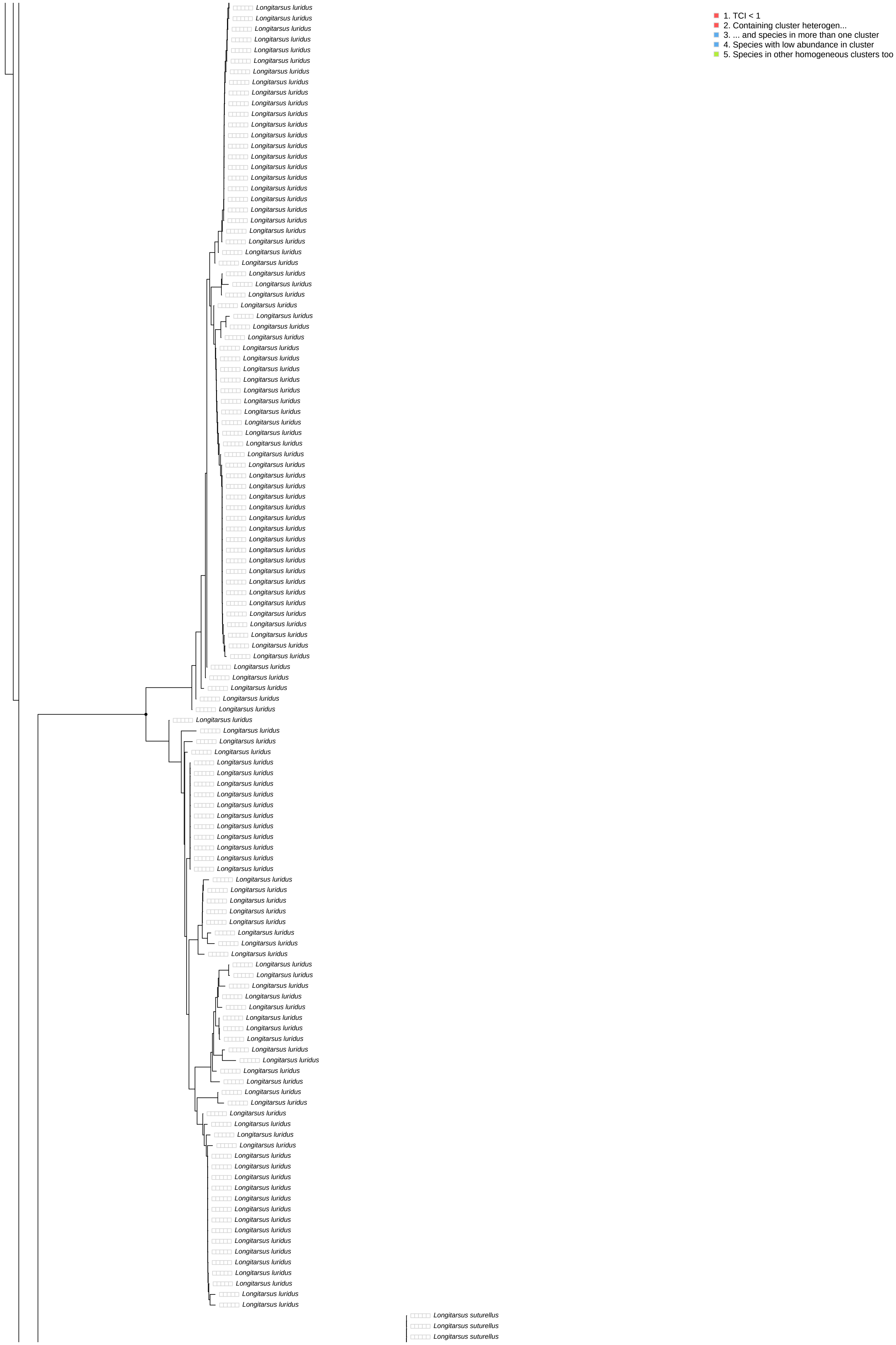
1. TCI < 1
2. Containing cluster heterogen...
3. ... and species in more than one cluster
4. Species with low abundance in cluster
5. Species in other homogeneous clusters too
Longitarsus luridus
Longitarsus luridus
Longitarsus luridus
Longitarsus luridus
Longitarsus luridus
Longitarsus luridus
Longitarsus luridus
Longitarsus luridus
Longitarsus luridus
Longitarsus luridus
Longitarsus luridus
Longitarsus luridus
Longitarsus luridus
Longitarsus luridus
Longitarsus luridus
Longitarsus luridus
Longitarsus luridus
Longitarsus luridus
Longitarsus luridus
Longitarsus luridus
Longitarsus luridus
Longitarsus luridus
Longitarsus luridus
Longitarsus luridus
Longitarsus luridus
Longitarsus luridus
Longitarsus luridus
Longitarsus luridus
Longitarsus luridus
Longitarsus luridus
Longitarsus luridus
Longitarsus luridus
Longitarsus luridus
Longitarsus luridus
Longitarsus luridus
Longitarsus luridus
Longitarsus luridus
Longitarsus luridus
Longitarsus luridus
Longitarsus luridus
Longitarsus luridus
Longitarsus luridus
Longitarsus luridus
Longitarsus luridus
Longitarsus luridus
Longitarsus luridus
Longitarsus luridus
Longitarsus luridus
Longitarsus luridus
Longitarsus luridus
Longitarsus luridus
Longitarsus luridus
Longitarsus luridus
Longitarsus luridus
Longitarsus luridus
Longitarsus luridus
Longitarsus luridus
Longitarsus luridus
Longitarsus luridus
Longitarsus luridus
Longitarsus luridus
Longitarsus luridus
Longitarsus luridus
Longitarsus luridus
Longitarsus luridus
Longitarsus luridus
Longitarsus luridus
Longitarsus luridus
Longitarsus luridus
Longitarsus luridus
Longitarsus luridus
Longitarsus luridus
Longitarsus luridus
Longitarsus luridus
Longitarsus luridus
Longitarsus luridus
Longitarsus luridus
Longitarsus luridus
Longitarsus luridus
Longitarsus luridus
Longitarsus luridus
Longitarsus luridus
Longitarsus luridus
Longitarsus luridus
Longitarsus luridus
Longitarsus luridus
Longitarsus luridus
Longitarsus luridus
Longitarsus luridus
Longitarsus luridus
Longitarsus luridus
Longitarsus luridus
Longitarsus luridus
Longitarsus luridus
Longitarsus luridus
Longitarsus luridus
Longitarsus luridus
Longitarsus luridus
Longitarsus luridus
Longitarsus luridus
Longitarsus luridus
Longitarsus luridus
Longitarsus luridus
Longitarsus luridus
Longitarsus luridus
Longitarsus luridus
Longitarsus luridus
Longitarsus luridus
Longitarsus luridus
Longitarsus luridus
Longitarsus luridus
Longitarsus luridus
Longitarsus luridus
Longitarsus luridus
Longitarsus luridus
Longitarsus luridus
Longitarsus luridus
Longitarsus luridus
Longitarsus luridus
Longitarsus luridus
Longitarsus luridus
Longitarsus luridus
Longitarsus luridus
Longitarsus suturellus
Longitarsus suturellus
Longitarsus suturellus

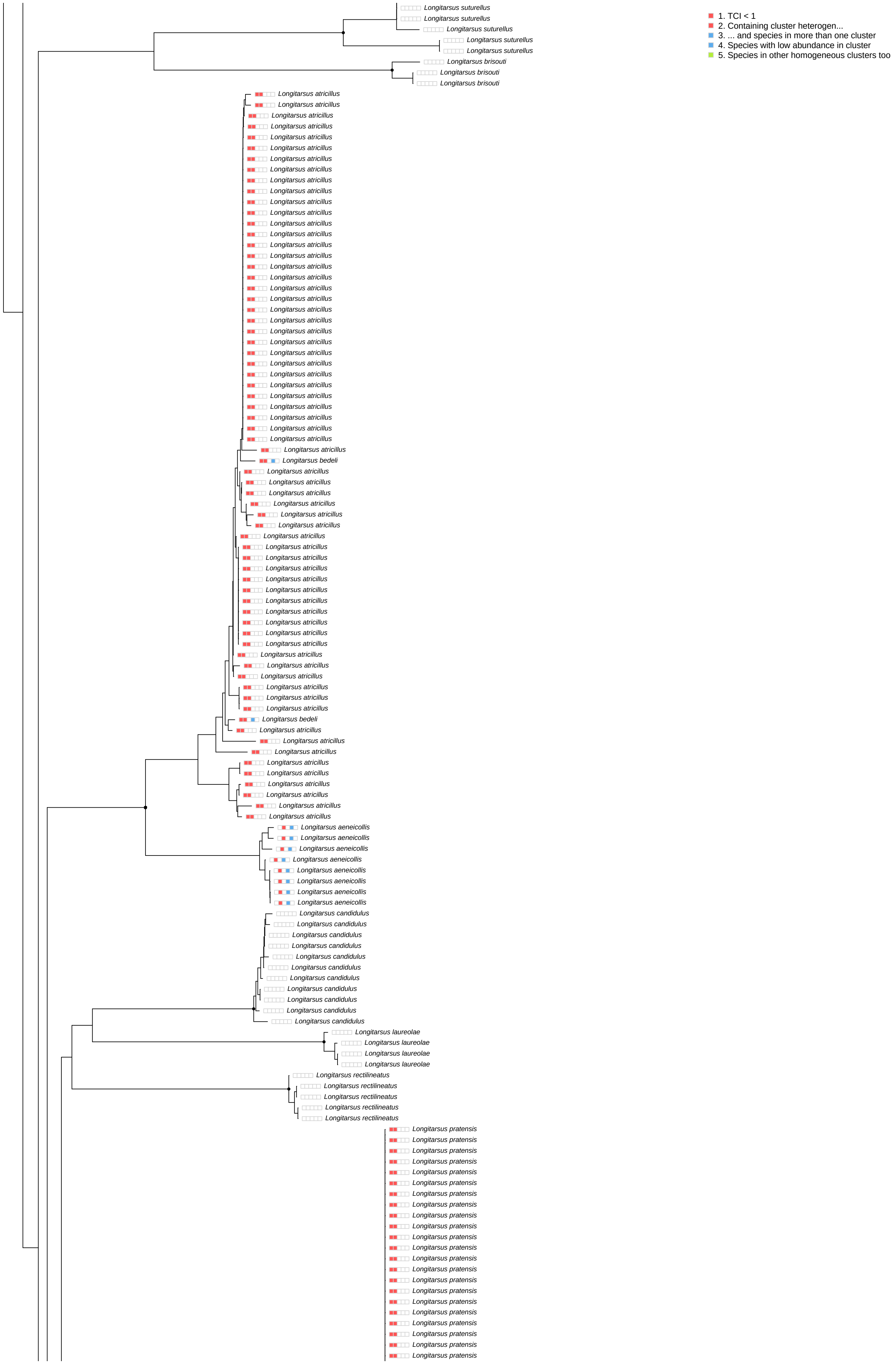
1. TCI < 1
2. Containing cluster heterogen...
3. ... and species in more than one cluster
4. Species with low abundance in cluster
5. Species in other homogeneous clusters too
Longitarsus suturellus
Longitarsus suturellus
Longitarsus suturellus
Longitarsus suturellus
Longitarsus suturellus
Longitarsus brisouti
Longitarsus brisouti
Longitarsus brisouti
Longitarsus atricillus
Longitarsus atricillus
Longitarsus atricillus
Longitarsus atricillus
Longitarsus atricillus
Longitarsus atricillus
Longitarsus atricillus
Longitarsus atricillus
Longitarsus atricillus
Longitarsus atricillus
Longitarsus atricillus
Longitarsus atricillus
Longitarsus atricillus
Longitarsus atricillus
Longitarsus atricillus
Longitarsus atricillus
Longitarsus atricillus
Longitarsus atricillus
Longitarsus atricillus
Longitarsus atricillus
Longitarsus atricillus
Longitarsus atricillus
Longitarsus atricillus
Longitarsus atricillus
Longitarsus atricillus
Longitarsus atricillus
Longitarsus atricillus
Longitarsus atricillus
Longitarsus atricillus
Longitarsus atricillus
Longitarsus atricillus
Longitarsus atricillus
Longitarsus atricillus
Longitarsus atricillus
Longitarsus bedeli
Longitarsus atricillus
Longitarsus atricillus
Longitarsus atricillus
Longitarsus atricillus
Longitarsus atricillus
Longitarsus atricillus
Longitarsus atricillus
Longitarsus atricillus
Longitarsus atricillus
Longitarsus atricillus
Longitarsus atricillus
Longitarsus atricillus
Longitarsus atricillus
Longitarsus atricillus
Longitarsus atricillus
Longitarsus atricillus
Longitarsus atricillus
Longitarsus atricillus
Longitarsus atricillus
Longitarsus atricillus
Longitarsus atricillus
Longitarsus atricillus
Longitarsus atricillus
Longitarsus bedeli
Longitarsus atricillus
Longitarsus atricillus
Longitarsus atricillus
Longitarsus atricillus
Longitarsus atricillus
Longitarsus atricillus
Longitarsus atricillus
Longitarsus atricillus
Longitarsus atricillus
Longitarsus aeneicollis
Longitarsus aeneicollis
Longitarsus aeneicollis
Longitarsus aeneicollis
Longitarsus aeneicollis
Longitarsus aeneicollis
Longitarsus aeneicollis
Longitarsus aeneicollis
Longitarsus candidulus
Longitarsus candidulus
Longitarsus candidulus
Longitarsus candidulus
Longitarsus candidulus
Longitarsus candidulus
Longitarsus candidulus
Longitarsus candidulus
Longitarsus candidulus
Longitarsus candidulus
Longitarsus candidulus
Longitarsus laureolae
Longitarsus laureolae
Longitarsus laureolae
Longitarsus laureolae
Longitarsus rectilineatus
Longitarsus rectilineatus
Longitarsus rectilineatus
Longitarsus rectilineatus
Longitarsus rectilineatus
Longitarsus pratensis
Longitarsus pratensis
Longitarsus pratensis
Longitarsus pratensis
Longitarsus pratensis
Longitarsus pratensis
Longitarsus pratensis
Longitarsus pratensis
Longitarsus pratensis
Longitarsus pratensis
Longitarsus pratensis
Longitarsus pratensis
Longitarsus pratensis
Longitarsus pratensis
Longitarsus pratensis
Longitarsus pratensis
Longitarsus pratensis
Longitarsus pratensis
Longitarsus pratensis
Longitarsus pratensis
Longitarsus pratensis
Longitarsus pratensis

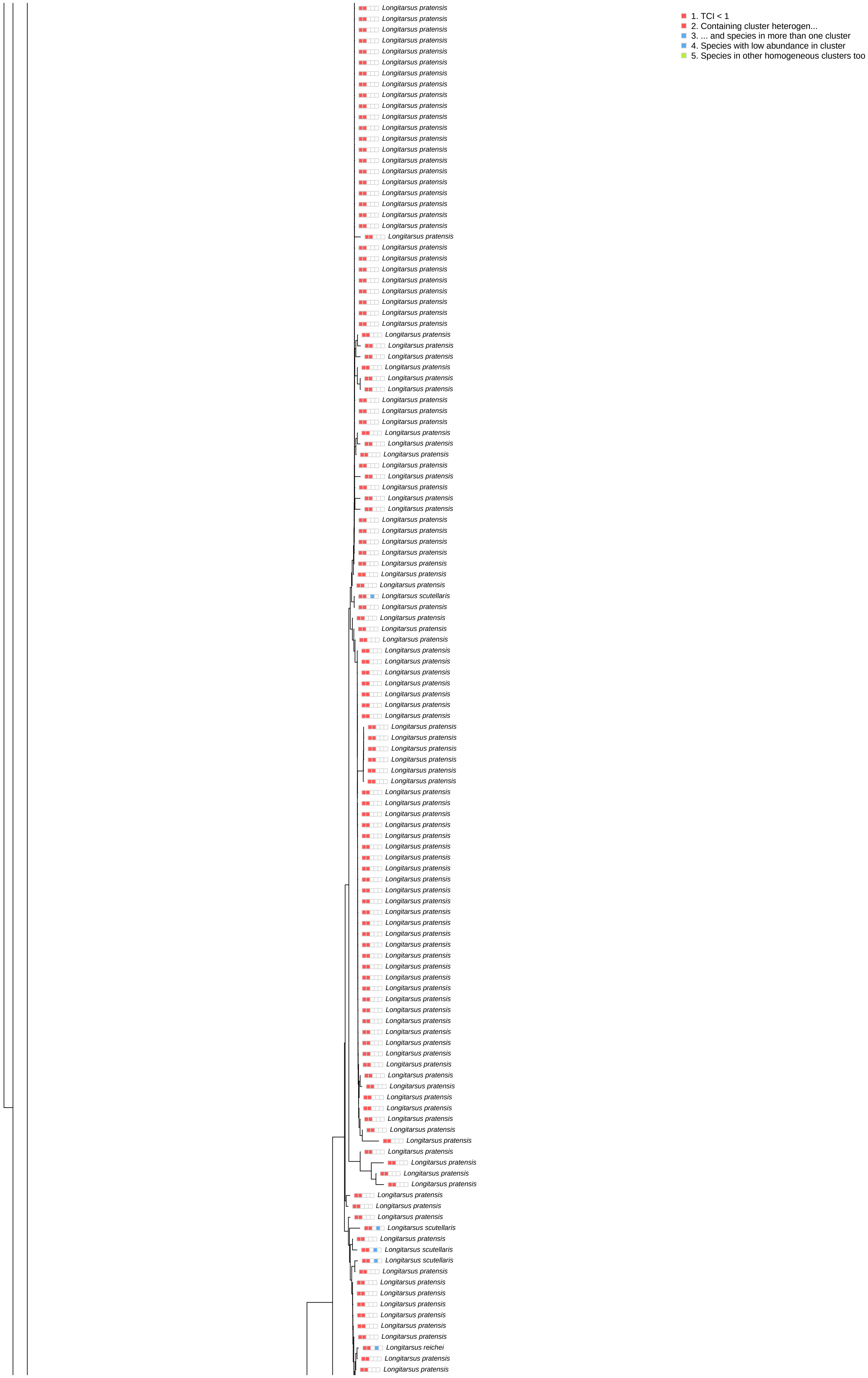

1. TCI < 1
2. Containing cluster heterogen...
3. ... and species in more than one cluster
4. Species with low abundance in cluster
5. Species in other homogeneous clusters too
Longitarsus pratensis
Longitarsus pratensis
Longitarsus pratensis
Longitarsus pratensis
Longitarsus pratensis
Longitarsus pratensis
Longitarsus pratensis
Longitarsus pratensis
Longitarsus pratensis
Longitarsus pratensis
Longitarsus pratensis
Longitarsus pratensis
Longitarsus pratensis
Longitarsus pratensis
Longitarsus pratensis
Longitarsus pratensis
Longitarsus pratensis
Longitarsus pratensis
Longitarsus pratensis
Longitarsus pratensis
Longitarsus pratensis
Longitarsus pratensis
Longitarsus pratensis
Longitarsus pratensis
Longitarsus pratensis
Longitarsus pratensis
Longitarsus pratensis
Longitarsus pratensis
Longitarsus pratensis
Longitarsus pratensis
Longitarsus pratensis
Longitarsus pratensis
Longitarsus pratensis
Longitarsus pratensis
Longitarsus pratensis
Longitarsus pratensis
Longitarsus pratensis
Longitarsus pratensis
Longitarsus pratensis
Longitarsus pratensis
Longitarsus pratensis
Longitarsus pratensis
Longitarsus pratensis
Longitarsus pratensis
Longitarsus pratensis
Longitarsus pratensis
Longitarsus pratensis
Longitarsus pratensis
Longitarsus pratensis
Longitarsus pratensis
Longitarsus pratensis
Longitarsus pratensis
Longitarsus pratensis
Longitarsus pratensis
Longitarsus scutellaris
Longitarsus pratensis
Longitarsus pratensis
Longitarsus pratensis
Longitarsus pratensis
Longitarsus pratensis
Longitarsus pratensis
Longitarsus pratensis
Longitarsus pratensis
Longitarsus pratensis
Longitarsus pratensis
Longitarsus pratensis
Longitarsus pratensis
Longitarsus pratensis
Longitarsus pratensis
Longitarsus pratensis
Longitarsus pratensis
Longitarsus pratensis
Longitarsus pratensis
Longitarsus pratensis
Longitarsus pratensis
Longitarsus pratensis
Longitarsus pratensis
Longitarsus pratensis
Longitarsus pratensis
Longitarsus pratensis
Longitarsus pratensis
Longitarsus pratensis
Longitarsus pratensis
Longitarsus pratensis
Longitarsus pratensis
Longitarsus pratensis
Longitarsus pratensis
Longitarsus pratensis
Longitarsus pratensis
Longitarsus pratensis
Longitarsus pratensis
Longitarsus pratensis
Longitarsus pratensis
Longitarsus pratensis
Longitarsus pratensis
Longitarsus pratensis
Longitarsus pratensis
Longitarsus pratensis
Longitarsus pratensis
Longitarsus pratensis
Longitarsus pratensis
Longitarsus pratensis
Longitarsus pratensis
Longitarsus pratensis
Longitarsus pratensis
Longitarsus pratensis
Longitarsus pratensis
Longitarsus pratensis
Longitarsus pratensis
Longitarsus pratensis
Longitarsus pratensis
Longitarsus pratensis
Longitarsus scutellaris
Longitarsus pratensis
Longitarsus scutellaris
Longitarsus scutellaris
Longitarsus pratensis
Longitarsus pratensis
Longitarsus pratensis
Longitarsus pratensis
Longitarsus pratensis
Longitarsus pratensis
Longitarsus pratensis
Longitarsus reichei
Longitarsus pratensis
Longitarsus pratensis

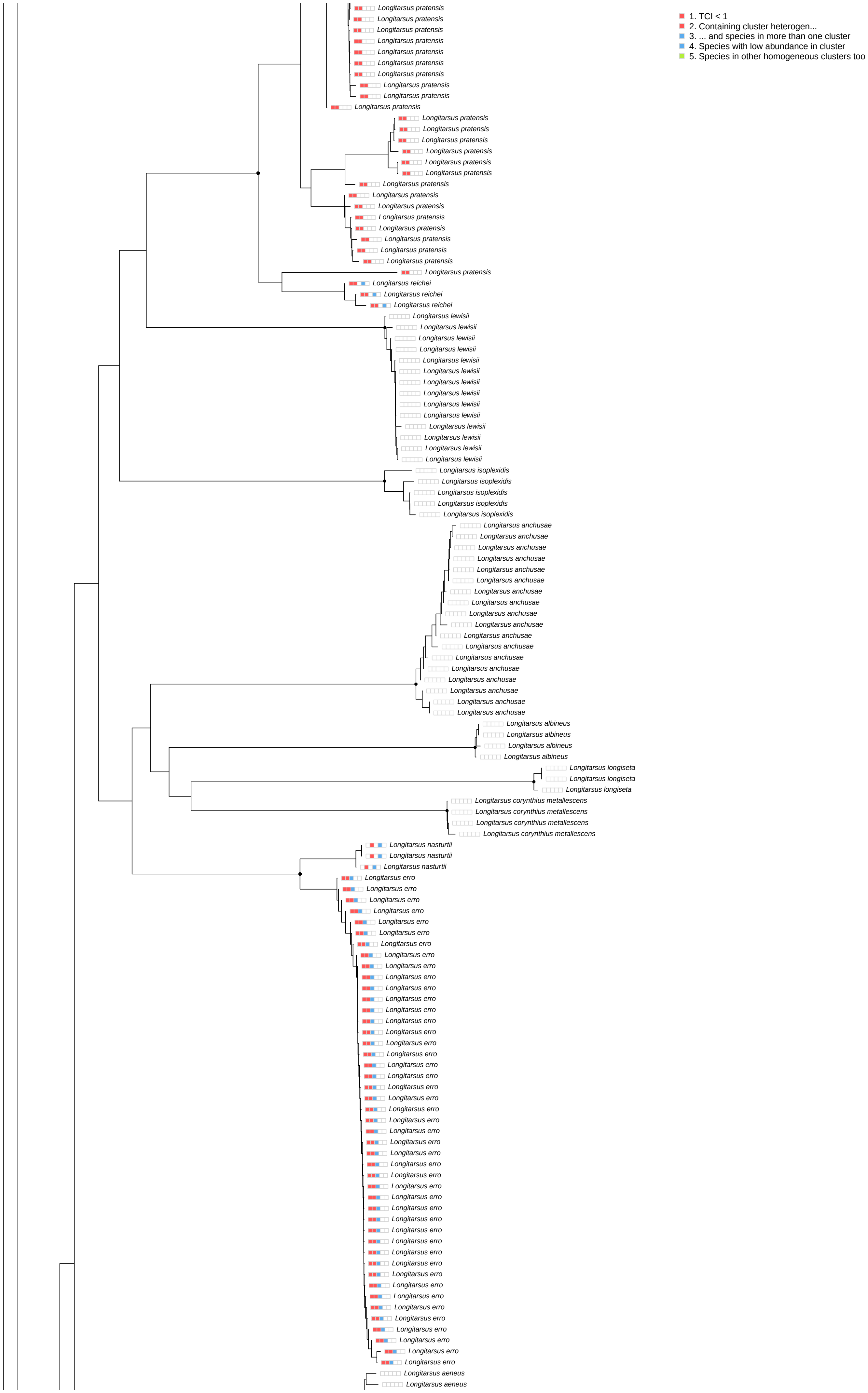
1. TCI < 1
2. Containing cluster heterogen...
3. ... and species in more than one cluster
4. Species with low abundance in cluster
5. Species in other homogeneous clusters too
Longitarsus pratensis
Longitarsus pratensis
Longitarsus pratensis
Longitarsus pratensis
Longitarsus pratensis
Longitarsus pratensis
Longitarsus pratensis
Longitarsus pratensis
Longitarsus pratensis
Longitarsus pratensis
Longitarsus pratensis
Longitarsus pratensis
Longitarsus pratensis
Longitarsus pratensis
Longitarsus pratensis
Longitarsus pratensis
Longitarsus pratensis
Longitarsus pratensis
Longitarsus pratensis
Longitarsus pratensis
Longitarsus pratensis
Longitarsus pratensis
Longitarsus pratensis
Longitarsus pratensis
Longitarsus pratensis
Longitarsus reichei
Longitarsus reichei
Longitarsus reichei
Longitarsus lewisii
Longitarsus lewisii
Longitarsus lewisii
Longitarsus lewisii
Longitarsus lewisii
Longitarsus lewisii
Longitarsus lewisii
Longitarsus lewisii
Longitarsus lewisii
Longitarsus lewisii
Longitarsus lewisii
Longitarsus lewisii
Longitarsus lewisii
Longitarsus lewisii
Longitarsus isoplexidis
Longitarsus isoplexidis
Longitarsus isoplexidis
Longitarsus isoplexidis
Longitarsus isoplexidis
Longitarsus anchusae
Longitarsus anchusae
Longitarsus anchusae
Longitarsus anchusae
Longitarsus anchusae
Longitarsus anchusae
Longitarsus anchusae
Longitarsus anchusae
Longitarsus anchusae
Longitarsus anchusae
Longitarsus anchusae
Longitarsus anchusae
Longitarsus anchusae
Longitarsus anchusae
Longitarsus anchusae
Longitarsus anchusae
Longitarsus anchusae
Longitarsus anchusae
Longitarsus albineus
Longitarsus albineus
Longitarsus albineus
Longitarsus albineus
Longitarsus longiseta
Longitarsus longiseta
Longitarsus longiseta
Longitarsus corynthius metallescens
Longitarsus corynthius metallescens
Longitarsus corynthius metallescens
Longitarsus corynthius metallescens
Longitarsus nasturtii
Longitarsus nasturtii
Longitarsus nasturtii
Longitarsus erro
Longitarsus erro
Longitarsus erro
Longitarsus erro
Longitarsus erro
Longitarsus erro
Longitarsus erro
Longitarsus erro
Longitarsus erro
Longitarsus erro
Longitarsus erro
Longitarsus erro
Longitarsus erro
Longitarsus erro
Longitarsus erro
Longitarsus erro
Longitarsus erro
Longitarsus erro
Longitarsus erro
Longitarsus erro
Longitarsus erro
Longitarsus erro
Longitarsus erro
Longitarsus erro
Longitarsus erro
Longitarsus erro
Longitarsus erro
Longitarsus erro
Longitarsus erro
Longitarsus erro
Longitarsus erro
Longitarsus erro
Longitarsus erro
Longitarsus erro
Longitarsus erro
Longitarsus erro
Longitarsus erro
Longitarsus erro
Longitarsus erro
Longitarsus erro
Longitarsus erro
Longitarsus erro
Longitarsus erro
Longitarsus erro
Longitarsus erro
Longitarsus aeneus
Longitarsus aeneus

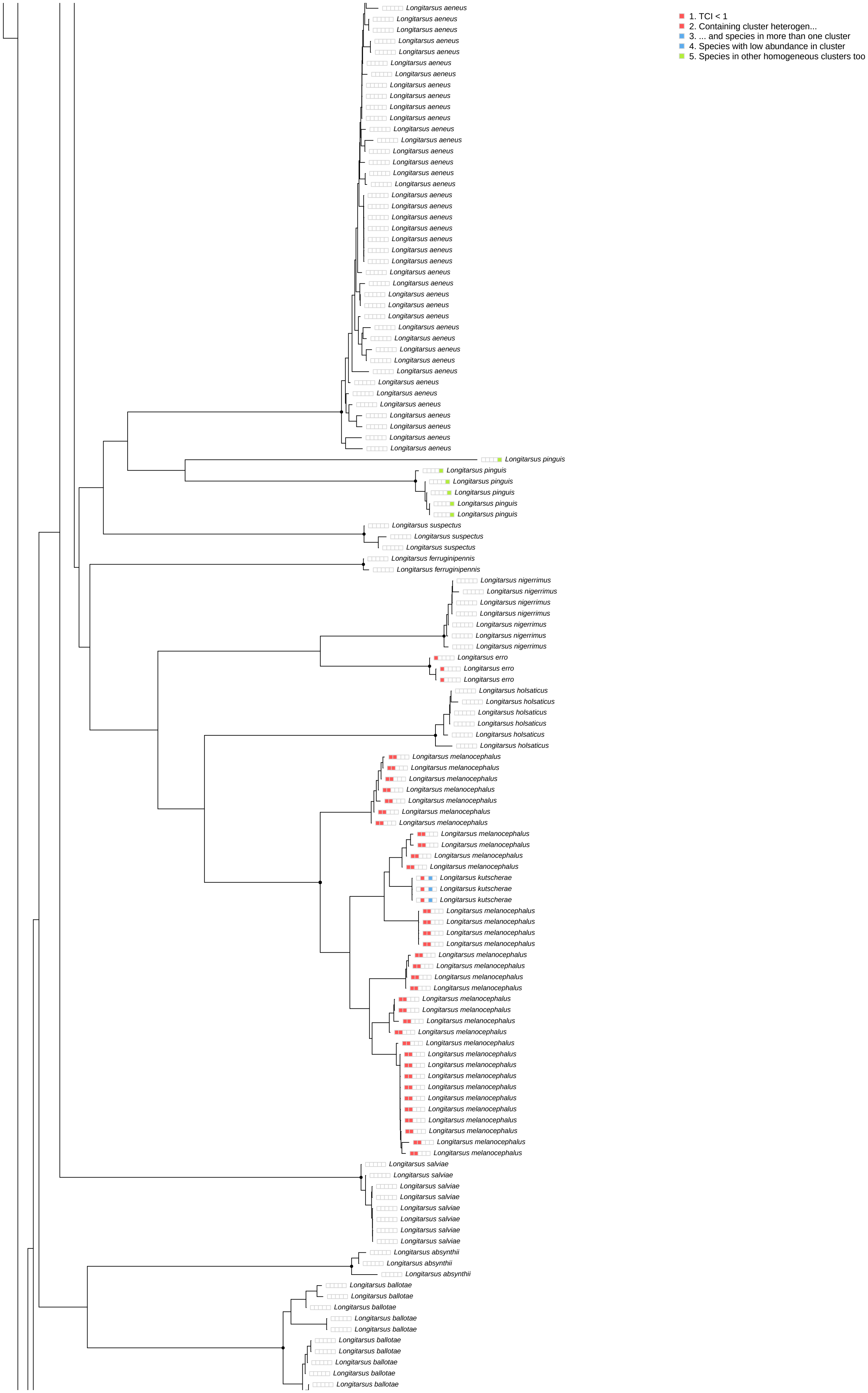
1. TCI < 1
2. Containing cluster heterogen...
3. ... and species in more than one cluster
4. Species with low abundance in cluster
5. Species in other homogeneous clusters too
Longitarsus aeneus
Longitarsus aeneus
Longitarsus aeneus
Longitarsus aeneus
Longitarsus aeneus
Longitarsus aeneus
Longitarsus aeneus
Longitarsus aeneus
Longitarsus aeneus
Longitarsus aeneus
Longitarsus aeneus
Longitarsus aeneus
Longitarsus aeneus
Longitarsus aeneus
Longitarsus aeneus
Longitarsus aeneus
Longitarsus aeneus
Longitarsus aeneus
Longitarsus aeneus
Longitarsus aeneus
Longitarsus aeneus
Longitarsus aeneus
Longitarsus aeneus
Longitarsus aeneus
Longitarsus aeneus
Longitarsus aeneus
Longitarsus aeneus
Longitarsus aeneus
Longitarsus aeneus
Longitarsus aeneus
Longitarsus aeneus
Longitarsus aeneus
Longitarsus aeneus
Longitarsus aeneus
Longitarsus aeneus
Longitarsus aeneus
Longitarsus aeneus
Longitarsus aeneus
Longitarsus aeneus
Longitarsus aeneus
Longitarsus aeneus
Longitarsus pinguis
Longitarsus pinguis
Longitarsus pinguis
Longitarsus pinguis
Longitarsus pinguis
Longitarsus pinguis
Longitarsus suspectus
Longitarsus suspectus
Longitarsus suspectus
Longitarsus ferruginipennis
Longitarsus ferruginipennis
Longitarsus nigerrimus
Longitarsus nigerrimus
Longitarsus nigerrimus
Longitarsus nigerrimus
Longitarsus nigerrimus
Longitarsus nigerrimus
Longitarsus nigerrimus
Longitarsus erro
Longitarsus erro
Longitarsus erro
Longitarsus holsaticus
Longitarsus holsaticus
Longitarsus holsaticus
Longitarsus holsaticus
Longitarsus holsaticus
Longitarsus holsaticus
Longitarsus melanocephalus
Longitarsus melanocephalus
Longitarsus melanocephalus
Longitarsus melanocephalus
Longitarsus melanocephalus
Longitarsus melanocephalus
Longitarsus melanocephalus
Longitarsus melanocephalus
Longitarsus melanocephalus
Longitarsus melanocephalus
Longitarsus melanocephalus
Longitarsus kutscherae
Longitarsus kutscherae
Longitarsus kutscherae
Longitarsus melanocephalus
Longitarsus melanocephalus
Longitarsus melanocephalus
Longitarsus melanocephalus
Longitarsus melanocephalus
Longitarsus melanocephalus
Longitarsus melanocephalus
Longitarsus melanocephalus
Longitarsus melanocephalus
Longitarsus melanocephalus
Longitarsus melanocephalus
Longitarsus melanocephalus
Longitarsus melanocephalus
Longitarsus melanocephalus
Longitarsus melanocephalus
Longitarsus melanocephalus
Longitarsus melanocephalus
Longitarsus melanocephalus
Longitarsus melanocephalus
Longitarsus melanocephalus
Longitarsus melanocephalus
Longitarsus melanocephalus
Longitarsus melanocephalus
Longitarsus salviae
Longitarsus salviae
Longitarsus salviae
Longitarsus salviae
Longitarsus salviae
Longitarsus salviae
Longitarsus salviae
Longitarsus salviae
Longitarsus absynthii
Longitarsus absynthii
Longitarsus absynthii
Longitarsus ballotae
Longitarsus ballotae
Longitarsus ballotae
Longitarsus ballotae
Longitarsus ballotae
Longitarsus ballotae
Longitarsus ballotae
Longitarsus ballotae
Longitarsus ballotae
Longitarsus ballotae

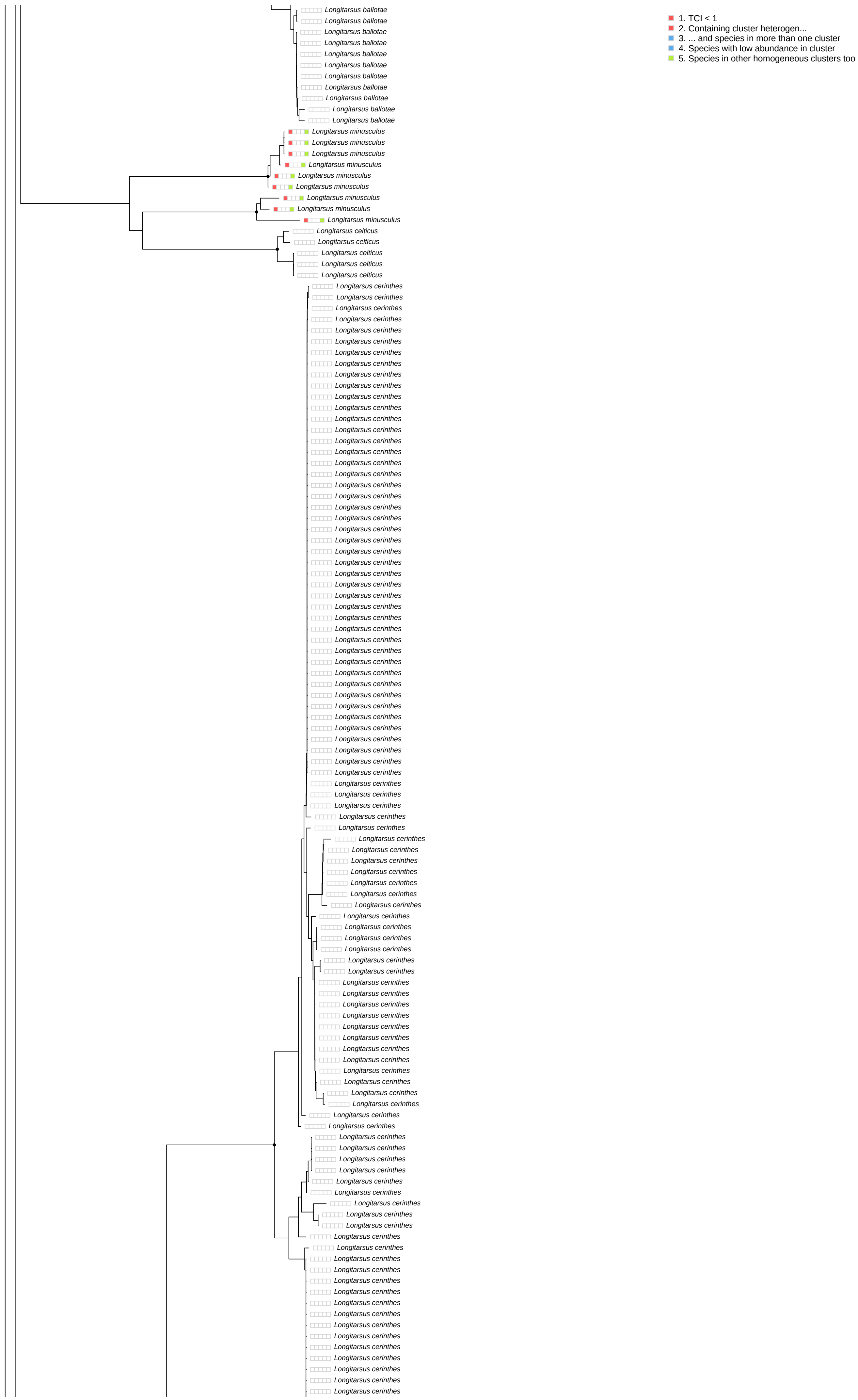
1. TCI < 1
2. Containing cluster heterogen...
3. ... and species in more than one cluster
4. Species with low abundance in cluster
5. Species in other homogeneous clusters too
Longitarsus ballotae
Longitarsus ballotae
Longitarsus ballotae
Longitarsus ballotae
Longitarsus ballotae
Longitarsus ballotae
Longitarsus ballotae
Longitarsus ballotae
Longitarsus ballotae
Longitarsus ballotae
Longitarsus ballotae
Longitarsus minusculus
Longitarsus minusculus
Longitarsus minusculus
Longitarsus minusculus
Longitarsus minusculus
Longitarsus minusculus
Longitarsus minusculus
Longitarsus minusculus
Longitarsus minusculus
Longitarsus celticus
Longitarsus celticus
Longitarsus celticus
Longitarsus celticus
Longitarsus celticus
Longitarsus cerinthes
Longitarsus cerinthes
Longitarsus cerinthes
Longitarsus cerinthes
Longitarsus cerinthes
Longitarsus cerinthes
Longitarsus cerinthes
Longitarsus cerinthes
Longitarsus cerinthes
Longitarsus cerinthes
Longitarsus cerinthes
Longitarsus cerinthes
Longitarsus cerinthes
Longitarsus cerinthes
Longitarsus cerinthes
Longitarsus cerinthes
Longitarsus cerinthes
Longitarsus cerinthes
Longitarsus cerinthes
Longitarsus cerinthes
Longitarsus cerinthes
Longitarsus cerinthes
Longitarsus cerinthes
Longitarsus cerinthes
Longitarsus cerinthes
Longitarsus cerinthes
Longitarsus cerinthes
Longitarsus cerinthes
Longitarsus cerinthes
Longitarsus cerinthes
Longitarsus cerinthes
Longitarsus cerinthes
Longitarsus cerinthes
Longitarsus cerinthes
Longitarsus cerinthes
Longitarsus cerinthes
Longitarsus cerinthes
Longitarsus cerinthes
Longitarsus cerinthes
Longitarsus cerinthes
Longitarsus cerinthes
Longitarsus cerinthes
Longitarsus cerinthes
Longitarsus cerinthes
Longitarsus cerinthes
Longitarsus cerinthes
Longitarsus cerinthes
Longitarsus cerinthes
Longitarsus cerinthes
Longitarsus cerinthes
Longitarsus cerinthes
Longitarsus cerinthes
Longitarsus cerinthes
Longitarsus cerinthes
Longitarsus cerinthes
Longitarsus cerinthes
Longitarsus cerinthes
Longitarsus cerinthes
Longitarsus cerinthes
Longitarsus cerinthes
Longitarsus cerinthes
Longitarsus cerinthes
Longitarsus cerinthes
Longitarsus cerinthes
Longitarsus cerinthes
Longitarsus cerinthes
Longitarsus cerinthes
Longitarsus cerinthes
Longitarsus cerinthes
Longitarsus cerinthes
Longitarsus cerinthes
Longitarsus cerinthes
Longitarsus cerinthes
Longitarsus cerinthes
Longitarsus cerinthes
Longitarsus cerinthes
Longitarsus cerinthes
Longitarsus cerinthes
Longitarsus cerinthes
Longitarsus cerinthes
Longitarsus cerinthes
Longitarsus cerinthes
Longitarsus cerinthes
Longitarsus cerinthes
Longitarsus cerinthes
Longitarsus cerinthes
Longitarsus cerinthes
Longitarsus cerinthes
Longitarsus cerinthes
Longitarsus cerinthes
Longitarsus cerinthes
Longitarsus cerinthes
Longitarsus cerinthes
Longitarsus cerinthes
Longitarsus cerinthes
Longitarsus cerinthes
Longitarsus cerinthes
Longitarsus cerinthes
Longitarsus cerinthes
Longitarsus cerinthes
Longitarsus cerinthes

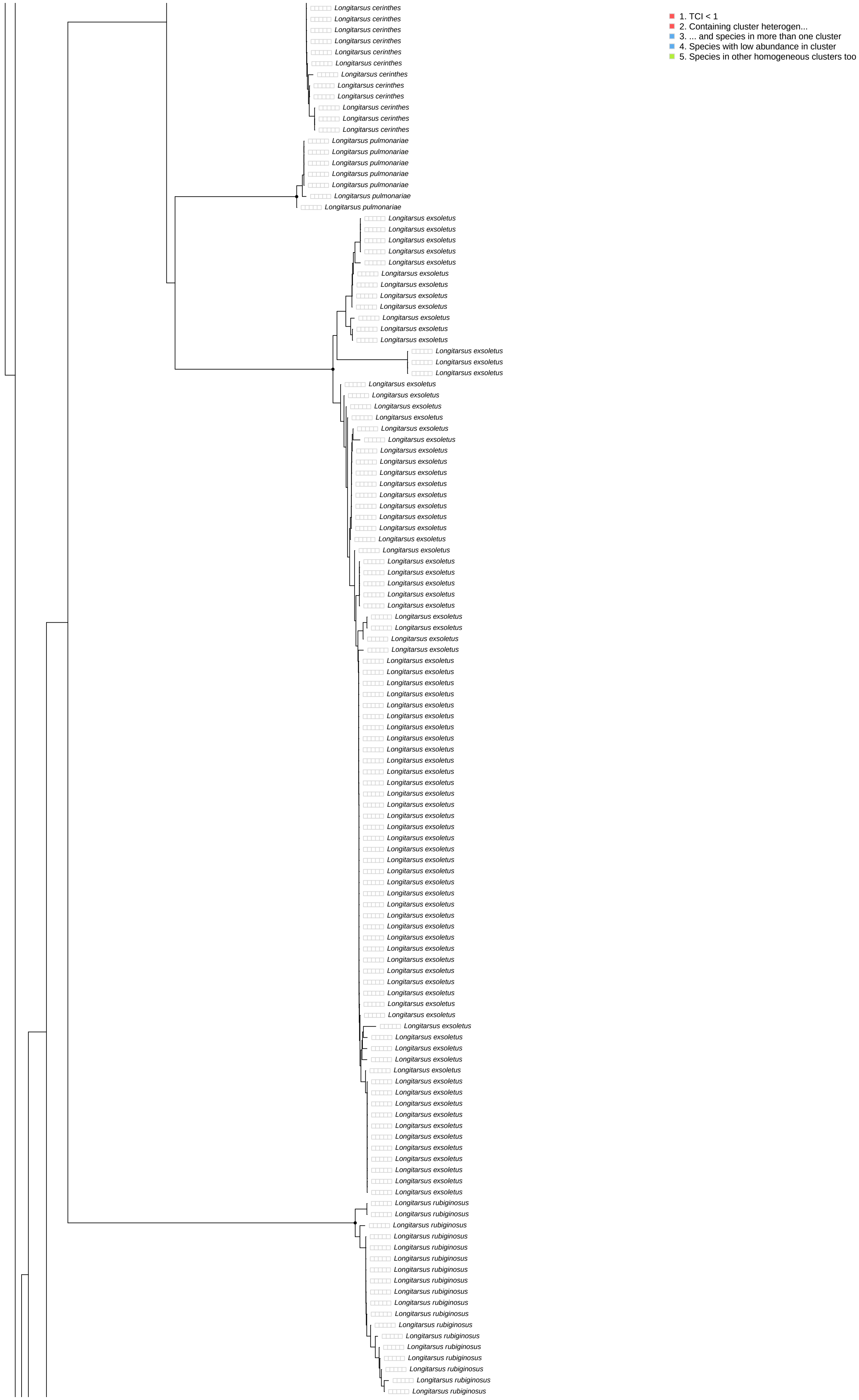
1. TCI < 1
2. Containing cluster heterogen...
3. ... and species in more than one cluster
4. Species with low abundance in cluster
5. Species in other homogeneous clusters too
Longitarsus cerinthes
Longitarsus cerinthes
Longitarsus cerinthes
Longitarsus cerinthes
Longitarsus cerinthes
Longitarsus cerinthes
Longitarsus cerinthes
Longitarsus cerinthes
Longitarsus cerinthes
Longitarsus cerinthes
Longitarsus cerinthes
Longitarsus cerinthes
Longitarsus pulmonariae
Longitarsus pulmonariae
Longitarsus pulmonariae
Longitarsus pulmonariae
Longitarsus pulmonariae
Longitarsus pulmonariae
Longitarsus pulmonariae
Longitarsus exsoletus
Longitarsus exsoletus
Longitarsus exsoletus
Longitarsus exsoletus
Longitarsus exsoletus
Longitarsus exsoletus
Longitarsus exsoletus
Longitarsus exsoletus
Longitarsus exsoletus
Longitarsus exsoletus
Longitarsus exsoletus
Longitarsus exsoletus
Longitarsus exsoletus
Longitarsus exsoletus
Longitarsus exsoletus
Longitarsus exsoletus
Longitarsus exsoletus
Longitarsus exsoletus
Longitarsus exsoletus
Longitarsus exsoletus
Longitarsus exsoletus
Longitarsus exsoletus
Longitarsus exsoletus
Longitarsus exsoletus
Longitarsus exsoletus
Longitarsus exsoletus
Longitarsus exsoletus
Longitarsus exsoletus
Longitarsus exsoletus
Longitarsus exsoletus
Longitarsus exsoletus
Longitarsus exsoletus
Longitarsus exsoletus
Longitarsus exsoletus
Longitarsus exsoletus
Longitarsus exsoletus
Longitarsus exsoletus
Longitarsus exsoletus
Longitarsus exsoletus
Longitarsus exsoletus
Longitarsus exsoletus
Longitarsus exsoletus
Longitarsus exsoletus
Longitarsus exsoletus
Longitarsus exsoletus
Longitarsus exsoletus
Longitarsus exsoletus
Longitarsus exsoletus
Longitarsus exsoletus
Longitarsus exsoletus
Longitarsus exsoletus
Longitarsus exsoletus
Longitarsus exsoletus
Longitarsus exsoletus
Longitarsus exsoletus
Longitarsus exsoletus
Longitarsus exsoletus
Longitarsus exsoletus
Longitarsus exsoletus
Longitarsus exsoletus
Longitarsus exsoletus
Longitarsus exsoletus
Longitarsus exsoletus
Longitarsus exsoletus
Longitarsus exsoletus
Longitarsus exsoletus
Longitarsus exsoletus
Longitarsus exsoletus
Longitarsus exsoletus
Longitarsus exsoletus
Longitarsus exsoletus
Longitarsus exsoletus
Longitarsus exsoletus
Longitarsus exsoletus
Longitarsus exsoletus
Longitarsus exsoletus
Longitarsus exsoletus
Longitarsus exsoletus
Longitarsus exsoletus
Longitarsus exsoletus
Longitarsus exsoletus
Longitarsus exsoletus
Longitarsus exsoletus
Longitarsus exsoletus
Longitarsus exsoletus
Longitarsus exsoletus
Longitarsus exsoletus
Longitarsus exsoletus
Longitarsus exsoletus
Longitarsus exsoletus
Longitarsus exsoletus
Longitarsus rubiginosus
Longitarsus rubiginosus
Longitarsus rubiginosus
Longitarsus rubiginosus
Longitarsus rubiginosus
Longitarsus rubiginosus
Longitarsus rubiginosus
Longitarsus rubiginosus
Longitarsus rubiginosus
Longitarsus rubiginosus
Longitarsus rubiginosus
Longitarsus rubiginosus
Longitarsus rubiginosus
Longitarsus rubiginosus
Longitarsus rubiginosus
Longitarsus rubiginosus
Longitarsus rubiginosus
Longitarsus rubiginosus

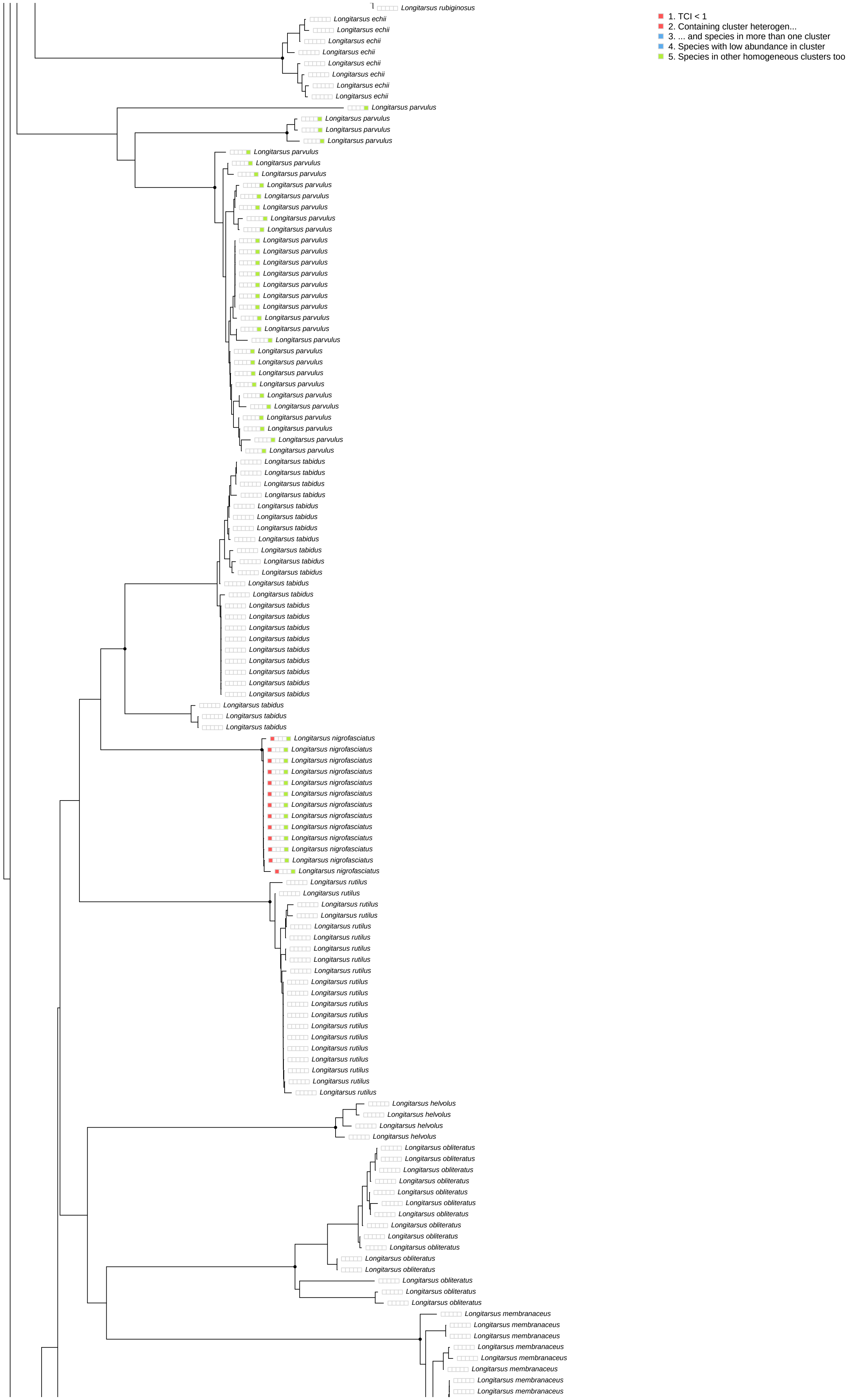
1. TCI < 1
2. Containing cluster heterogen...
3. ... and species in more than one cluster
4. Species with low abundance in cluster
5. Species in other homogeneous clusters too
Longitarsus rubiginosus
Longitarsus echii
Longitarsus echii
Longitarsus echii
Longitarsus echii
Longitarsus echii
Longitarsus echii
Longitarsus echii
Longitarsus echii
Longitarsus parvulus
Longitarsus parvulus
Longitarsus parvulus
Longitarsus parvulus
Longitarsus parvulus
Longitarsus parvulus
Longitarsus parvulus
Longitarsus parvulus
Longitarsus parvulus
Longitarsus parvulus
Longitarsus parvulus
Longitarsus parvulus
Longitarsus parvulus
Longitarsus parvulus
Longitarsus parvulus
Longitarsus parvulus
Longitarsus parvulus
Longitarsus parvulus
Longitarsus parvulus
Longitarsus parvulus
Longitarsus parvulus
Longitarsus parvulus
Longitarsus parvulus
Longitarsus parvulus
Longitarsus parvulus
Longitarsus parvulus
Longitarsus parvulus
Longitarsus parvulus
Longitarsus parvulus
Longitarsus parvulus
Longitarsus parvulus
Longitarsus parvulus
Longitarsus tabidus
Longitarsus tabidus
Longitarsus tabidus
Longitarsus tabidus
Longitarsus tabidus
Longitarsus tabidus
Longitarsus tabidus
Longitarsus tabidus
Longitarsus tabidus
Longitarsus tabidus
Longitarsus tabidus
Longitarsus tabidus
Longitarsus tabidus
Longitarsus tabidus
Longitarsus tabidus
Longitarsus tabidus
Longitarsus tabidus
Longitarsus tabidus
Longitarsus tabidus
Longitarsus tabidus
Longitarsus tabidus
Longitarsus tabidus
Longitarsus tabidus
Longitarsus tabidus
Longitarsus tabidus
Longitarsus nigrofasciatus
Longitarsus nigrofasciatus
Longitarsus nigrofasciatus
Longitarsus nigrofasciatus
Longitarsus nigrofasciatus
Longitarsus nigrofasciatus
Longitarsus nigrofasciatus
Longitarsus nigrofasciatus
Longitarsus nigrofasciatus
Longitarsus nigrofasciatus
Longitarsus nigrofasciatus
Longitarsus nigrofasciatus
Longitarsus nigrofasciatus
Longitarsus rutilus
Longitarsus rutilus
Longitarsus rutilus
Longitarsus rutilus
Longitarsus rutilus
Longitarsus rutilus
Longitarsus rutilus
Longitarsus rutilus
Longitarsus rutilus
Longitarsus rutilus
Longitarsus rutilus
Longitarsus rutilus
Longitarsus rutilus
Longitarsus rutilus
Longitarsus rutilus
Longitarsus rutilus
Longitarsus rutilus
Longitarsus rutilus
Longitarsus rutilus
Longitarsus rutilus
Longitarsus helvolus
Longitarsus helvolus
Longitarsus helvolus
Longitarsus helvolus
Longitarsus obliteratus
Longitarsus obliteratus
Longitarsus obliteratus
Longitarsus obliteratus
Longitarsus obliteratus
Longitarsus obliteratus
Longitarsus obliteratus
Longitarsus obliteratus
Longitarsus obliteratus
Longitarsus obliteratus
Longitarsus obliteratus
Longitarsus obliteratus
Longitarsus obliteratus
Longitarsus obliteratus
Longitarsus obliteratus
Longitarsus membranaceus
Longitarsus membranaceus
Longitarsus membranaceus
Longitarsus membranaceus
Longitarsus membranaceus
Longitarsus membranaceus
Longitarsus membranaceus
Longitarsus membranaceus

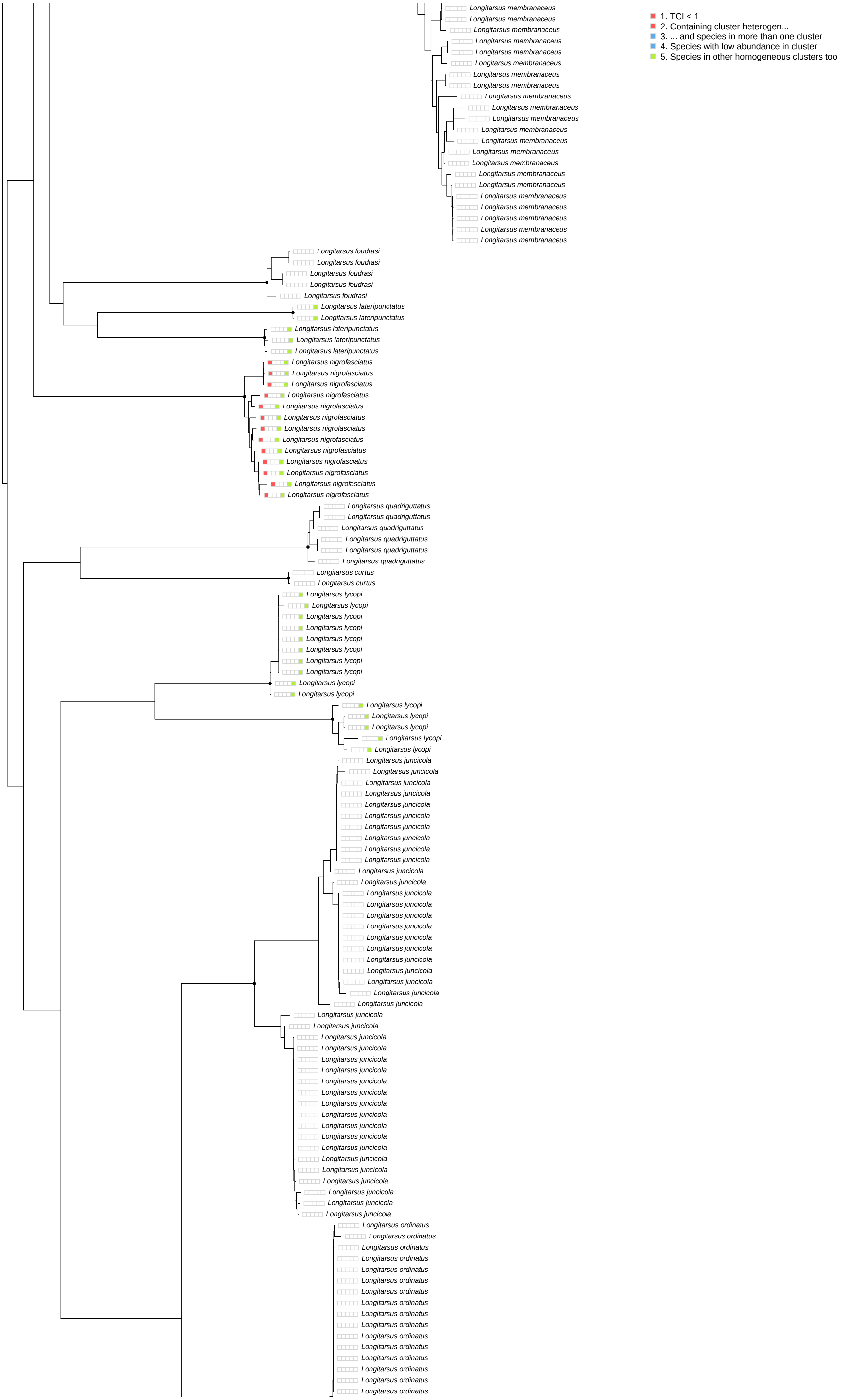
1. TCI < 1
2. Containing cluster heterogen...
3. ... and species in more than one cluster
4. Species with low abundance in cluster
5. Species in other homogeneous clusters too
Longitarsus membranaceus
Longitarsus membranaceus
Longitarsus membranaceus
Longitarsus membranaceus
Longitarsus membranaceus
Longitarsus membranaceus
Longitarsus membranaceus
Longitarsus membranaceus
Longitarsus membranaceus
Longitarsus membranaceus
Longitarsus membranaceus
Longitarsus membranaceus
Longitarsus membranaceus
Longitarsus membranaceus
Longitarsus membranaceus
Longitarsus membranaceus
Longitarsus membranaceus
Longitarsus membranaceus
Longitarsus membranaceus
Longitarsus membranaceus
Longitarsus membranaceus
Longitarsus membranaceus
Longitarsus foudrasi
Longitarsus foudrasi
Longitarsus foudrasi
Longitarsus foudrasi
Longitarsus foudrasi
Longitarsus lateripunctatus
Longitarsus lateripunctatus
Longitarsus lateripunctatus
Longitarsus lateripunctatus
Longitarsus lateripunctatus
Longitarsus nigrofasciatus
Longitarsus nigrofasciatus
Longitarsus nigrofasciatus
Longitarsus nigrofasciatus
Longitarsus nigrofasciatus
Longitarsus nigrofasciatus
Longitarsus nigrofasciatus
Longitarsus nigrofasciatus
Longitarsus nigrofasciatus
Longitarsus nigrofasciatus
Longitarsus nigrofasciatus
Longitarsus nigrofasciatus
Longitarsus nigrofasciatus
Longitarsus quadriguttatus
Longitarsus quadriguttatus
Longitarsus quadriguttatus
Longitarsus quadriguttatus
Longitarsus quadriguttatus
Longitarsus quadriguttatus
Longitarsus curtus
Longitarsus curtus
Longitarsus lycopi
Longitarsus lycopi
Longitarsus lycopi
Longitarsus lycopi
Longitarsus lycopi
Longitarsus lycopi
Longitarsus lycopi
Longitarsus lycopi
Longitarsus lycopi
Longitarsus lycopi
Longitarsus lycopi
Longitarsus lycopi
Longitarsus lycopi
Longitarsus lycopi
Longitarsus lycopi
Longitarsus juncicola
Longitarsus juncicola
Longitarsus juncicola
Longitarsus juncicola
Longitarsus juncicola
Longitarsus juncicola
Longitarsus juncicola
Longitarsus juncicola
Longitarsus juncicola
Longitarsus juncicola
Longitarsus juncicola
Longitarsus juncicola
Longitarsus juncicola
Longitarsus juncicola
Longitarsus juncicola
Longitarsus juncicola
Longitarsus juncicola
Longitarsus juncicola
Longitarsus juncicola
Longitarsus juncicola
Longitarsus juncicola
Longitarsus juncicola
Longitarsus juncicola
Longitarsus juncicola
Longitarsus juncicola
Longitarsus juncicola
Longitarsus juncicola
Longitarsus juncicola
Longitarsus juncicola
Longitarsus juncicola
Longitarsus juncicola
Longitarsus juncicola
Longitarsus juncicola
Longitarsus juncicola
Longitarsus juncicola
Longitarsus juncicola
Longitarsus juncicola
Longitarsus juncicola
Longitarsus juncicola
Longitarsus juncicola
Longitarsus juncicola
Longitarsus juncicola
Longitarsus ordinatus
Longitarsus ordinatus
Longitarsus ordinatus
Longitarsus ordinatus
Longitarsus ordinatus
Longitarsus ordinatus
Longitarsus ordinatus
Longitarsus ordinatus
Longitarsus ordinatus
Longitarsus ordinatus
Longitarsus ordinatus
Longitarsus ordinatus
Longitarsus ordinatus
Longitarsus ordinatus
Longitarsus ordinatus
Longitarsus ordinatus

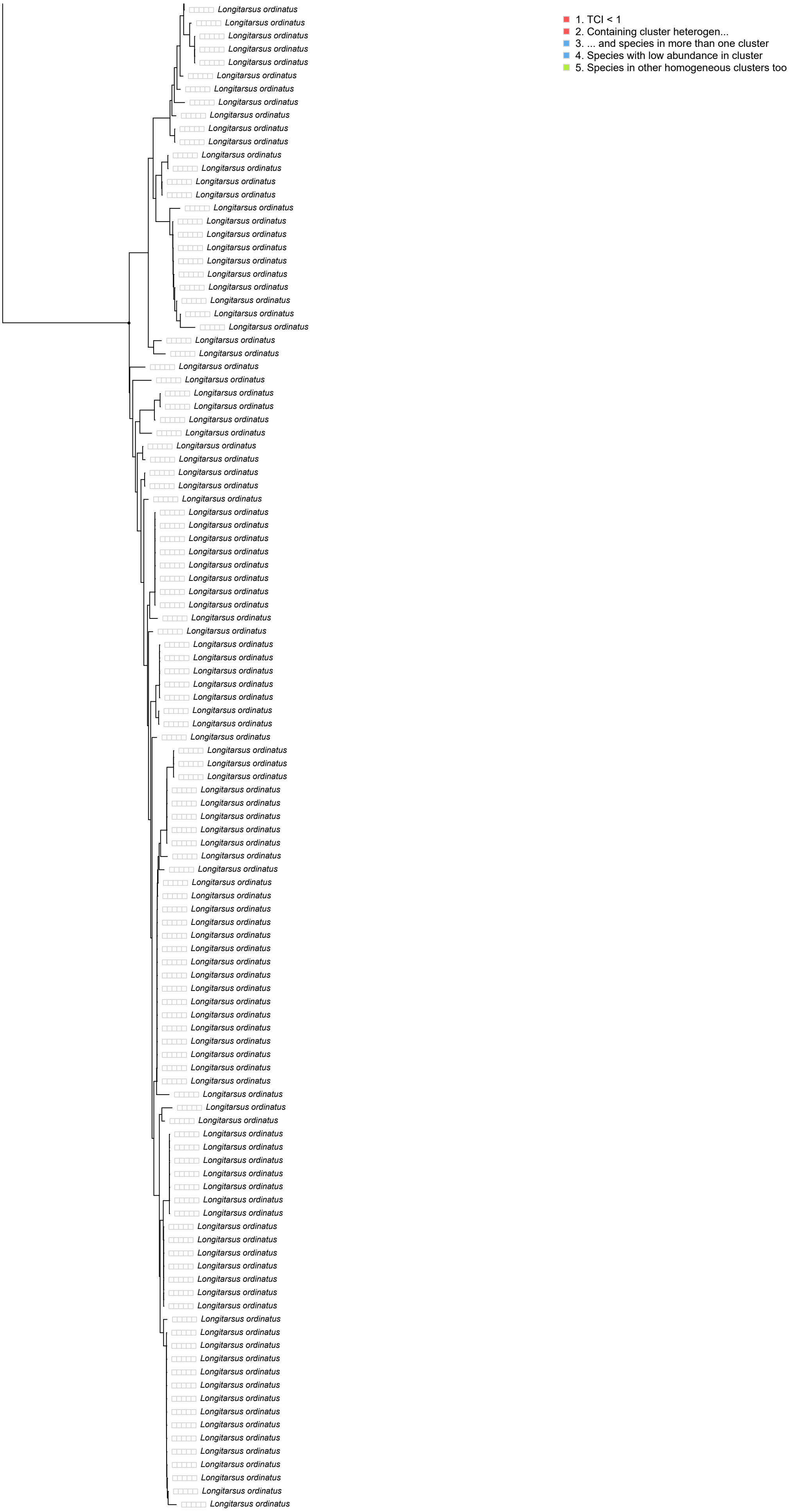

Supplementary Figure S8. Tree output from TaxCI analysis on the final dataset.
