# Supplementary material for: Sharpening the DNA barcoding tool through a posteriori taxonomic validation: The case of Longitarsus flea beetles (Coleoptera: Chrysomelidae)
Source: PLoS One. 2020 May 21;15(5):e0233573. doi: 10.1371/journal.pone.0233573 (PMC7241800; doi:10.1371/journal.pone.0233573)
Supplement: S1 Raw images — (PDF) [file pone.0233573.s011.pdf]

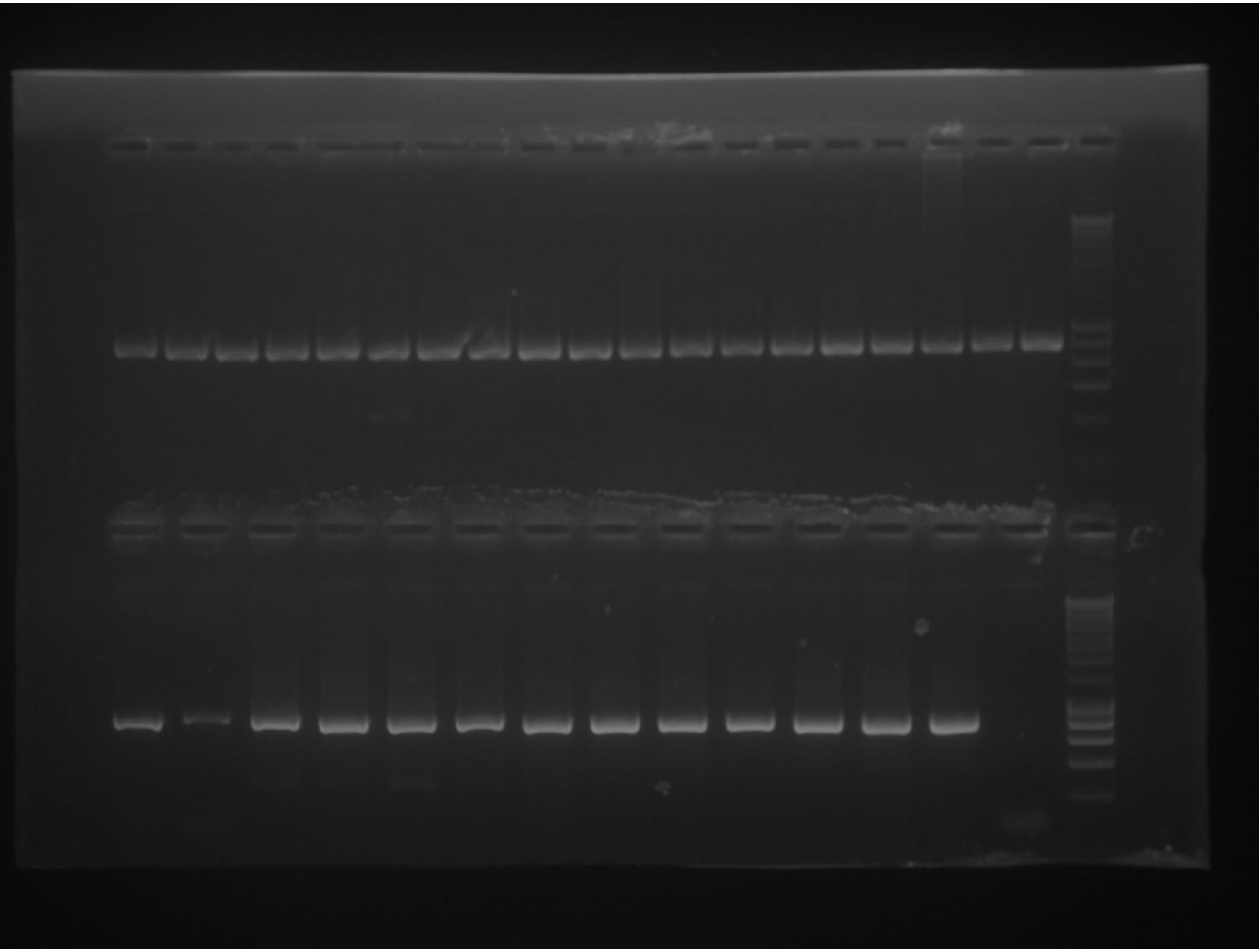

Image of the electrophoresis gel from which the Figure S5B (Supplementary materials) was generated. The image was taken with a Canon PowerShot G10. Rightmost lanes in the upper and lower part of the gel contain DNA ladder. Codes of the samples (see Table S2) uploaded in each lane (from left to right) of the upper part of the gel: 29b, 29c, 29d, 30a, 30b, 30c, 30d, 31a, 31b, 31c, 31d, 32a, 32b, 32c, 32d, 33a, 33b, 33c, 34a; lower part of the gel: 34b, 34c, 35a, 35b, 35c, 35d, 36a, 36b, 36c, 37a, 37b, 37c, X (sample not included in the analysis), empty.
